# Biosensor-based enzyme engineering approach applied to psicose biosynthesis

**DOI:** 10.1093/synbio/ysz028

**Published:** 2019-12-02

**Authors:** Jeremy Armetta, Rose Berthome, Antonin Cros, Celine Pophillat, Bruno Maria Colombo, Amir Pandi, Ioana Grigoras

**Affiliations:** 1 iSSB, UMR8030 Génomique Métabolique, Genoscope, Institut François Jacob, CEA, CNRS, Univ Evry, Université Paris-Saclay, Genopole Campus 1, Bât. 6, 5 rue Henri Desbruères, 91030 Evry, France; 2 Micalis Institute, INRA, AgroParisTech, Université Paris-Saclay, 78350, Jouy-en-Josas, France

**Keywords:** transcription factor-based biosensor, rare sugars, psicose, enzyme engineering, Universal Biosensing Chassis

## Abstract

Bioproduction of chemical compounds is of great interest for modern industries, as it reduces their production costs and ecological impact. With the use of synthetic biology, metabolic engineering and enzyme engineering tools, the yield of production can be improved to reach mass production and cost-effectiveness expectations. In this study, we explore the bioproduction of D-psicose, also known as D-allulose, a rare non-toxic sugar and a sweetener present in nature in low amounts. D-psicose has interesting properties and seemingly the ability to fight against obesity and type 2 diabetes. We developed a biosensor-based enzyme screening approach as a tool for enzyme selection that we benchmarked with the *Clostridium cellulolyticum* D-psicose 3-epimerase for the production of D-psicose from D-fructose. For this purpose, we constructed and characterized seven psicose responsive biosensors based on previously uncharacterized transcription factors and either their predicted promoters or an engineered promoter. In order to standardize our system, we created the Universal Biosensor Chassis, a construct with a highly modular architecture that allows rapid engineering of any transcription factor-based biosensor. Among the seven biosensors, we chose the one displaying the most linear behavior and the highest increase in fluorescence fold change. Next, we generated a library of D-psicose 3-epimerase mutants by error-prone PCR and screened it using the biosensor to select gain of function enzyme mutants, thus demonstrating the framework’s efficiency.

## 1. Introduction

For the last few decades, finding new solutions for sustainable production of valuable compounds and chemicals has been increasingly important. One of the most promising and efficient methods lies in harnessing the synthesis capabilities of engineered microbes. However, precise and robust engineering of these organisms remains challenging. Indeed, numerous steps of optimization are required for an implemented heterologous pathway to reach industrial synthesis capabilities and economical viability. Advances in the design have allowed generating millions of cell variants with different synthesis capabilities, but a major bottleneck resides in the screening and selection process. To help circumvent this hurdle, synthetic biology provides many valuable tools. Amongst these tools, biosensors have been extensively used for metabolic engineering with success in various organisms (1–5), but mainly bacteria and yeast. Overall, two types of biosensors are extensively used for metabolic engineering: transcription factor-based biosensors, relying on transcriptional regulators to sense metabolites (6) and RNA based biosensors, using riboswitches to trigger pathways in the presence of the desired compound (7, 8). However, transcription factor-based biosensors remain the most convenient to engineer (6) and have been successfully employed to detect amino acids (9–11), fatty acids (12, 13) or sugars (14–16), but also a large variety of other types of metabolites (17–21), directly or indirectly (22).

Indeed, metabolic engineering heavily contributes to sugar technologies. Sugar consumption and production remain a major environmental and societal problem. Recently, rare sugars, i.e. sugar occurring in small quantities in nature, have emerged as a potential solution (23). Indeed, rare sugars like D-allose, D-psicose, D-tagatose or L-xylose display numerous biological properties and could help fight obesity and type 2 diabetes, two diseases with dramatically increasing incidence in the population and for which the main factor linked with these pathologies is the over-consumption of sugar as well as high-fat diet. For example, D-psicose, also known as D-allulose, a C3 epimer of D-fructose is an ideal substitute for sucrose with around 70% of its sweetness. Thanks to a low absorption by the human gastrointestinal tract (24), D-psicose shows beneficial hypoglycemic and hypolipidemic properties for weight reduction and demonstrates important antioxidant activities (25, 26). In addition, D-psicose is also Generally Recognized As Safe (GRAS) by the U.S. Food and Drug Administration in June 2014 (GRAS Notice No. GRN 498) which allows its use for industrial food and beverage manufacturing as a sweetener. Therefore, achieving an efficient production of D-psicose could be very valuable. The rare sugar’s synthesis can be achieved chemically using organic synthesis, which proves to be a time consuming, and polluting process, inducing high manufacturing costs (27, 28). However, it is also possible to produce D-psicose through biocatalysis, but it remains highly challenging. This biocatalysis generally harnesses the ability of D-psicose 3-epimerases (DPEase) and D-tagatose 3-epimerases (DTEase) for bioconversion, by epimerization on the C3 position, of D-fructose into D-psicose. Numerous DPEase and DTEase have been reported, mainly from plant pathogens like *Pseudomonas cichorii* (29), *Agrobacterium tumefaciens* (30) or *Clostridium cellulolyticum* (31, 32). These enzymes could be good candidates for industrial biocatalysis, particularly the DPEase from the *C. cellulolyticum* for its thermal stability, but they demonstrate low enzymatic activity rendering costly all current industrial applications.

Here, we develop a framework to efficiently evolve and select for DPEase in order to improve its enzymatic activity, thereby enabling potentially significant production cost reduction. First, we designed seven different transcription factor-based biosensors to detect the D-psicose. We combined the use of PsiR, a predicted LacI family transcription factor with high affinity for D-psicose with both natural and synthetic inducible promoters. In order to efficiently build, test and optimize the different biosensor variants, we developed a Universal Biosensing Chassis (UBC). This synthetic construct optimized for Golden Gate assembly allowed a standardized, fast and reliable assembly of any transcription factor with its suitable inducible promoter. We then characterized each biosensor, regarding basal expression of fluorescence and responsive (operational) range, to assess which one would be the more suitable to screen for DPEase. The psicose biosensor based on the pPsiA promoter and PsiR transcription factor from *A.* *tumefaciens* demonstrated the best characteristics. Next, we engineered this biosensor to allow the insertion by Golden Gate assembly of a DPEase expression cassette into the biosensor vector. Using random mutagenesis and fluorescence-activated cell sorting (FACS), we generated and screened DPEase mutants displaying higher level of reporter production. Finally, we identified and characterized a *C. cellulolyticum* DPEase mutant, demonstrating the framework’s efficiency.

## 2. Materials and methods

### 2.1 Plasmid construction

*Escherichia coli* strain DH5α was used for cloning. pSB1C3 plasmid was used as backbone for all constructs. Transformed bacteria were selected on LB medium containing 35 µg/ml chloramphenicol.

All plasmids were assembled by the Golden Gate cloning method (33, 34). The T4 DNA ligase was purchased from New England Biolabs as well as the type IIS restriction endonucleases BsaI and BbsI. BsmBI was purchased from Thermo Fisher Scientific. DNA fragments were synthesized as gBlocks by Integrated DNA Technologies, Inc. (IDT) or amplified by polymerase chain reaction (PCR) with oligonucleotide primers bearing Golden Gate adapters at their 5′ ends (synthesized by IDT). PCR reactions were carried out using the Q5^®^ High-Fidelity DNA Polymerase (New England Biolabs) according to the manufacturer’s protocol. Error-prone PCR was performed according to the protocol described by Wilson and Keefe (35) using the OneTaq DNA Polymerase (New England Biolabs). Successful cloning was verified by sequencing (GATC Biotech, now Eurofins Genomics).

This work was initiated in the framework of the international Genetically Engineered Machines (iGEM) competition by the Evry Paris-Saclay 2017 team. Consequently, all nucleotide sequences were submitted to the publicly available iGEM’s Registry of Standard Biological Parts (http://parts.igem.org/). Sequence information about all individual functional parts (genes, promoters and terminators) are indicated in Supplementary Table S1. All plasmids accession numbers are listed in Table 1 and their sequences are available in GenBank format in the Supplementary material. All plasmids follow the BioBrick RFC[10] standard and are in the pSB1C3 backbone. The details of the construction of each plasmid including the sequences of all primers used for PCR and all gBlocks can be found in the Supplementary Materials and Methods section.

**Table 1.** Plasmids build and used in this study

Accession numbers	Description
BBa_K2448023	Universal Biosensing Chassis (UBC)
BBa_K2448025	Psicose biosensor based on pPsiA promoter from *A. tumefaciens* and the PsiR transcription factor from *A. tumefaciens* with mCherry as reporter gene
BBa_K2448026	Psicose biosensor based on pPsiR promoter from *A. tumefaciens* and the PsiR transcription factor from *A. tumefaciens* with mCherry as reporter gene
BBa_K2448027	Psicose biosensor based on pPsiTacI synthetic promoter and the PsiR transcription factor from *A. tumefaciens* with mCherry as reporter gene
BBa_K2448028	Psicose biosensor based on pPsiA promoter from *S. fredii* and the PsiR transcription factor from *S. fredii* with mCherry as reporter gene
BBa_K2448029	Psicose biosensor based on pPsiR promoter from *S. fredii* and the PsiR transcription factor from *S. fredii* with mCherry as reporter gene
BBa_K2448030	Psicose biosensor based on pPsiA promoter from *S. meliloti* and the PsiR transcription factor from *S. meliloti* with mCherry as reporter gene
BBa_K2448031	Psicose biosensor based on pPsiR promoter from *S. meliloti* and the PsiR transcription factor from *S. meliloti* with mCherry as reporter gene
BBa_K2448057	Psicose biosensor based on pPsiA promoter from *A. tumefaciens* and the PsiR transcription factor from *A. tumefaciens* with mEmerald as reporter gene and a downstream the Mutant Drop Zone
BBa_K2448058	Psicose biosensor based on pPsiA promoter from *A. tumefaciens* and the PsiR transcription factor from *A. tumefaciens* with mEmerald as reporter gene and a downstream D-Psicose 3-epimerase (DPEase) from *C. cellulolyticum* under the control of pTacI promoter
BBa_K2448033	D-Psicose 3-epimerase (DPEase) from *C. cellulolyticum* under the control of pTacI promoter
BBa_K2448054	D-Psicose 3-epimerase (DPEase) from *C. cellulolyticum* with a C-terminal Histidine tag under the control of pTacI promoter

### 2.2 Biosensor *in vivo* characterization

The pSB1C3 plasmids harboring the psicose biosensors were introduced into *E. coli* DH5α. Transformed cells were grown overnight at 37°C in LB medium containing 35 µg/ml chloramphenicol. The suspension was diluted by 100 in the same medium and incubated at 37°C and 200 rpm for 1 h. Afterwards, a 96 well plate (COSTAR^®^ 3603, Corning Inc.) was prepared and each well was filled with 120 µl of cell suspension and 30 µl of a solution containing Psicose and IPTG. Different concentrations of Psicose (0, 0.1 µM, 1 µM, 10 µM, 100 µM, 1 mM, 10 mM, 100 mM, 200 mM and 300 mM) and IPTG (0, 1, 10, 100 and 1000 µM) were tested. The plate was incubated at 37°C at 200 rpm, fluorescence and OD_600nm_ were measured every 7 min during 150 cycles. Fluorescence of mCherry was measured using CLARIOstar^®^ plate reader (BMG Labtech) at 587/610 nm, the mCherry wavelengths of fluorescence excitation and emission (36). Fluorescence of mEmerald was measured using Synergy™ HTX plate reader (BioTek^®^ Instruments, Inc.) at 485/528 nm, the mEmerald wavelengths of fluorescence excitation and emission (37). The experiments were performed in triplicate and the fluorescence values (background subtracted) normalized by cell density (OD_600nm_).

### 2.3 Fluorescence-activated cell sorting

A library of DPEase of *C. cellulolyticum* mutants was generated following the error-prone PCR protocol (35) using the OneTaq DNA Polymerase (New England Biolabs), the forward primer 5′-GCCGTCTCGGATGAAACACGGTATCTACTAC-3′, the reverse primer 5′-GCCGTCTCCCGCTTTAAGAGTGTTTGTGGCATTC-3′ and as template a gBlock encoding the *C. cellulolyticum* DPEase. A control library was performed with the Q5^®^ High-Fidelity DNA Polymerase (New England Biolabs). Each library was inserted in the Mutant Drop Zone (MDZ) downstream of the psicose biosensor (BBa_K2448057) by Golden Gate, using the BsmBI restriction enzyme (Thermo Fisher Scientific). Ten microliters of the Golden Gate reaction were used to transform chemically competent *E. coli* DH5α cells. After overnight culturing in LB media supplemented with 35 µg/ml chloramphenicol, transformed cells were centrifuged, washed with IsoFlow Sheath Fluid (Beckman Coulter) and resuspended in this same isotonic fluid at a concentration of 10^6^ cells/ml. Flow cytometric measurements were performed at Genoscope on a MoFlo Astrios cell sorter (Beckam Coulter), using a single laser operating at 488 nm for excitation and a channel of 576/21 nm for detection of the mEmerald fluorescence. The selection was triggered by fluorescence (threshold 0.05%). The data were analyzed using the Summit V6.2 Software (Beckam Coulter).

### 2.4 Bioproduction of psicose from fructose

The pSB1C3 plasmids harboring the DPEase under the control of pTacI promoter (BBa_K2448033) were introduced into *E. coli* BL21-AI (New England Biolabs). Transformed cells were grown at 37°C in mineral salts medium (38, 39) (7 g/l K_2_HPO_4_, 3 g/l KH_2_PO_4_, 1 g/l (NH_4_)_2_SO_4_, 2 µM FeSO_4_, 0.4 mM MgSO_4_, 1.44 mM sodium citrate, 0.1 mg/l Thiamine, 2 g/l glucose) containing 35 µg/ml chloramphenicol. When cells reached early/middle exponential growth phase (OD_600nm_ = 0.6), protein expression was induced with 1 mM isopropyl ß-D-thiogalactopyranoside (IPTG) and the media was supplemented with fructose at various concentrations. Cultures were sampled every 2 h and, after centrifugation at high speed, the supernatant was analyzed by high performance liquid chromatography (HPLC).

### 2.5 HPLC analysis

HPLC analysis was carried out using a Shimadzu Prominence LC20/SIL-20AC equipped with a SUPELCOGEL™ Ca column (300 × 7.8 mm, 9 μm particle size, 6% Crosslinked) and a RID-10A refractive index detector. The separation was performed isocratically using pure water as mobile phase, at a flow rate of 500 µl/min on the column thermostated at 85°C. The sample injection volume was 20 µl. Quantification of sugars was done by interpolation of the integrated peak areas using a calibration curve prepared with standard samples.

### 2.6 Purification of DPEase under native conditions

The pSB1C3 plasmids harboring the His-tagged DPEase variants under the control of pTacI promoter (BBa_K2448054) were introduced into *E. coli* BL21-AI (New England Biolabs). Transformed cells were grown at 37°C in 50 ml LB medium containing 35 µg/ml chloramphenicol. When cells reached early/middle exponential growth phase (OD_600nm_ = 0.6), protein expression was induced with 1 mM isopropyl ß-D-thiogalactopyranoside (IPTG). After overnight culture, cells were harvested by centrifugation at 5000 g for 30 min at 4°C. Cell pellet was resuspended in 2 ml Lysis Buffer containing 50 mM Tris–HCl Buffer pH 7.5, 100 mM NaCl, 10 µg/ml lysozyme, 1 mM phenylmethylsulfonyl fluoride, 10 µg/ml DNase and 10 µg/ml RNase. Cells were broken with 1 g of glass beads by vortexing three times 1 min at maximum speed interrupted by 1 min on ice. Debris was removed by centrifugation (14 000 g for 20 min at 4°C) and the supernatant collected. Purification of DPEase was performed essentially as described (31) using the Ni-NTA Spin kit (Qiagen). Briefly, the column was equilibrated with 600 µl Equilibration Buffer (50 mM Tris–HCl Buffer pH 7.5, 500 mM NaCl), then 1.2 ml of crude soluble lysate from *E. coli* cells were loaded. After washing twice with 600 µl of Equilibration Buffer, then twice with 600 µl Wash Buffer (50 mM Tris–HCl Buffer pH 7.5, 500 mM NaCl, 50 mM Imidazole), the target protein was eluted with 3 × 600 µl Elution Buffer (50 mM Tris–HCl Buffer pH 7.5, 500 mM NaCl, 500 mM Imidazole). All manipulations were performed at 4°C. Protein purification was visualized by sodium dodecyl sulfate polyacrylamide gel electrophoresis (SDS-PAGE). Protein samples (10 µl) to be analyzed by SDS-PAGE were mixed with Laemmli Buffer (final concentrations 20.83 mM Tris–HCl pH 6.8, 0.67% (w/v) SDS, 3.33% glycerol, 1.67% 2-mercaptoéthanol, 0.5% bromophenol blue) and after heating for 3 min at 95°C, they were loaded onto a 12% SDS-polyacrylamide gel for protein separation, using a Bio-Rad Protean mini-gel system. Electrophoresis was performed in the SDS-PAGE running buffer (3.03 g/l Tris base, 14.4 g/l Glycine, 1 g/l SDS, pH 8.3) at constant 200 V, until the dye migrated close to the bottom of the gel. The gel was then stained with Coomassie Blue R-250. The total amount of proteins was determined by Bradford protein assay using the Pierce™ Coomassie Plus Assay Kit (Thermo Scientific™) following the manufacturer’s instructions for the Micro Test protocol. Briefly, the protein solution was mixed to an equal volume of 1× dye reagent and the absorbance was measured at 595 nm after 5 min of incubation at room temperature. A calibration curve was created using a set of bovine serum albumin dilutions with concentrations ranging from 0 to 25 µg/ml.

### 2.7 Enzyme activity

Initial rates of DPEase activity were assayed essentially as described (31) at 55°C in 50 mM Tris–HCl pH 8.0 containing 7.5 μg/ml protein, 0.1 mM CoCl_2_ and up to 100 g/l substrate D-fructose. The reactions were stopped by boiling and analyzed by HPLC. Data were fitted to the Michaelis–Menten equation using least-squares non-linear regression to generate estimates of K_m_ and k_cat_ values.

*Material sharing statement*. The materials and resources described in this article are available from the corresponding author on reasonable request.

## 3. Results and discussion

### 3.1 Design–build–test of seven psicose biosensors

**Figure 1.**
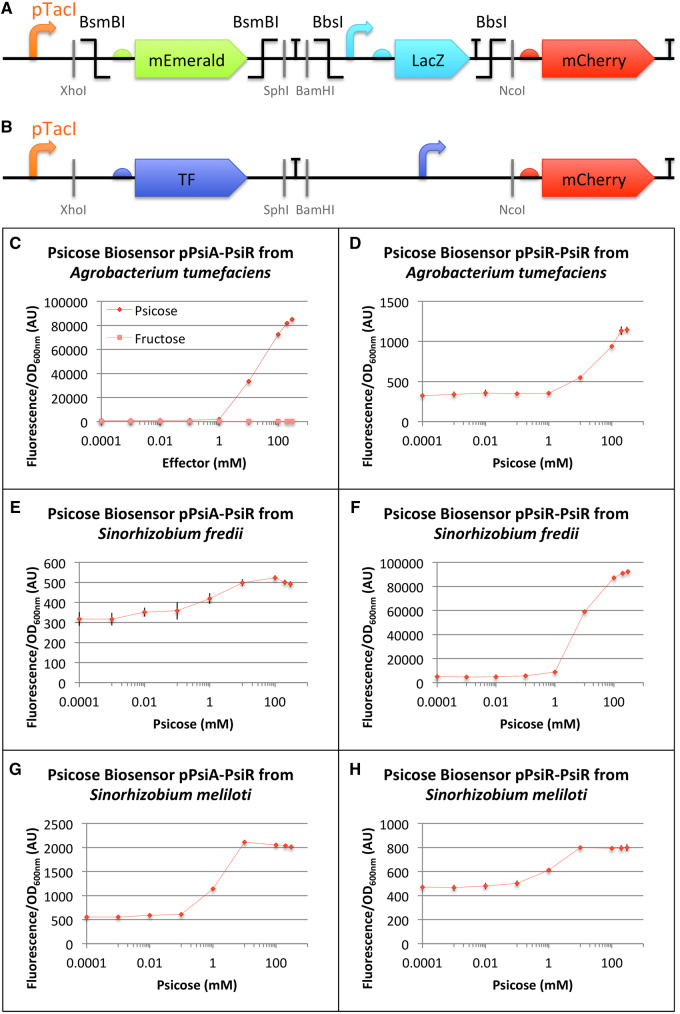
Design and characterization of six psicose biosensors. (**A**) Schematic representation of the UBC used as a platform to build the psicose biosensors (**B**). (**C–H**) *In vivo* characterization of mCherry expression by *E. coli* cells harboring (C) the psicose biosensor based on pPsiA promoter from *A. tumefaciens* and the PsiR transcription factor from *A. tumefaciens* (BBa_K2448025), (D) the psicose biosensor based on pPsiR promoter from *A. tumefaciens* and the PsiR transcription factor from *A. tumefaciens* (BBa_K2448026), (E) the psicose biosensor based on pPsiA promoter from *S. fredii* and the PsiR transcription factor from *S. fredii* (BBa_K2448028), (F) the psicose biosensor based on pPsiR promoter from *S. fredii* and the PsiR transcription factor from *S. fredii* (BBa_K2448029), (G) the psicose biosensor based on pPsiA promoter from *S. meliloti* and the PsiR transcription factor from *S. meliloti* (BBa_K2448030), (H) the psicose biosensor based on pPsiR promoter from *S. meliloti* and the PsiR transcription factor from *S. meliloti* (BBa_K2448031). Fluorescence values (background subtracted) were normalized by OD_600nm_. The data and error bars are the mean and standard deviation of six measurements (three biological replicates, each measured as two technical duplicates).

To achieve a precise and rapid engineering of transcription factor-based biosensors, we first designed the UBC (Figure 1A) that allows two different assembly methods for the insertion of transcription factors and promoters: the Golden Gate assembly (33, 34), or the traditional digestion–ligation. UBC contains insertion markers in order to enable quick and easy identification of the colonies carrying the right construct: mEmerald (37) for the transcription factors and LacZ-alpha for the promoters. An inducible pTacI promoter controls the transcription factor expression in the chassis, and we selected strong RBSs and efficient synthetic terminators to regulate the overall transcription and translation in the chassis. Finally, we used mCherry (36) as a reporter. This monomeric fluorescent protein shows rapid maturation, low brightness as well as an improved photostability and resistance to bleaching which makes it the perfect reporter for precise measurements. Moreover, unlike green fluorescent protein-like proteins, there is no *E. coli* cell auto-fluorescence effect at its excitation wavelength.

To construct a variety of psicose biosensors, using the UBC architecture, it was essential to identify a transcription factor with a high affinity to D-psicose. Using the SensiPath tool (40), we identified PsiR of *Rhizobiales* that appeared to be a great candidate. It is a predicted LacI family transcription factor with high affinity for D-psicose. This implies that PsiR is potentially capable of binding a consensus sequence in the promoter region and prevent transcription of the regulated promoters in the absence of D-psicose, in a manner similar to the way LacI does in the absence of allolactose (or the synthetic IPTG). PsiR occurs naturally in different *Rhizobiales* species (*A.* *tumefaciens*, *Sinorhizobium fredii*, *Sinorhizobium meliloti*) where it regulates an operon while also self-regulating its own expression. In all these species, the genetic context is similar as illustrated in Supplementary Figure S1: *psiR* gene precedes an operon starting with the *psiA* gene, but faces in the opposite direction, meaning that the promoter regions of *psiA* and *psiR* are overlapping. Furthermore, using the BPROM webserver (41), we identified two -35 and -10 boxes in close proximity to two 20 bp sequences conserved between different Rhizobiales species and that could be the PsiO sequences, with a function equivalent to the LacO sequences of the lactose operon. These regulatory regions could be great candidates for a PsiR regulated promoter, regulating the transcription of *mCherry*. Thus, the 400 bp sequences upstream of *psiA* and *psiR* were extracted from the genome of each species, to generate two promoter regions that are denoted pPsiA and pPsiR, respectively.

Using the UBC (Figure 1A), six different biosensors were generated by replacing the mEmerald with one of the three codon-optimized PsiR of *A. tumefaciens*, *S. fredii* or *S. meliloti* and *lacZ* with a corresponding pPsiR or pPsiA from the same species (Figure 1B). The six D-psicose biosensors should work in the following way: when pTacI is induced by IPTG, it drives the transcription of *psiR gene encoding* the PsiR protein that is predicted to be a transcription factor able to bind D-psicose. If D-psicose is present in the cell, PsiR will bind preferentially to it and thus this transcription factor becomes inactivated. The repression of the related promoter pPsi will be released which will enable the expression of a fluorescent protein, mCherry. If D-psicose is not present in the cell, PsiR will bind to pPsi, preventing any expression of mCherry.

To determine which of the six constructed biosensors were the most suited for our screening process, *E. coli* cultures were transformed with individual biosensors and characterized using a plate reader. By measuring the fluorescence intensity of the mCherry protein, normalized to the cell density, critical parameters were evaluated such as the optimal measurement time, the basal expression and the responsive range (Figure 1 C–H and Supplementary Figure S2). The optimal measurement time, which is the shortest time to get an observable signal for each biosensor, was assessed using a range of D-psicose concentrations. It turned out that for the majority of our biosensors, if D-psicose concentrations were above 10 mM, a 9 hours’ incubation after induction would give relevant results. The basal activity of biosensors corresponds to the signal emitted in the absence of D-psicose, which is due to the imbalance between the amount of PsiR transcription factor available and the pPsi promoter strength. Even when PsiR is produced, the transcription factor cannot totally prevent the transcription of the *mCherry* gene. A biosensor with a low basal activity could seem favorable; however, it is often related to lower sensing abilities. This parameter is therefore not sufficient in itself and should be associated with other criteria. For a biosensor characterization, the fold change of fluorescence is more interesting than the absolute intensity (Supplementary Figure S2). The sensitivity of a biosensor is determined when a significant change in the fluorescence intensity can be measured in relation to D-psicose concentration. For our biosensors, we can observe that a signal arises from the basal signal around 1 mM (Figure 1 C–H and Supplementary Figure S2). The different versions of the biosensor are also saturated around a concentration of 300 mM of D-psicose. The span of concentrations between the detection and the saturation is reflected by the responsive range, which is essential to evaluate to which range of concentration our biosensor can be used to give a significant output.

From these results, we can see that each PsiR behaved as predicted, inhibiting the pPsi promoters and interacting with D-psicose. Their responsive ranges are similar, ranging from 1 mM to 300 mM of D-psicose. The difference appears in the fold change and the linearity profile of the response (the fluorescence fold change being the ratio of the fluorescence values when 300 or 0 mM of D-psicose are added). The biosensor based on pPsiA and PsiR from *A. tumefaciens* shows both high fold change (90.4 ± 1.4×) and linearity in the range of concentrations corresponding to those of the bioproduction (1–300 mM of D-psicose) (Figure 1C and Supplementary Figure S2A, H). The biosensor based on pPsiR and PsiR from *A. tumefaciens* shows saturation at high concentrations but also a weak fold change (3.4 ± 1.1×), making it not suitable for an enzyme improvement (Figure 1D and Supplementary Figure S2B, H). The biosensors based on pPsiA and PsiR from *S. fredii* and on the pPsiR and PsiR from *S. meliloti* show similar characteristics with an early saturation upon increasing the concentration and a very low fold change (1.5 ± 0.05× and 1.7 ± 0.04×, respectively) making them bad candidates even if they display great sensitivity (Figure 1E, H and Supplementary Figure S2C, F, H). The biosensor based on pPsiR and PsiR from *S. fredii* displays a high fold change (20.3 ± 0.3×) but it tends to saturate at high concentrations (Figure 1F and Supplementary Figure S2D, H). This biosensor is still suitable for screening. Finally, the biosensor based on pPsiA and PsiR from *S. meliloti* is not suitable because of an early saturation with increasing concentration of D-psicose combined with a very low fold change (3.7 ± 0.1×) (Figure 1G and Supplementary Figure S2E, H).

The biosensor based on pPsiA and PsiR from *A. tumefaciens* is the best candidate because of its linearity and fold change, but it also has to work in D-psicose bioconversion conditions. The PsiR from *A. tumefaciens* has to specifically respond to its ligand and not to other molecules of the cell or the media, such as D-fructose, which will be at high concentration. Using the same range of concentrations of D-fructose, on the pPsiA-PsiR biosensor from *A. tumefaciens* we can see that D-fructose does not influence the biosensor behavior since mCherry production isn’t a function of fructose concentration in the media (Figure 1C and Supplementary Figure S2A). This finding implies that our transcription factor does not bind to D-fructose and that it can be used in high fructose level media to measure psicose concentration. Therefore, the pPsiA-PsiR biosensor from *A. tumefaciens* is suitable for assessing the activity of D-psicose 3-epimerase converting D-fructose into D-psicose.

**Figure 2.**
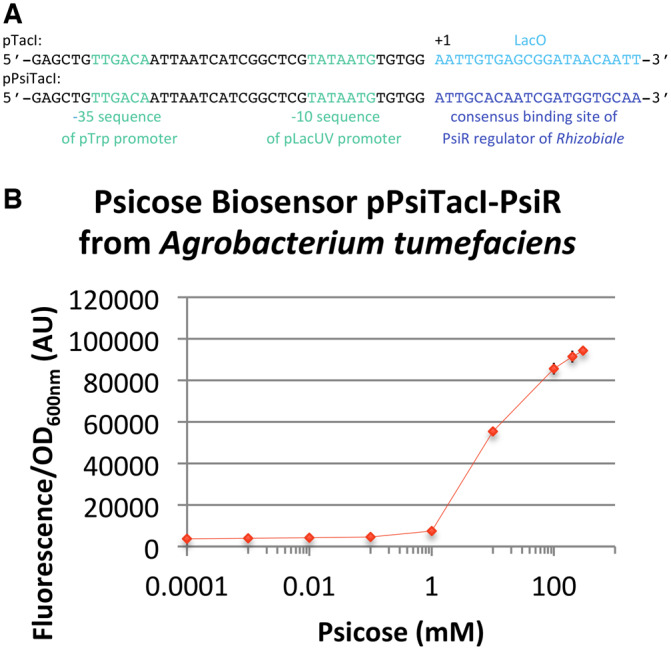
Design and characterization of a synthetic psicose biosensor. (**A**) Sequence comparison between the pTacI promoter (43) and the pPsiTacI synthetic promoter. (**B**) *In vivo* characterization of mCherry expression by *E. coli* cells harboring the psicose biosensor based on pPsiTacI synthetic promoter and the PsiR transcription factor from *A. tumefaciens* (BBa_K2448027). Fluorescence values (background subtracted) were normalized by OD_600nm_. The data and error bars are the mean and standard deviation of six measurements (three biological replicates, each measured as two technical duplicates).

The results presented in Figure 1 show that all pPsiR and pPsiA natural promoters are active in *E. coli* and that are regulated by the corresponding PsiR and by the presence of D-psicose. Knowing that PsiR is a LacI family transcription factor, and that these transcription factors modulate the expression of regulated genes by binding to a specific operator DNA sequence (42), we decided to further characterize this inducible system by engineering a hybrid synthetic promoter. We have based this hybrid synthetic promoter on the well-known LacI regulated promoter, pTacI (43) and we replaced the LacO sequence of pTacI by a consensus 20 bp sequence on which PsiR is predicted to bind according to RegPrecise database. The thus newly created promoter region, pPsiTacI (Figure 2A) combined with the PsiR from *A. tumefaciens* led to the seventh D-psicose biosensor which displays the same responsive range as the other six psicose biosensors described above, a high fold change (24.7 ± 0.6×) and a satisfactory linearity (Figure 2B and Supplementary Figure S2G, H). pPsiTacI behaved as predicted being tightly regulated by PsiR thanks to the 20 bp consensus sequence.

To the best of our knowledge, the results we present in Figures 1 and 2 are a first proof that PsiR is a transcription factor that negatively regulates the pPsi promoters in the absence of D-psicose and which, in the presence of D-psicose, allows the expression of a gene placed under the control of the pPsi promoter. The regulation is dependent on a 20 bp sequence (Figure 2) present in pPsi to which PsiR (potentially) binds. This sequence was sufficient to change the induction specificity of a LacI regulated promoter (pTacI) and convert it into a psicose inducible promoter. The seven psicose biosensors allowed us to develop a set of seven psicose inducible promoters with variable strengths, working in a widely used chassis *E. coli* and that allow fine-tuning of gene expression levels with applications that go beyond the scope of this article.

### 3.2 Bioproduction of D-psicose from D-fructose

In order to improve the bioconversion of D-fructose into D-psicose, we decided to engineer the DPEase from *C. cellulolyticum* (31) and screen mutants for potentially improved catalytic efficiency using the best psicose biosensor described above. For this, a *sine qua non* condition is the expression of a functional DPEase that is able to convert D-fructose into D-psicose at 37°C during *E. coli* growth.

To demonstrate the whole-cell bioproduction of D-psicose from D-fructose, *E. coli* cells were transformed with the pSB1C3 plasmid harboring the DPEase under the control of the pTacI promoter. The optimal concentration of substrate was investigated using concentrations of D-fructose ranging from 2 g/l to 300 g/l. A decrease in the growth of the culture could be noticed above 100 g/l of D-fructose, which might be due to osmotic stress on the cells. A maximal production of 9 g/l of D-psicose was reached after 24 h, using a D-fructose concentration of 50 g/l, which represents a yield of 18%.

This conversion rate is comparable to the biocatalysis yield described in the literature for this enzyme which retains at 37°C only 60% of its maximum activity that it has at 55°C (31). Higher biocatalysis yields have been reached, for example 23% at 70°C when using purified DPEase from *Dorea* sp. CAG317 (44), 31% at 65°C when permeabilizing the membrane of cells (45) or even 70% at 45°C with a mutated DPEase from *A. tumefaciens* immobilized on a surface (46).

Many aspects of the bioconversion could, therefore, be improved, using for instance higher temperatures to harness the optimal activity of DPEase, by permeabilizing the cells or even working on a cell-free method (47). However, the production conditions should match our screening process, which needs living cells. In the current settings, the primary bioconversion improvement will come from the selection of enhanced DPEase. These enzyme candidates could then be used in any D-psicose bioproduction process.

### 3.3 A screening method for gain of function mutants of *C. cellulolyticum* DPEase

Enzyme engineering currently focuses on computation modeling followed by directed mutagenesis on specific amino acids of the protein to improve its characteristics. This maximizes the probability of improving activity for a defined number of mutants but restricts possible random conformational changes, with the potential to improve catalytic sites. Conversely, random mutagenesis favors completely new conformations but requires screening a much larger number of mutants, hence the need to use an efficient screening system. For this purpose, we first engineered the biosensor to allow the insertion of mutants into the vector, in order to build the mutant library, and finally screened all the mutants for potentially improved catalytic efficiency.

**Figure 3.**
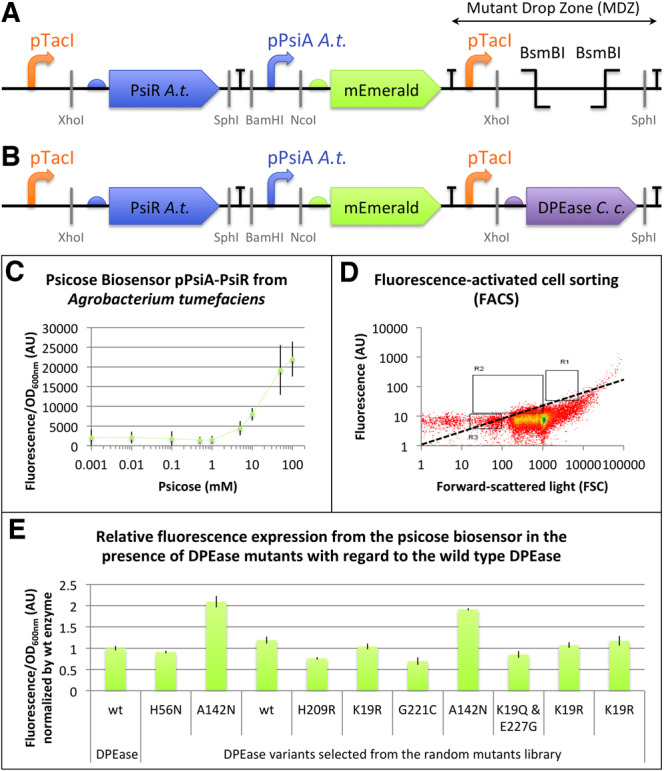
DPEase mutant library screening. (**A**) Schematic representation of the psicose biosensor based on pPsiA promoter from *A. tumefaciens* and the PsiR transcription factor from *A. tumefaciens* with downstream the MDZ (BBa_K2448057). (**B**) The DPEase from *C. cellulolyticum* or a DPEase mutant library generated by error-prone PCR were inserted in the MDZ of (A) by Golden Gate cloning using the BsmBI restriction endonuclease (BBa_K2448058). (**C**) *In vivo* characterization of mEmerald expression as reporter gene of the psicose biosensor represented schematically in (A). Fluorescence values (background subtracted) were normalized by OD_600nm_. The data and error bars are the mean and standard deviation of six measurements (three biological replicates, each measured as two technical duplicates). (**D**) FACS of *E. coli* cells harboring the psicose biosensor with a downstream DPEase library represented schematically in (B). Cells having the fluorescence/size ratio above average (dotted line) were isolated (regions R1, R2, R3). (**E**) *In vivo* characterization of mEmerald expression by *E. coli* cells harboring the psicose biosensor and ten DPEase mutants represented schematically in (B). All the data points are fluorescence values (background subtracted) normalized by OD_600nm_ of each mutant normalized by the same value from the control (wild-type DPEase). The data and error bars are the mean and standard deviation of three measurements.

The engineering of the biosensor consisted of adding, downstream of the reporter gene, a sequence that we refer to as the MDZ (Figure 3A). MDZ comprise the pTacI promoter followed by restriction sites that allow insertion of the DPEase in the same plasmid as the biosensor. To build the mutant DPEase library, we chose to use error-prone PCR because it favors mutations during the elongation phase, thanks to a mutagenic buffer (for example imbalance in dNTPs concentrations) and low fidelity polymerases. This technique remains more efficient than chemical methods, which rely on reagents to modify the sequence, and is safer for the user, as chemical mutagens are highly toxic. Moreover, it is an *a priori* free method compared to saturating mutagenesis. The protocol described by Wilson and Keefe (35) was applied on the full length coding sequence of *C. cellulolyticum* DPEase to build the library. According to this protocol, variants were obtained with a theoretical mutation average of eight amino acids. A high-fidelity PCR was performed on the same gBlocks with the same primers in order to obtain a non-mutated enzyme, as a positive control. Library sequences were inserted by Golden Gate assembly in the MDZ downstream of the psicose biosensor based on pPsiA promoter and the PsiR transcription factor from *A. tumefaciens* (Figure 3B) and the Golden Gate assembly products were transformed into *E. coli*. Due to technical constraints related to the cell sorter characteristics, the reporter gene mCherry was replaced by a less efficient mEmerald (37). This resulted in a decrease from 77-fold to a 16.8-fold change in fluorescence between 0 mM and 100 mM of D-psicose (Supplementary Figure S3). However, mEmerald shares common characteristics with mCherry relevant to the framework, such as rapid maturation and photostability, and proved to be sufficient to distinguish potentially improved mutants during screening.

In order to assess the DPEase enzyme activity, all the screening process was conducted on *E. coli* cells cultured in the presence of 50 g/l of fructose for 9–10 h before measurement, as this is the optimal measurement time according to our biosensor characterization. Then, FACS was used on a liquid culture of transformed cells (Figure 3D) to isolate mutants displaying superior catalytic efficiency compared to the wild-type DPEase enzyme. Cells having the fluorescence/size ratio above average (dotted line) were isolated (regions R1, R2 and R3) and subsequently spread on LB agar plates containing 35 µg/ml chloramphenicol. A total of 848 colonies were isolated between R1, R2 and R3 during this procedure.

In the next step, we chose 10 mutants to more precisely evaluate the psicose production using the biosensor. The fluorescence values of the cells producing psicose as well as the OD_600nm_ were measured in a plate reader 10 h after culturing in LB media supplemented with 80 g/l D-fructose. Figure 3E shows the relative fluorescence expression of the mutants with regard to the wild-type DPEase. Not surprisingly, the gain of function mutations are less likely to happen than loss of function and neutral mutations. Nonetheless, using FACS and then the plate reader characterization of 10 mutants we found 6 DPEase variants displaying various degrees of improvement in psicose production. We chose the mutant with the highest ratio of fluorescence/OD_600nm_ compared to the wild-type enzyme (*t*-test *P*-value <0.01) to further characterize it using purified DPEase.

Sequence analysis of the selected DPEase mutant revealed the presence of two mutations: a synonymous mutation of the codon of the serine residue in position 110 (TCT to TCA) and a non-synonymous mutation leading to alanine to asparagine substitution in position 142 (GCT to GAT). To further characterize this mutant, the DPEase sequence was extracted by PCR and placed under the control of the pTacI promoter. During this process, a Histidine Tag (identical to the one used in the literature for this DPEase (31)) was added at the C-terminus to allow rapid purification of the protein by Ni affinity chromatography. After protein overexpression in *E. coli* BL21-AI and purification, the kinetic parameters for the conversion of D-fructose to D-psicose were determined for the *C. cellulolyticum* DPEase (Supplementary Figure S4). The A142N mutant displayed a higher K_m_ for D-fructose (164 mM versus 77 mM for the wild-type enzyme) and a higher turnover number (8613 min^−1^ versus 3515 min^−1^ for the wild-type enzyme). A142 is a residue located at the end of an α-helix that is followed by a small loop and the β-strand bearing the catalytic glutamate (E150) (Supplementary Figure S5). This proximity may explain the differences in the kinetic parameters of the A142N mutant. An increased k_cat_ value is an interesting feature for an enzyme as it allows to speed up the conversion rate of the substrate into product, in our case D-fructose to D-psicose and it can be very useful in continuous psicose production methods like for instance those that use enzymes immobilized on a surface. For an *in vivo* production experiment in batch cultures of *E. coli*, this feature may have very limited effect, as the bioconversion of D-fructose to D-psicose reaches an equilibrium that depends on temperature and standard Gibbs free energy (48). Indeed, using the mutated enzyme in *E. coli* the production of D-psicose from was D-fructose (at an initial concentration of 50 g/l) was not significantly different from the wild-type histidine-tagged DPEase (8.96 ± 0.61 g/l for the A142N mutant versus 8.72 ± 0.11 g/l for the wild-type enzyme, the *t*-test *P*-value = 0.3572). These production values were reached after 24 h, and increasing the time of *E. coli* culturing to 48 h did not improved them. This result may seem in contradiction with the fluorescence measurements (Figure 3E) and with the cell sorting experiment (Figure 3D). However, even if the output in batch cultures is the same, the screening method based on genetic circuits allows detecting fine differences of enzymes behaviors highlighting the power or this approach. Indeed, this genetic circuit composed of a transducer (here in its simplest form, an enzyme transforming an undetectable substrate into a detectable product) and an actuator (the module capable of detecting a signal, here the PsiR transcription factor) acts in a manner that depends on substrate concentration, enzyme efficiency and cellular resource allocation (49).

## 4. Conclusions

In this work, we developed multiple biosensors for a high-value rare sugar, psicose, and screened its improved bioproduction using random mutagenesis. Recent advances in synthetic biology enable efficient implementation of design–build–test (DBT) cycle to develop new devices for industrial, medical and environmental applications. In this direction, biosensors are promising tools to equip metabolic and enzyme engineering with a monitoring ability. In this study, we show a workflow to DBT unconventional biosensors sensing new chemicals rather than those with well-known characterization. To do so, we provide the UBC to utilize the state of the art of characterized genetic parts as well as uncharacterized genes and promoters. The UBC architecture enables faster ‘design’ and ‘build’ of the biosensors which can be applied to a large number of transcription factors responding to different small molecules (50). Due to the ability of the quick characterization and prototyping using the biosensors, the ‘test’ phase of the DBT cycle can also be performed in a highly automated manner. Therefore, using this workflow, and taking advantage of the characterized genetic parts, an engineering DBT cycle brings sophisticated biosensors to pathway and enzyme engineers. Synthetic biosensors not only speed up the prototyping of the existing enzymes and pathways, but also provide the ability for monitoring rational engineering of the enzymes and pathways to develop new phenotypes.

## Supplementary Material

ysz028_Supplementary_Data
