# Supplementary material for: Biosensor-based enzyme engineering approach applied to psicose biosynthesis
Source: Synth Biol (Oxf). 2019 Dec 2;4(1):ysz028. doi: 10.1093/synbio/ysz028 (PMC7445875; doi:10.1093/synbio/ysz028)
Supplement: ysz028_Supplementary_Data [file ysz028_supplementary_data.zip › Armetta et al - Supplementary information.pdf]

Supplementary information for:

Biosensor-based enzyme engineering approach applied to psicose biosynthesis

Jeremy Armetta[1,2,†], Rose Berthome[1,3,†], Antonin Cros[1,2], Celine Pophillat[1,4], Bruno Maria Colombo[1], Amir Pandi[5,6] and Ioana Grigoras[1*]
[1] iSSB, UMR8030 Génomique Métabolique, Genoscope, Institut François Jacob, CEA, CNRS, Univ Evry, Université Paris-Saclay, Genopole Campus 1, Bât. 6, 5 rue Henri Desbruères, 91030 Evry, France.
[5] Micalis Institute, INRA, AgroParisTech, Université Paris-Saclay, 78350 Jouy-en-Josas, France.

*Corresponding author:
Ioana Grigoras
ioana.popescu@univ-evry.fr

[†]Jeremy Armetta and Rose Berthome contributed equally to this work

Present addresses:
[2] Novo Nordisk Foundation Center for Biosustainability, Technical University of Denmark, Kemitovert 220, 2800 Kongens Lyngby, Denmark.
[3] Ginkgo Bioworks Inc., 27 Drydock Avenue, Boston, MA 02210, United States.
[4] Cordeliers Research Center, UMRS INSERM 1138, 15 rue de l'École de Médecine, 75006 Paris, France
[6] Max Planck Institute for Terrestrial Microbiology, Department of Biochemistry and Synthetic Metabolism, Karl-von-Frisch-Strasse 10, Marburg, 35043, Germany.

Table of contents **Page**

Supplementary Figure S1. The gene clusters in *A. tumefaciens*, *S. fredii* and *S. meliloti* predicted to be involved in D-psicose utilization. 2

Supplementary Figure S2. Fluorescence/OD_{600nm} fold changes of the seven psicose biosensors. 3

Supplementary Figure S3. Fluorescence/OD_{600nm} fold changes of the psicose biosensor based on pPsiA promoter from *Agrobacterium tumefaciens* and the PsiR transcription factor from *Agrobacterium tumefaciens* with mCherry or mEmerald as reporter gene. 4

Supplementary Figure S4. Purification and kinetic characterization of *C. cellulolyticum* DPEase. 5

Supplementary Figure S5. Structure of *C. cellulolyticum* DPEase. 6

Supplementary Table S1. Sequences used in this study. 7

Supplementary Materials and Methods 34

Supplementary References 41

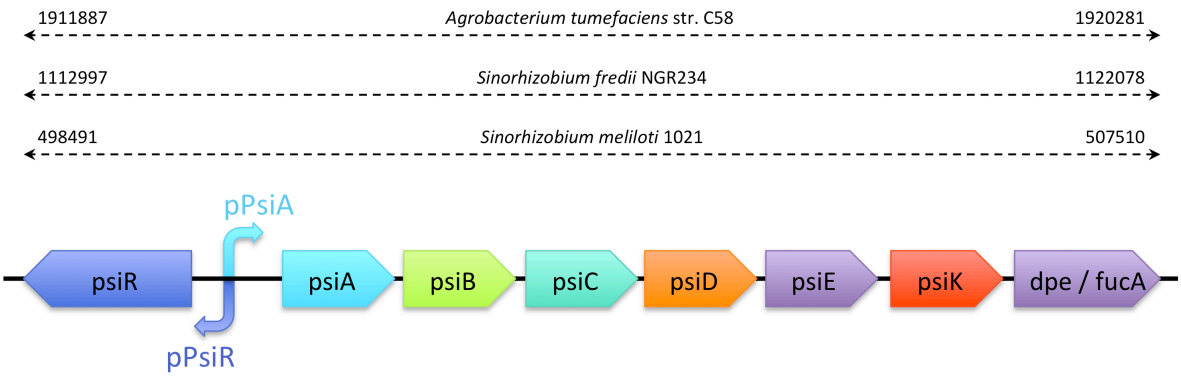

Gene	*A. tumefaciens*	*S. fredii*	*S. meliloti*	
psiR	Atu4743	NGR_b11520	SMb20483	Predicted transcriptional regulator for psicose utilization, LacI family
psiA	Atu4744	NGR_b11530	SMb20484	Predicted psicose ABC transporter, substrate-binding protein
psiB	Atu4745	NGR_b11540	SMb20485	Predicted psicose ABC transporter, ATP-binding protein
psiC	Atu4746	NGR_b11550	SMb20486	Predicted psicose ABC transporter, inner membrane protein
psiD	Atu4747	NGR_b11560	SMb20487	Predicted psicose ABC transporter, inner membrane protein
psiE	Atu4748	NGR_b11570	SMb20488	Fructose:psicose-3-epimerase (EC 5.3.1.-)
psiK / frcK	Atu4749	NGR_b11580	SMb20489	Fructokinase
dpe / fucA	Atu4750	NGR_b11590	SMb20490	D-psicose 3-epimerase / Ribulose-5-phosphate 4-epimerase and related epimerases and aldolases

Supplementary Figure S1. The gene clusters in *A. tumefaciens*, *S. fredii* and *S. meliloti* predicted to be involved in D-psicose utilization. The genome location of the clusters is indicated according to the NCBI reference sequences NC_003063.2, NC_012586.1 et NC_003078.1 respectively. For each of the 8 genes, the locus tag and the function of the encoded proteins is compiled. The two divergently oriented putative D-psicose inducible promoters are depicted.

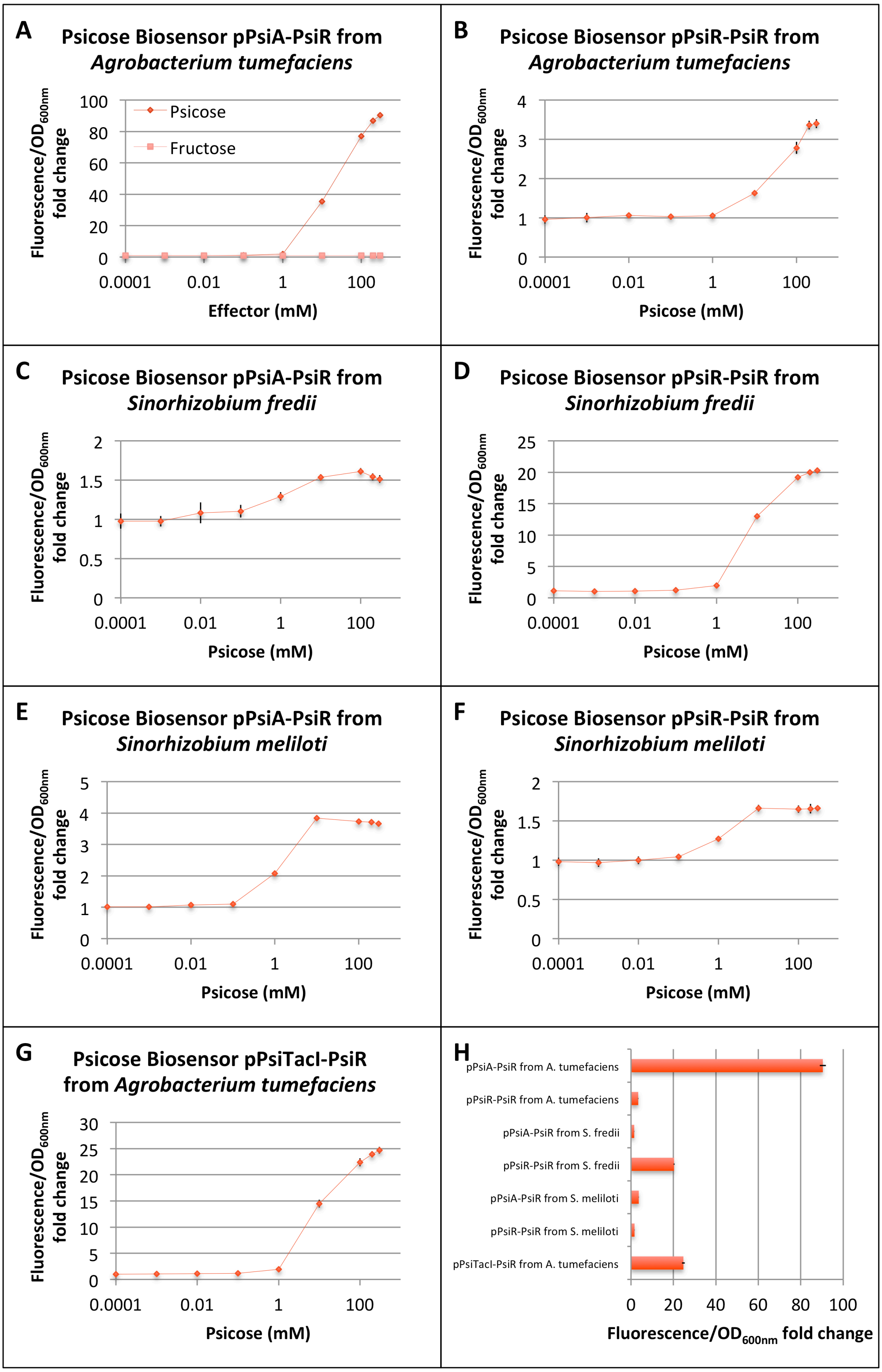

Supplementary Figure S2. Fluorescence/OD_{600nm} fold changes of the seven psicose biosensors. The fluorescence values (background subtracted) were normalized by OD_{600nm} (Figures 1 and 2) and represented (A-G) as fold change compared to the Fluorescence/OD_{600nm} value at 0 mM D-psicose. (H) The maximum fold change of each biosensor (the ratio of the fluorescence values when 300 or 0 mM of D-psicose are added). The data and error bars are the mean and standard deviation of six measurements (three biological replicates each measured as two technical duplicates).

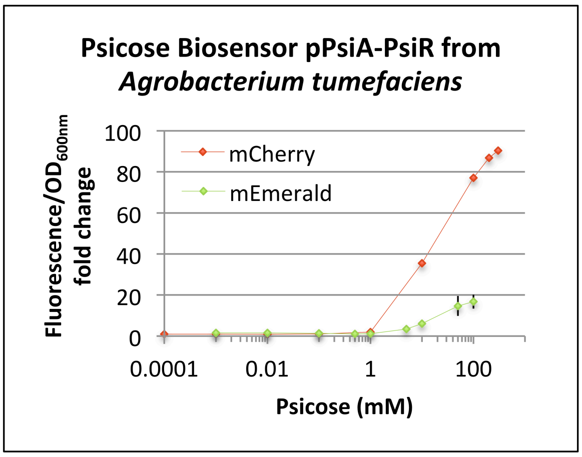

Supplementary Figure S3. Fluorescence/OD_{600nm} fold changes of the psicose biosensor based on pPsiA promoter from *Agrobacterium tumefaciens* and the PsiR transcription factor from *Agrobacterium tumefaciens* with mCherry or mEmerald as reporter gene. The fluorescence values (background subtracted) were normalized by OD_{600nm} (Figures 1A and 3C) and represented as fold change compared to the Fluorescence/OD_{600nm} value at 0 mM D-psicose. The data and error bars are the mean and standard deviation of six measurements (three biological replicates each measured as two technical duplicates) for mCherry and of three measurements in case of mEmerald.

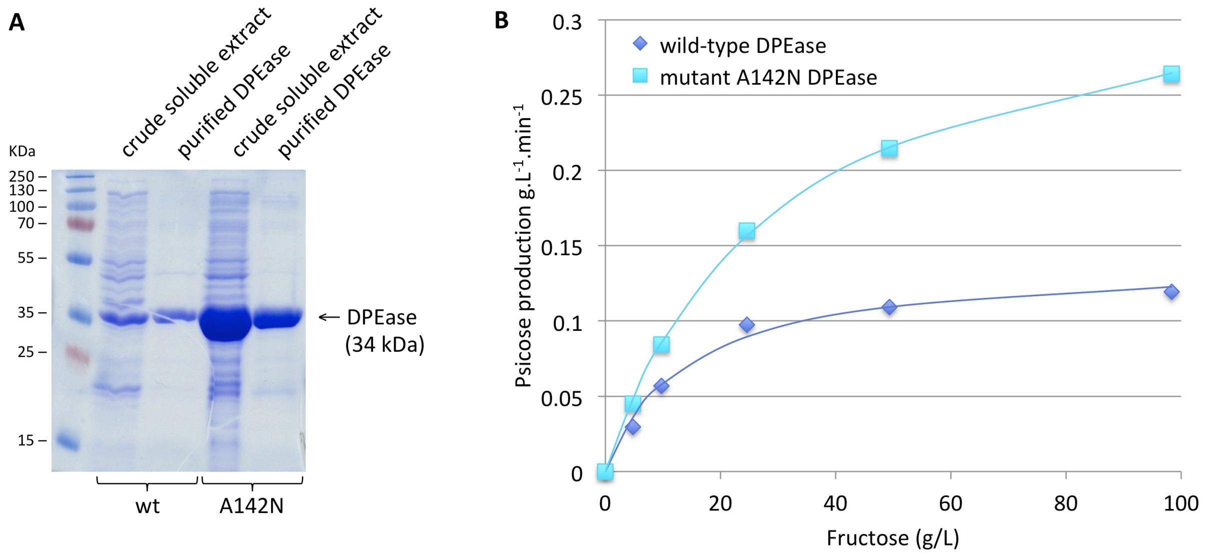

C

	Wild-type (1)	Wild-type	A142N
K_m (mM)	53.5 ± 1.8	77	164
k_{cat} (min^{-1})	3354.5 ± 47.2	3516	8613
k_{cat}/K_m ($mM^{-1}.min^{-1}$)	62.7 ± 1.8	46	53

Supplementary Figure S4. Purification and kinetic characterization of *C. cellulolyticum* DPEase. (A) Coomassie blue-stained SDS-PAGE profiles of crude soluble extract preparations obtained from the *E. coli* cells transformed with the plasmids expressing the D-Psicose 3-epimerase (DPEase) from *Clostridium cellulolyticum* with a C-terminal Histidine tag under the control of pTacI promoter and of the purified preparations derived from the corresponding crude extracts. (B) Steady-state kinetic parameters for the conversion of D-fructose into D-psicose by wild-type and mutant A142N DPEase. The assays were carried out as described in Materials and Methods. (C) The kinetic parameters of wild-type and mutant A142N DPEase determined from the plots are presented along with the values reported in the literature for the wild-type enzyme (1).

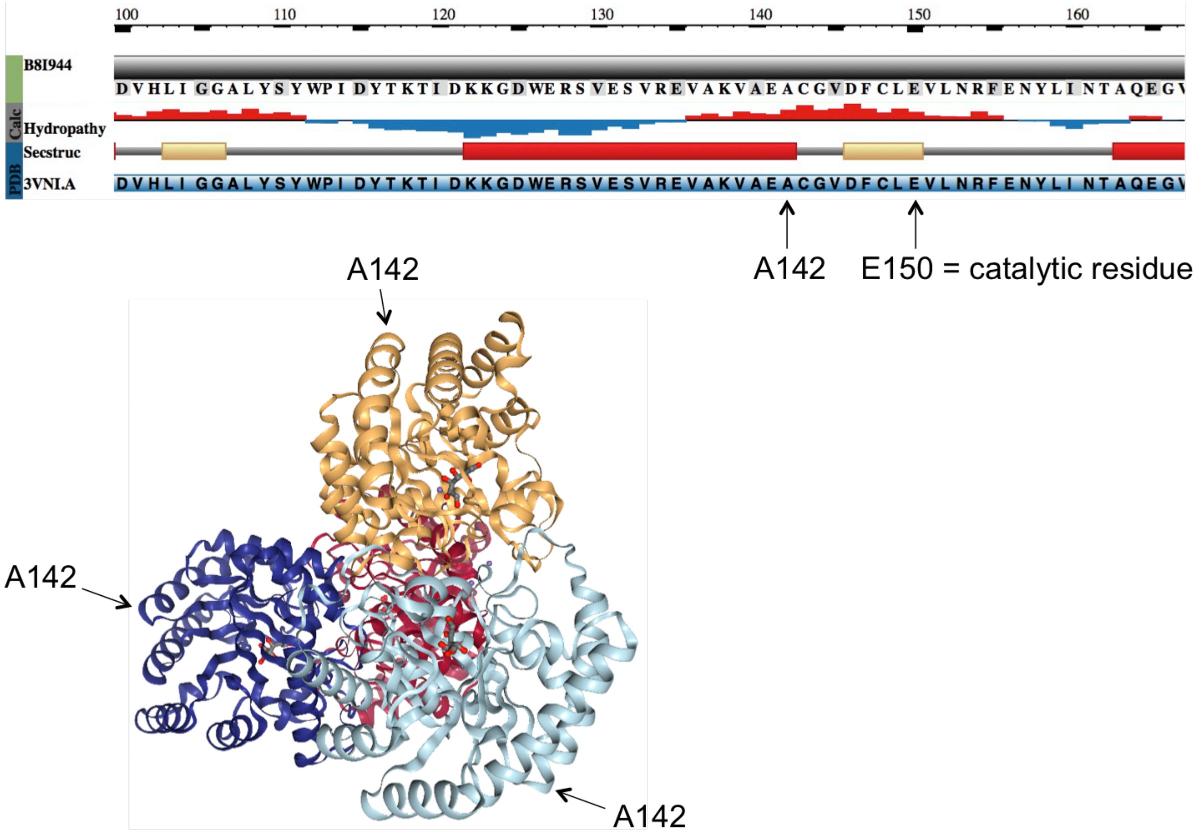

Supplementary Figure S5. Structure of *C. cellulolyticum* DPEase. (A) Protein feature view of PDB entry 3VNK mapped to the UniProt sequence B8I944. (B) Ribbon representation of PDB entry 3VNK (image created with NGL Viewer (2)). The catalytic glutamate (E150) is indicated as well as the alanine residue (A142) that was found mutated into an asparagine upon screening of a random mutant library for DPEase mutants for potentially improved catalytic efficiency.

Supplementary Table S1. Sequences used in this study.

Sequence name	**PsiR from *Agrobacterium tumefaciens***
Description	*E. coli* codon optimized version of the PsiR found in *Agrobacterium tumefaciens* str. C58 (gene Atu4743, UniProt A9CH24)
Acc. number	iGEM Parts Registry: BBa_K2448006<br>http://parts.igem.org/Part:BBa_K2448006
Sequence	ATGACCGGTATCTCTTCTAAAAAAGCTACCATCTACGACCTGTCTATCCTGT<br>CTGGTGCTTCTGCTTCTACCGTTTCTGCTGTTCTGAACGGTTCTTGGCGTAA<br>ACGTCGTATCTCTGAAGAAACCGCTGACAAAATCCTGTCTCTGGCTAAAGC<br>TCAGCGTTACACCACCAACTTACAGGCTCGTGGTCTGCGTTCTTCTAAATCT<br>GGTCTGGTTGGTCTGCTGGTTCCGGTTTACGACAACCGTTTCTTCTCTTCTA<br>TGGCTCAGACCTTCGAAGGTCAGGCTCGTAAACGTGGTCTGTCTCCGATGG<br>TTGTTTCTGGTCGTCGTGACCCGGAAGAAGAACGTCGTACCGTTGAAACCC<br>TGATCGCTTACTCTATCGACGCTCTGTTCATCGCTGGTGTTACCGACCCGG<br>ACGGTGTTCACCAGGTTTGCGCTCGTGCTGCTCTGCCGCACGTTAACATCG<br>ACCTGCCGGGTAAATTCGCTTCTTCTGTTATCTCTAACAACCGTCACGGTGC<br>TGAAATCCTGACCGCTGCTATCCTGGCTCACGCTGCTAAAGGTGGTTCTCT<br>GGGTCCGGACGACGTTATCCTGTTCGGTGGTCACGACGACCACGCTTCTC<br>GTGAACGTATCGACGGTTTCCACGCTGCTAAAGCTGACTACTTCGGTGTTG<br>AAGGTGGTGACGACATCGAAATCACCGGTTACTCTCCGCACATGACCGAAA<br>TGGCTTTCGAACGTTTCTTCGGTCGTCGTGGTCGTCTGCCGCGTTGCTTCT<br>TCGTTAACTCTTCTATCAACTTCGAAGGTCTGCTGCGTTTCATGGGTCGTCA<br>CGACGGTGAAGCTTTCGGTGACATCGTTGTTGGTTGCTTCGACTACGACCC<br>GTTCGCTTCTTTCCTGCCGTTCCCGGTTTACATGATCAAACCGGACATCGCT<br>CAGATGCTGGAAAAAGGTTTCGAACTGCTGGAAGAAAACCGTACCGAACCG<br>GAAGTTACCATCATCGAACCGCAGCTGATCCCGCCGCGTACCGCTCTGGA<br>AGGTCCGCTGGACGACATCTGGGACCCGGTTGCTCTGCGTCGTATGGCTA<br>AATAA

Sequence name	**PsiR from *Sinorhizobium fredii***
Description	*E. coli* codon optimized version of the PsiR found in *Sinorhizobium fredii* (gene NGR_b11520, UniProt C3KR97)
Acc. number	iGEM Parts Registry: BBa_K2448007<br>http://parts.igem.org/Part:BBa_K2448007
Sequence	ATGGCTAACTCTGGTAAAAAAAAAGCTACCATCTACGACCTGTCTGTTCTGT<br>CTGGTTCTTCTCCGTCTACCGTTTCTGCTGTTCTGAACGGTACCTGGCGTAA<br>ACGTCGTATCAAAGAATCTACCGCTGAACTGATCCGTAACCTGGCTGAAAC<br>CCACCAGTACACCGCTAACCGTCAGGCTCGTGGTCTGCGTTCTTCTCGTTC<br>TGGTCTGGTTGGTCTGCTGCTGCCGGTTCACGACAACCGTTACTTCTCTTC<br>TCTGGCTCAGACCTTCGAAGCTCACGTTCGTTCTAAAGGTCAGTGCCCGAT<br>CGTTGTTTCTGCTTCTCGTGACCCGCAGGAAGAACGTAAAACCGCTGAAAC<br>CCTGATCTCTTACTCTATCGACGAACTGTTCATCTGCGGTGCTACCGACCC<br>GGACGGTGTTCACGAAGTTTGCGAAGCTGCTGGTCTGAAACACATCAACAT<br>CGACCTGCCGGGTACCAAAGTTCCGTCTGTTATCTCTGACAACTTCGAAGG<br>TGGTCGTCTGCTGACCGAAGCTATCATCCGTCACTTCCCGGCTGACCGTGC<br>TCTGGCTCCGACCGACCTGTACCTGTTCGGTGGTCGTAACGACCACGCTTC<br>TCACGAACGTATCCGTGGTTTCCGTGCTGTTAAAAAAGACCTGCTGGGTGA<br>CGACCCGGACGAATGCATCCAGCCGACCGGTTACGCTGCTAACAACGCTC<br>GTAAAGCGTTCGAAGCGTTCTACGCTCGTCACGGTAAACTGCCGCGTGGTC

	TGTTCGTTAACTCTTCTATCAACTTCGAAGGTCTGCTGCGTTTCATGGCTGA ACACCCGCACGACAACTTCACCGACCTGGTTGTTGGTTGCTACGACTACGA CCCGTTCGCTTCTTTCCTGCCGTTCCCGGTTATCATGATCCGTCAGGACGT TGAAGGTATGATCGCTAAAGCGTTCGAAGTTATCGAACAGCCGCGTGCTCT GGCTCGTATCCACCTGGTTCAGCCGGAACTGGTTCCGCCGCGTACCGCTC TGACCGGTCCGCTGGACGCTCTGAAAGACATCGACCTGCCGCGTGGTTCT CAGTAA

Sequence name	**PsiR from *Sinorhizobium meliloti***
Description	*E. coli* codon optimized version of the PsiR found in *Sinorhizobium meliloti* (gene SMb20483, UniProt Q92W80)
Acc. number	iGEM Parts Registry: BBa_K2448008 http://parts.igem.org/Part:BBa_K2448008
Sequence	ATGACCAACGGTGGTCGTAAAAAAGCTACCATCTACGACCTGTCTGTTCTG TCTGGTTCTTCTCCGTCTACCGTTTCTGCTGTTCTGAACGGTACCTGGCGTA AACGTCGTATCAAAGAATCTACCGCTGAACTGATCCGTTCTCTGGCTGAAA CCCACCAGTACACCGCTAACCGTCAGGCTCGTGGTCTGCGTTCTTCTCGTT CTGGTCTGGTTGGTCTGCTGCTGCCGGTTCACGACAACCGTTACTTCTCTT CTCTGGCTCAGACCTTCGAAGCTCACGTTCGTTCTAAAGGTCAGTGCCCGA TCGTTGTTTCTGCTTCTCGTGACCCGGAAGAAGAACGTCGTACCGCTGAAA CCCTGATCTCTTACTCTATCGACGAACTGTTCATCTGCGGTGCTACCGACC CGGACGGTGTTCACGAAGTTTGCGAAGCTGCTGGTCTGCGTCACATCAACA TCGACCTGCCGGGTACCAAAGTTCCGTCTGTTATCTCTGACAACTTCGAAG GTGGTCGTCTGCTGACCGAAGCTATCATCCGTCACTTCCCGGCTGAACGTC CGCTGGAACCGGACGACCTGTACCTGTTCGGTGGTCGTGACGACCACGCT ACCCGTGAACGTATCCGTGGTTTCCGTGCTGTTAAATCTGACCTGCTGGGT GCTGACCCGGACGAATGCATCTGGCCGACCGGTTACGCTGCTGACAACGC TCGTAAAGCGTTCGAAGCGTTCTACGAACAGCACGGTAAACTGCCGCGTG GTTTCTTCGTTAACTCTTCTATCAACTTCGAAGGTCTGCTGCGTTTCATGGC TGAACACCCGCTGGAAAACTTCACCGACCTGGTTGTTGGTTGCTACGACTA CGACCCGTTCGCTTCTTTCCTGCCGTTCCCGGTTATCATGATCCGTCAGAA CATCGAAGGTATGATCGCTAAAGCGTTCGAAGTTATCGAAGAACCGCGTGC TTCTCTGCAAATCCACATGATCGAACCGCAGCTGGTTCCGCCGCGTACCGC TCTGACCGGTCCGCTGGACGCTCTGATGGACTCTGAAATGCCGCGTGAATA A

Sequence name	**D-Psicose 3-epimerase (DPEase) from *Clostridium cellulolyticum***
Description	*E. coli* codon optimized version of the D-psicose 3-epimerase (DPEase) from *Clostridium cellulolyticum* str. ATCC 35319 (gene Ccel_0941, UniProt B8I944)
Acc. number	iGEM Parts Registry: BBa_K2448021 http://parts.igem.org/Part:BBa_K2448021
Sequence	ATGAAACACGGTATCTACTACGCTTACTGGGAACAGGAATGGGAAGCTGAC TACAAATACTACATCGAAAAAGTTGCTAAACTGGGTTTCGACATCCTGGAAA TCGCTGCTTCTCCGCTGCCGTTCTACTCTGACATCCAGATCAACGAACTGA AAGCTTGCGCTCACGGTAACGGTATCACCCTGACCGTTGGTCACGGTCCGT CTGCTGAACAGAACCTGTCTTCTCCGGACCCGGACATCCGTAAAAACGCTA AAGCTTTCTACACCGACCTGCTGAAACGTCTGTACAAACTGGACGTTCACC TGATCGGTGGTGCTCTGTACTCTTACTGGCCGATCGACTACACCAAAACCA TCGACAAAAAAGGTGACTGGGAACGTTCTGTTGAATCTGTTCGTGAAGTTG

	CTAAAGTTGCTGAAGCTTGCGGTGTTGACTTCTGCCTGGAAGTTCTGAACC<br>GTTTCGAAAACTACCTGATCAACACCGCTCAGGAAGGTGTTGACTTCGTTAA<br>ACAGGTTGACCACAACAACGTTAAAGTTATGCTGGACACCTTCCACATGAA<br>CATCGAAGAAGACTCTATCGGTGGTGCTATCCGTACCGCTGGTTCTTACCT<br>GGGTCACCTGCACACCGGTGAATGCAACCGTAAAGTTCCGGGTCGTGGTC<br>GTATCCCGTGGGTTGAAATCGGTGAAGCTCTGGCTGACATCGGTTACAACG<br>GTTCTGTTGTTATGGAACCGTTCGTTCGTATGGGTGGTACCGTTGGTTCTAA<br>CATCAAAGTTTGGCGTGACATCTCTAACGGTGCTGACGAAAAAATGCTGGA<br>CCGTGAAGCTCAGGCTGCTCTGGACTTCTCTCGTTACGTTCTGGAATGCCA<br>CAAACACTCTTAA

Sequence name	**D-Psicose 3-epimerase (DPEase) from *Clostridium cellulolyticum* with a C-terminal HisTag**
Description	*E. coli* codon optimized version of the D-psicose 3-epimerase (DPEase) from *Clostridium cellulolyticum* str. ATCC 35319 (gene Ccel_0941, UniProt B8I944) with a C-terminal HisTag
Acc. number	iGEM Parts Registry: BBa_K2448053<br>http://parts.igem.org/Part:BBa_K2448053
Sequence	ATGAAACACGGTATCTACTACGCTTACTGGGAACAGGAATGGGAAGCTGAC<br>TACAAATACTACATCGAAAAAGTTGCTAAACTGGGTTTCGACATCCTGGAAA<br>TCGCTGCTTCTCCGCTGCCGTTCTACTCTGACATCCAGATCAACGAACTGA<br>AAGCTTGCGCTCACGGTAACGGTATCACCCTGACCGTTGGTCACGGTCCGT<br>CTGCTGAACAGAACCTGTCTTCTCCGGACCCGGACATCCGTAAAAACGCTA<br>AAGCTTTCTACACCGACCTGCTGAAACGTCTGTACAAACTGGACGTTCACC<br>TGATCGGTGGTGCTCTGTACTCTTACTGGCCGATCGACTACACCAAAACCA<br>TCGACAAAAAAGGTGACTGGGAACGTTCTGTTGAATCTGTTCGTGAAGTTG<br>CTAAAGTTGCTGAAGCTTGCGGTGTTGACTTCTGCCTGGAAGTTCTGAACC<br>GTTTCGAAAACTACCTGATCAACACCGCTCAGGAAGGTGTTGACTTCGTTAA<br>ACAGGTTGACCACAACAACGTTAAAGTTATGCTGGACACCTTCCACATGAA<br>CATCGAAGAAGACTCTATCGGTGGTGCTATCCGTACCGCTGGTTCTTACCT<br>GGGTCACCTGCACACCGGTGAATGCAACCGTAAAGTTCCGGGTCGTGGTC<br>GTATCCCGTGGGTTGAAATCGGTGAAGCTCTGGCTGACATCGGTTACAACG<br>GTTCTGTTGTTATGGAACCGTTCGTTCGTATGGGTGGTACCGTTGGTTCTAA<br>CATCAAAGTTTGGCGTGACATCTCTAACGGTGCTGACGAAAAAATGCTGGA<br>CCGTGAAGCTCAGGCTGCTCTGGACTTCTCTCGTTACGTTCTGGAATGCCA<br>CAAACACTCTCTCGAGCACCACCATCACCACCACTAA

Sequence name	**mCherry**
Description	*E. coli* codon optimized version of the mCherry fluorescent protein (UniProt X5DSL3)
Acc. number	iGEM Parts Registry: BBa_K2448004<br>http://parts.igem.org/Part:BBa_K2448004
Sequence	ATGGTTTCTAAAGGTGAAGAAGATAACATGGCTATCATCAAAGAATTTATGC<br>GTTTCAAAGTTCACATGGAAGGTTCTGTTAACGGTCACGAATTTGAAATCGA<br>AGGTGAAGGTGAAGGTCGTCCGTACGAAGGTACCCAGACCGCTAAACTGA<br>AAGTTACCAAAGGTGGTCCGCTGCCGTTCGCTTGGGACATCCTGTCTCCGC<br>AGTTCATGTACGGTTCTAAAGCGTACGTTAAACACCCGGCTGACATCCCGG<br>ACTACCTGAAACTGTCTTTCCCGGAAGGTTTCAAATGGGAACGTGTTATGAA<br>CTTCGAAGATGGTGGTGTTGTTACCGTTACCCAGGACTCTTCTCTGCAAGA<br>CGGTGAATTTATCTACAAAGTTAAACTGCGTGGTACCAACTTCCCGTCTGAC

	GGTCCGGTTATGCAGAAAAAAACTATGGGTTGGGAAGCGAGCTCTGAACGT<br>ATGTACCCGGAAGATGGTGCTCTGAAAGGTGAAATCAAACAGCGTCTGAAA<br>CTGAAAGACGGTGGTCACTACGACGCTGAAGTTAAAACCACCTACAAAGCT<br>AAAAAACCGGTTCAGCTGCCGGGTGCTTACAACGTTAACATCAAACTGGAC<br>ATCACCTCTCACAACGAAGATTACACCATCGTTGAACAGTACGAACGTGCT<br>GAAGGTCGTCACTCTACCGGTGGTATGGACGAACTGTACAAATAA

Sequence name	**mEmerald**
Description	*E. coli* codon optimized version of the mEmerald fluorescent protein (FPbase AD4BK)
Acc. number	iGEM Parts Registry: BBa_K2448001<br>http://parts.igem.org/Part:BBa_K2448001
Sequence	ATGGTTTCTAAAGGTGAAGAACTGTTCACCGGTGTTGTTCCGATCCTGGTT<br>GAACTGGACGGTGACGTTAACGGTCACAAATTCTCTGTTTCTGGTGAAGGT<br>GAAGGTGACGCTACCTACGGTAAACTGACCCTGAAATTCATCTGCACCACC<br>GGTAAACTGCCGGTTCCGTGGCCGACCCTGGTTACCACCCTGACCTACGG<br>TGTTCAGTGCTTCGCTCGTTACCCGGACCACATGAAACAGCACGACTTCTT<br>CAAATCTGCTATGCCGGAAGGTTACGTTCAGGAACGTACCATCTTCTTCAAA<br>GACGACGGTAACTACAAAACCCGTGCTGAAGTTAAATTCGAAGGTGACACC<br>CTGGTTAACCGTATCGAACTGAAAGGTATCGACTTCAAAGAAGATGGTAAC<br>ATCCTGGGTCACAAACTGGAATACAACTACAACTCTCACAAAGTTTACATCA<br>CCGCTGACAAACAGAAAAACGGTATCAAAGTTAACTTCAAAACCCGTCACAA<br>CATCGAAGATGGTTCTGTTCAGCTGGCTGACCACTACCAGCAGAACACCCC<br>GATCGGTGACGGTCCGGTTCTGCTGCCGGACAACCACTACCTGTCTACCC<br>AGTCTAAACTGTCTAAAGACCCGAACGAAAAACGTGACCACATGGTTCTGC<br>TGGAATTTGTTACCGCTGCTGGTATCACCCTGGGTATGGACGAACTGTACA<br>AATAA

Sequence name	**LacZ-alpha**
Description	alpha fragment of the LacZ gene derived from the pUC19 cloning vector
Acc. number	iGEM Parts Registry: BBa_K2448003<br>http://parts.igem.org/Part:BBa_K2448003
Sequence	ATGACCATGATCACCCCGAGCCTGCACGCTTGCCGTTCTACCCTGGAAGAT<br>CCGCGTGTTCCGAGCTCTAACTCTCTGGCTGTTGTTCTGCAACGTCGTGAC<br>TGGGAAAACCCGGGTGTTACCCAGCTGAACCGTCTGGCTGCTCACCCGCC<br>GTTCGCTTCTTGGCGTAACTCTGAAGAAGCTCGTACCGACCGTCCGAGCCA<br>GCAGCTGCGTTCTCTGAACGGTGAATGGCGTCTGATGCGTTACTTCCTGCT<br>GACCCACCTGTGCGGTATCTCTCACCGTATCTGGTGCACCCTGTCTACCAT<br>CTGCTCTGACGCTGCTTAA

Sequence name	**pPsiA from *Agrobacterium tumefaciens***
Description	the promoter region (0.4 kb upstream) of the PsiA gene of *Agrobacterium tumefaciens* str. C58 (gene Atu4744) corresponding to nucleotides 1912931 to 1913328 of *Agrobacterium tumefaciens* str. C58 (GenBank AE007870.2)
Acc. number	iGEM Parts Registry: BBa_K2448010

	http://parts.igem.org/Part:BBa_K2448010
Sequence	GTATAAATGGTGGCTTTTTTTGAACTTATGCCCGTCACTGTGATCTCCCCAA<br>CTGATTCCGATTATTAGAGCACGCATCCCCTTGACGGAAGGGCGCTTCATG<br>ATATGGTTATTGCACCATCGATTGTGCAGATTGGCAATATCGATTGTGCATG<br>GTGGTTGCTATGGGAGTGGCAAGGGAGAGTCTCGAATAAGCGAGATGAGA<br>GATTTTGAACGCGTCCGGGAAAAACGGGCTGCGGGCGGATTTCGTTTGCC<br>GAATTTTTGAGGAGGAACATCAATGAAGAAAATTATTGCTGCGGCGGTTGG<br>TCTGTCGCTGGCGTTGCTCTCATCCGCAGCCTTTGCCGAAGGGCCGAAGG<br>TGGGCGTCGTCGTCAAGATCGGCGGCATTCCGTGGTTCAACGCC

Sequence name	**pPsiR from *Agrobacterium tumefaciens***
Description	the promoter region (0.36 kb upstream) of the PsiR gene of *Agrobacterium tumefaciens* str. C58 (gene Atu4743) corresponding to nucleotides 1912967 to 1913328 of *Agrobacterium tumefaciens* str. C58 (GenBank AE007870.2)
Acc. number	iGEM Parts Registry: BBa_K2448011<br>http://parts.igem.org/Part:BBa_K2448011
Sequence	GGAGGCGTTGAACCACGGAATGCCGCCGATCTTGACGACGACGCCCACCT<br>TCGGCCCTTCGGCAAAGGCTGCGGATGAGAGCAACGCCAGCGACAGACCA<br>ACCGCCGCAGCAATAATTTTCTTCATTGATGTTCCTCCTCAAAAATTCGGCA<br>AACGAAATCCGCCCGCAGCCCGTTTTTCCCGGACGCGTTCAAAATCTCTCA<br>TCTCGCTTATTCGAGACTCTCCCTTGCCACTCCCATAGCAACCACCATGCA<br>CAATCGATATTGCCAATCTGCACAATCGATGGTGCAATAACCATATCATGAA<br>GCGCCCTTCCGTCAAGGGGATGCGTGCTCTAATAATCGGAATCAGTTGGG<br>GAGATCACAT

Sequence name	**pPsiA from *Sinorhizobium fredii***
Description	the promoter region (0.4 kb upstream) of the PsiA gene of *Sinorhizobium fredii* (gene NGR_b11530) corresponding to nucleotides 1113914 to 1114313 of *Sinorhizobium fredii* NGR234 plasmid pNGR234b (GenBank CP000874.1) with 2 modifications to remove a BsaI site and a PstI site (to allow Golden Gate assembly use and comply to iGEM BioBrick RFC[10] standard)
Acc. number	iGEM Parts Registry: BBa_K2448012<br>http://parts.igem.org/Part:BBa_K2448012
Sequence	GGTGGGTCTGGGCGAGGTTGCGGATCAACTCGGCGGTGCTTTCCTTGATG<br>CGCCGCTTGCGCCAGGTGCCGTTCAGCACGGCGCTGACCGTCGAGGGCG<br>AGCTGCCGGAGAGCACCGAGAGATCGTAGATCGTCGCCTTTTTCTTGCCG<br>CTGTTCGCCATCCGAGCCCCCTCGAATCTCTTAGAGCCGTTTTGCGCTTGA<br>CGAAAGATTAAGTCTGCACGATAGTCTTTGCACCATCGATTGTGCAAATAAG<br>AAATATCGATTGTGCAGCTCTTTGGGCCGTCTGAGGAGGCGGCGGTCAGC<br>GGCGGGAAACGCGCTTCTCGTCATGGAGGATTGAAACTGGAGGCCGGCGC<br>GCCAGCGCCCGGGAGAGTTCCCGTTGCGGGAACCTGTGGAGGAGAGAC

Sequence name	**pPsiR from *Sinorhizobium fredii***
Description	the promoter region (0.4 kb upstream) of the PsiR gene of *Sinorhizobium fredii* (gene NGR_b11520) corresponding to nucleotides 1114473 to 1114074 of

	Sinorhizobium fredii NGR234 plasmid pNGR234b (GenBank CP000874.1) with 2 modifications to remove a NcoI site and a PstI site (to allow Golden Gate assembly use and comply to iGEM BioBrick RFC[10] standard)
Acc. number	iGEM Parts Registry: BBa_K2448013<br>http://parts.igem.org/Part:BBa_K2448013
Sequence	GGCCGCGCTCCTTGATGCCGACTTGCATGGCGTTGAACCACGGAATGCCG<br>CCGATCTTGACGACCACGCCGACCTTCGGCGCATCCTGCGCCGCGACGGA<br>AAAGGCACCGGCGAGCGAAAGCGAAGCCGCCAGAGCGGCAGCAAGAAAT<br>GTCTTGATCATGTCTCTCCTCCACAGGTTCCCGCAACGGGAACTCTCCCGG<br>GCGCTGGCGCGCCGGCCTCCAGTTTCAATCCTCCATGACGAGAAGCGCGT<br>TTCCCGCCGCTGACCGCCGCCTCCTCAGACGGCCCAAAGAGCTGCACAAT<br>CGATATTTCTTATTTGCACAATCGATGGTGCAAAGACTATCCTGCTGACTTA<br>ATCTTTCGTCAAGCGCAAAACGGCTCTAAGAGATTCGAGGGGGCTCGG

Sequence name	**pPsiA from *Sinorhizobium meliloti***
Description	the promoter region (0.4 kb upstream) of the PsiA gene of *Sinorhizobium meliloti* (gene SMb20484) corresponding to nucleotides 499435 to 499834 of *Sinorhizobium meliloti* 1021 plasmid pSymB (GenBank AL591985.1)
Acc. number	iGEM Parts Registry: BBa_K2448014<br>http://parts.igem.org/Part:BBa_K2448014
Sequence	CGGTGCTTTCCTTGATCCGTCGCTTGCGCCACGTGCCGTTTAGCACCGCAC<br>TGACGGTAGAGGGCGAACTTCCCGACAGCACCGAGAGATCATAGATCGTC<br>GCTTTTTTCCTGCCGCCGTTCGTCATCTGACCTCCTCCAAACCCCGGAAAA<br>CCGATGCGCACGTTTCCTGGAATTGCTCTAGTGCCGATTTCGGCTTGACGA<br>AAGATTAAGTCTGAATGATAGTCATTGCACCATCGATTGTGCAAAAAAGAAA<br>TATCGATTGTGCAAGTTGTTGGTGCCGTCTGAGGAGGCGGCCGTCAGCGG<br>CGGGATATCCCCTTCCGTGCAAAAGAATTAAGCTGGAGGCCGGCGCGTGA<br>AGCGCCCGGGAGCGTTCCCCTCGGGGAAACATGTGGAGGAGAAAC

Sequence name	**pPsiR from *Sinorhizobium meliloti***
Description	the promoter region (0.4 kb upstream) of the PsiR gene of *Sinorhizobium meliloti* (gene SMb20483) corresponding to nucleotides 499961 to 499562 of *Sinorhizobium meliloti* 1021 plasmid pSymB (GenBank AL591985.1)
Acc. number	iGEM Parts Registry: BBa_K2448015<br>http://parts.igem.org/Part:BBa_K2448015
Sequence	TGAACCACGGTATGCCGCCGATCTTGACGACGACACCGACCTTGCCCGTAT<br>CCTGCGCCGCGGCGGTATAGGCACCCGCAAGCGAAAGCGACGCCGCCAG<br>AGCGGCGGCAAGAATTTTCTTGATCATGTTTCTCCTCCACATGTTTCCCCGA<br>GGGGAACGCTCCCGGGCGCTTCACGCGCCGGCCTCCAGCTTAATTCTTTT<br>GCACGGAAGGGGATATCCCGCCGCTGACGGCCGCCTCCTCAGACGGCAC<br>CAACAACTTGCACAATCGATATTTCTTTTTTGCACAATCGATGGTGCAATGA<br>CTATCATTCAGACTTAATCTTTCGTCAAGCCGAAATCGGCACTAGAGCAATT<br>CCAGGAAACGTGCGCATCGGTTTTCCGGGGTTTGGAGGAGGTCAG

Sequence name	**pPsiTacI**
Description	a hybrid synthetic promoter composed of the -35 and the Pribnow box sequences of pTacI promoter and the consensus binding site of PsiR regulator of Rhizobiale
Acc. number	iGEM Parts Registry: BBa_K2448016<br>http://parts.igem.org/Part:BBa_K2448016
Sequence	TGAGCTGTTGACAATTAATCATCGGCTCGTATAATGTGTGGATTGCACAATC<br>GATGGTGCAA

Sequence name	**pTacI**
Description	a hybrid synthetic promoter derived from the *E. coli* trp and lac UV5 promoters
Acc. number	iGEM Parts Registry: BBa_K864400<br>http://parts.igem.org/Part:BBa_K864400
Sequence	GAGCTGTTGACAATTAATCATCGGCTCGTATAATGTGTGGAATTGTGAGCG<br>GATAACAATT

Sequence name	**J23100 promoter**
Description	a constitutive synthetic promoter isolated from a small combinatorial library
Acc. number	iGEM Parts Registry: BBa_J23100<br>http://parts.igem.org/Part:BBa_J23100
Sequence	TTGACGGCTAGCTCAGTCCTAGGTACAGTGCTAGC

Sequence name	**B0015 Terminator**
Description	double transcriptional terminator consisting of terminator T1 of the *Escherichia coli* rrnB gene and the terminator TE of coliphage T7 DNA ligase gene
Acc. number	iGEM Parts Registry: BBa_B0015<br>http://parts.igem.org/Part:BBa_B0015
Sequence	CCAGGCATCAAATAAAACGAAAGGCTCAGTCGAAAGACTGGGCCTTTCGTT<br>TTATCTGTTGTTTGTCGGTGAACGCTCTCTACTAGAGTCACACTGGCTCACC<br>TTCGGGTGGGCCTTTCTGCGTTTATA

Sequence name	**L2U3H03 Terminator**
Description	synthetic transcriptional terminator
Acc. number	iGEM Parts Registry: BBa_K2448059<br>http://parts.igem.org/Part:BBa_K2448059
Sequence	TAGCGTGACCGGCGCATCGGTCACGCTATTTGTTGAG

Sequence name	**Universal Biosensing Chassis (UBC)**
Acc. number	iGEM Parts Registry: BBa_K2448023<br>http://parts.igem.org/Part:BBa_K2448023
Sequence	TCCGGCAAAAAAGGGCAAGGTGTCACCACCCTGCCCTTTTTCTTTAAAACC<br>GAAAAGATTACTTCGCGTTATGCAGGCTTCCTCGCTCACTGACTCGCTGCG<br>CTCGGTCGTTCGGCTGCGGCGAGCGGTATCAGCTCACTCAAAGGCGGTAA<br>TACGGTTATCCACAGAATCAGGGGATAACGCAGGAAAGAACATGTGAGCAA<br>AAGGCCAGCAAAAGGCCAGGAACCGTAAAAAGGCCGCGTTGCTGGCGTTT<br>TTCCACAGGCTCCGCCCCCCTGACGAGCATCACAAAAATCGACGCTCAAGT<br>CAGAGGTGGCGAAACCCGACAGGACTATAAAGATACCAGGCGTTTCCCCC<br>TGGAAGCTCCCTCGTGCGCTCTCCTGTTCCGACCCTGCCGCTTACCGGATA<br>CCTGTCCGCCTTTCTCCCTTCGGGAAGCGTGGCGCTTTCTCATAGCTCACG<br>CTGTAGGTATCTCAGTTCGGTGTAGGTCGTTCGCTCCAAGCTGGGCTGTGT<br>GCACGAACCCCCCGTTCAGCCCGACCGCTGCGCCTTATCCGGTAACTATC<br>GTCTTGAGTCCAACCCGGTAAGACACGACTTATCGCCACTGGCAGCAGCCA<br>CTGGTAACAGGATTAGCAGAGCGAGGTATGTAGGCGGTGCTACAGAGTTCT<br>TGAAGTGGTGGCCTAACTACGGCTACACTAGAAGAACAGTATTTGGTATCT<br>GCGCTCTGCTGAAGCCAGTTACCTTCGGAAAAAGAGTTGGTAGCTCTTGAT<br>CCGGCAAACAAACCACCGCTGGTAGCGGTGGTTTTTTTGTTTGCAAGCAGC<br>AGATTACGCGCAGAAAAAAAGGATCTCAAGAAGATCCTTTGATCTTTTCTAC<br>GGGGTCTGACGCTCAGTGGAACGAAAACTCACGTTAAGGGATTTTGGTCAT<br>GAGATTATCAAAAAGGATCTTCACCTAGATCCTTTTAAATTAAAAATGAAGTT<br>TTAAATCAATCTAAAGTATATATGAGTAAACTTGGTCTGACAGCTCGAGGCT<br>TGGATTCTCACCAATAAAAAACGCCCGGCGGCAACCGAGCGTTCTGAACAA<br>ATCCAGATGGAGTTCTGAGGTCATTACTGGATCTATCAACAGGAGTCCAAG<br>CGAGCTCGATATCAAATTACGCCCCGCCCTGCCACTCATCGCAGTACTGTT<br>GTAATTCATTAAGCATTCTGCCGACATGGAAGCCATCACAAACGGCATGAT<br>GAACCTGAATCGCCAGCGGCATCAGCACCTTGTCGCCTTGCGTATAATATT<br>TGCCCATGGTGAAAACGGGGGCGAAGAAGTTGTCCATATTGGCCACGTTTA<br>AATCAAAACTGGTGAAACTCACCCAGGGATTGGCTGACACGAAAAACATAT<br>TCTCAATAAACCCTTTAGGGAAATAGGCCAGGTTTTCACCGTAACACGCCA<br>CATCTTGCGAATATATGTGTAGAAACTGCCGGAAATCGTCGTGGTATTCACT<br>CCAGAGCGATGAAAACGTTTCAGTTTGCTCATGGAAAACGGTGTAACAAGG<br>GTGAACACTATCCCATATCACCAGCTCACCGTCTTTCATTGCCATACGAAAT<br>TCCGGATGAGCATTCATCAGGCGGGCAAGAATGTGAATAAAGGCCGGATAA<br>AACTTGTGCTTATTTTTCTTTACGGTCTTTAAAAAGGCCGTAATATCCAGCTG<br>AACGGTCTGGTTATAGGTACATTGAGCAACTGACTGAAATGCCTCAAAATGT<br>TCTTTACGATGCCATTGGGATATATCAACGGTGGTATATCCAGTGATTTTTTT<br>CTCCATTTTAGCTTCCTTAGCTCCTGAAAATCTCGATAACTCAAAAAATACG<br>CCCGGTAGTGATCTTATTTCATTATGGTGAAAGTTGGAACCTCTTACGTGCC<br>CGATCAACTCGAGTGCCACCTGACGTCTAAGAAACCATTATTATCATGACAT<br>TAACCTATAAAAATAGGCGTATCACGAGGCAGAATTTCAGATAAAAAAAATC<br>CTTAGCTTTCGCTAAGGATGATTTCTGGAATTCGCGGCCGCTTCTAGAGGA<br>GCTGTTGACAATTAATCATCGGCTCGTATAATGTGTGGAATTGTGAGCGGAT<br>AACAATTCTCGAGTGGAAGAGACGAGGAAAAGAGGAGAAAAGATCAATGGT<br>TTCTAAAGGTGAAGAACTGTTCACCGGTGTTGTTCCGATCCTGGTTGAACT<br>GGACGGTGACGTTAACGGTCACAAATTCTCTGTTTCTGGTGAAGGTGAAGG<br>TGACGCTACCTACGGTAAACTGACCCTGAAATTCATCTGCACCACCGGTAA<br>ACTGCCGGTTCCGTGGCCGACCCTGGTTACCACCCTGACCTACGGTGTTC<br>AGTGCTTCGCTCGTTACCCGGACCACATGAAACAGCACGACTTCTTCAAAT<br>CTGCTATGCCGGAAGGTTACGTTCAGGAACGTACCATCTTCTTCAAAGACG<br>ACGGTAACTACAAAACCCGTGCTGAAGTTAAATTCGAAGGTGACACCCTGG<br>TTAACCGTATCGAACTGAAAGGTATCGACTTCAAAGAAGATGGTAACATCCT<br>GGGTCACAAACTGGAATACAACTACAACTCTCACAAAGTTTACATCACCGCT<br>GACAAACAGAAAAACGGTATCAAAGTTAACTTCAAAACCCGTCACAACATCG

	AAGATGGTTCTGTTCAGCTGGCTGACCACTACCAGCAGAACACCCCGATCG<br>GTGACGGTCCGGTTCTGCTGCCGGACAACCACTACCTGTCTACCCAGTCTA<br>AACTGTCTAAAGACCCGAACGAAAAACGTGACCACATGGTTCTGCTGGAAT<br>TTGTTACCGCTGCTGGTATCACCCTGGGTATGGACGAACTGTACAAATAAG<br>AGAGCAGATCGTCTCAGCAGGCATGCCCAGGCATCAAATAAAACGAAAGG<br>CTCAGTCGAAAGACTGGGCCTTTCGTTTTATCTGTTGTTTGTCGGTGAACGC<br>TCTCTACTAGAGTCACACTGGCTCACCTTCGGGTGGGCCTTTCTGCGTTTA<br>TAACGTACGTACGTACGTACGTTGGAGAGAGGATCCCTTGGAAAGTCTTCA<br>CTTGACGGCTAGCTCAGTCCTAGGTACAGTGCTAGCAATTAAAGAGGAGAA<br>CAGCTATGACCATGATCACCCCGAGCCTGCACGCTTGCCGTTCTACCCTGG<br>AAGATCCGCGTGTTCCGAGCTCTAACTCTCTGGCTGTTGTTCTGCAACGTC<br>GTGACTGGGAAAACCCGGGTGTTACCCAGCTGAACCGTCTGGCTGCTCAC<br>CCGCCGTTCGCTTCTTGGCGTAACTCTGAAGAAGCTCGTACCGACCGTCCG<br>AGCCAGCAGCTGCGTTCTCTGAACGGTGAATGGCGTCTGATGCGTTACTTC<br>CTGCTGACCCACCTGTGCGGTATCTCTCACCGTATCTGGTGCACCCTGTCT<br>ACCATCTGCTCTGACGCTGCTTAAGCCAGGCATCAAATAAAACGAAAGGCT<br>CAGTCGAAAGACTGGGCCTTTCGTTTTATCTGTTGTTTGTCGGTGAACGCTC<br>TCTACTAGAGTCACACTGGCTCACCTTCGGGTGGGCCTTTCTGCGTTTATAT<br>GAAGACAGGCAGCCATGGGAGAGCAGGGTACAAAGAGGAGAAATACTAGA<br>TGGTTTCTAAAGGTGAAGAAGATAACATGGCTATCATCAAAGAATTTATGCG<br>TTTCAAAGTTCACATGGAAGGTTCTGTTAACGGTCACGAATTTGAAATCGAA<br>GGTGAAGGTGAAGGTCGTCCGTACGAAGGTACCCAGACCGCTAAACTGAA<br>AGTTACCAAAGGTGGTCCGCTGCCGTTCGCTTGGGACATCCTGTCTCCGCA<br>GTTCATGTACGGTTCTAAAGCGTACGTTAAACACCCGGCTGACATCCCGGA<br>CTACCTGAAACTGTCTTTCCCGGAAGGTTTCAAATGGGAACGTGTTATGAAC<br>TTCGAAGATGGTGGTGTTGTTACCGTTACCCAGGACTCTTCTCTGCAAGAC<br>GGTGAATTTATCTACAAAGTTAAACTGCGTGGTACCAACTTCCCGTCTGACG<br>GTCCGGTTATGCAGAAAAAAACTATGGGTTGGGAAGCGAGCTCTGAACGTA<br>TGTACCCGGAAGATGGTGCTCTGAAAGGTGAAATCAAACAGCGTCTGAAAC<br>TGAAAGACGGTGGTCACTACGACGCTGAAGTTAAAACCACCTACAAAGCTA<br>AAAAACCGGTTCAGCTGCCGGGTGCTTACAACGTTAACATCAAACTGGACA<br>TCACCTCTCACAACGAAGATTACACCATCGTTGAACAGTACGAACGTGCTG<br>AAGGTCGTCACTCTACCGGTGGTATGGACGAACTGTACAAATAATCCAGGC<br>ATCAAATAAAACGAAAGGCTCAGTCGAAAGACTGGGCCTTTCGTTTTATCTG<br>TTGTTTGTCGGTGAACGCTCTCTACTAGAGTCACACTGGCTCACCTTCGGG<br>TGGGCCTTTCTGCGTTTATATGTTTACTAGTAGCGGCCGCTGCAG

Sequence name	**Psicose biosensor based on pPsiA promoter from *Agrobacterium tumefaciens* and the PsiR transcription factor from *Agrobacterium tumefaciens* with mCherry as reporter gene**
Acc. number	iGEM Parts Registry: BBa_K2448025<br>http://parts.igem.org/Part:BBa_K2448025
Sequence	TCCGGCAAAAAAGGGCAAGGTGTCACCACCCTGCCCTTTTTCTTTAAAACC<br>GAAAAGATTACTTCGCGTTATGCAGGCTTCCTCGCTCACTGACTCGCTGCG<br>CTCGGTCGTTCGGCTGCGGCGAGCGGTATCAGCTCACTCAAAGGCGGTAA<br>TACGGTTATCCACAGAATCAGGGGATAACGCAGGAAAGAACATGTGAGCAA<br>AAGGCCAGCAAAAGGCCAGGAACCGTAAAAAGGCCGCGTTGCTGGCGTTT<br>TTCCACAGGCTCCGCCCCCCTGACGAGCATCACAAAAATCGACGCTCAAGT<br>CAGAGGTGGCGAAACCCGACAGGACTATAAAGATACCAGGCGTTTCCCCC<br>TGGAAGCTCCCTCGTGCGCTCTCCTGTTCCGACCCTGCCGCTTACCGGATA<br>CCTGTCCGCCTTTCTCCCTTCGGGAAGCGTGGCGCTTTCTCATAGCTCACG<br>CTGTAGGTATCTCAGTTCGGTGTAGGTCGTTCGCTCCAAGCTGGGCTGTGT<br>GCACGAACCCCCCGTTCAGCCCGACCGCTGCGCCTTATCCGGTAACTATC<br>GTCTTGAGTCCAACCCGGTAAGACACGACTTATCGCCACTGGCAGCAGCCA<br>CTGGTAACAGGATTAGCAGAGCGAGGTATGTAGGCGGTGCTACAGAGTTCT<br>TGAAGTGGTGGCCTAACTACGGCTACACTAGAAGAACAGTATTTGGTATCT

	GCGCTCTGCTGAAGCCAGTTACCTTCGGAAAAAGAGTTGGTAGCTCTTGAT<br>CCGGCAAACAAACCACCGCTGGTAGCGGTGGTTTTTTTGTTTGCAAGCAGC<br>AGATTACGCGCAGAAAAAAAGGATCTCAAGAAGATCCTTTGATCTTTTCTAC<br>GGGGTCTGACGCTCAGTGGAACGAAAACTCACGTTAAGGGATTTTGGTCAT<br>GAGATTATCAAAAAGGATCTTCACCTAGATCCTTTTAAATTAAAAATGAAGTT<br>TTAAATCAATCTAAAGTATATATGAGTAAACTTGGTCTGACAGCTCGAGGCT<br>TGGATTCTCACCAATAAAAAACGCCCGGCGGCAACCGAGCGTTCTGAACAA<br>ATCCAGATGGAGTTCTGAGGTCATTACTGGATCTATCAACAGGAGTCCAAG<br>CGAGCTCGATATCAAATTACGCCCCGCCCTGCCACTCATCGCAGTACTGTT<br>GTAATTCATTAAGCATTCTGCCGACATGGAAGCCATCACAAACGGCATGAT<br>GAACCTGAATCGCCAGCGGCATCAGCACCTTGTCGCCTTGCGTATAATATT<br>TGCCCATGGTGAAAACGGGGGCGAAGAAGTTGTCCATATTGGCCACGTTTA<br>AATCAAAACTGGTGAAACTCACCCAGGGATTGGCTGACACGAAAAACATAT<br>TCTCAATAAACCCTTTAGGGAAATAGGCCAGGTTTTCACCGTAACACGCCA<br>CATCTTGCGAATATATGTGTAGAAACTGCCGGAAATCGTCGTGGTATTCACT<br>CCAGAGCGATGAAAACGTTTCAGTTTGCTCATGGAAAACGGTGTAACAAGG<br>GTGAACACTATCCCATATCACCAGCTCACCGTCTTTCATTGCCATACGAAAT<br>TCCGGATGAGCATTCATCAGGCGGGCAAGAATGTGAATAAAGGCCGGATAA<br>AACTTGTGCTTATTTTTCTTTACGGTCTTTAAAAAGGCCGTAATATCCAGCTG<br>AACGGTCTGGTTATAGGTACATTGAGCAACTGACTGAAATGCCTCAAAATGT<br>TCTTTACGATGCCATTGGGATATATCAACGGTGGTATATCCAGTGATTTTTTT<br>CTCCATTTTAGCTTCCTTAGCTCCTGAAAATCTCGATAACTCAAAAAATACG<br>CCCGGTAGTGATCTTATTTCATTATGGTGAAAGTTGGAACCTCTTACGTGCC<br>CGATCAACTCGAGTGCCACCTGACGTCTAAGAAACCATTATTATCATGACAT<br>TAACCTATAAAAATAGGCGTATCACGAGGCAGAATTTCAGATAAAAAAAATC<br>CTTAGCTTTCGCTAAGGATGATTTCTGGAATTCGCGGCCGCTTCTAGAGGA<br>GCTGTTGACAATTAATCATCGGCTCGTATAATGTGTGGAATTGTGAGCGGAT<br>AACAATTCTCGAGTGGAAGAGACGGTACAAAGAGGAGAAATACCATATGAC<br>CGGTATCTCTTCTAAAAAAGCTACCATCTACGACCTGTCTATCCTGTCTGGT<br>GCTTCTGCTTCTACCGTTTCTGCTGTTCTGAACGGTTCTTGGCGTAAACGTC<br>GTATCTCTGAAGAAACCGCTGACAAAATCCTGTCTCTGGCTAAAGCTCAGC<br>GTTACACCACCAACTTACAGGCTCGTGGTCTGCGTTCTTCTAAATCTGGTCT<br>GGTTGGTCTGCTGGTTCCGGTTTACGACAACCGTTTCTTCTCTTCTATGGCT<br>CAGACCTTCGAAGGTCAGGCTCGTAAACGTGGTCTGTCTCCGATGGTTGTT<br>TCTGGTCGTCGTGACCCGGAAGAAGAACGTCGTACCGTTGAAACCCTGATC<br>GCTTACTCTATCGACGCTCTGTTCATCGCTGGTGTTACCGACCCGGACGGT<br>GTTCACCAGGTTTGCGCTCGTGCTGCTCTGCCGCACGTTAACATCGACCTG<br>CCGGGTAAATTCGCTTCTTCTGTTATCTCTAACAACCGTCACGGTGCTGAAA<br>TCCTGACCGCTGCTATCCTGGCTCACGCTGCTAAAGGTGGTTCTCTGGGTC<br>CGGACGACGTTATCCTGTTCGGTGGTCACGACGACCACGCTTCTCGTGAAC<br>GTATCGACGGTTTCCACGCTGCTAAAGCTGACTACTTCGGTGTTGAAGGTG<br>GTGACGACATCGAAATCACCGGTTACTCTCCGCACATGACCGAAATGGCTT<br>TCGAACGTTTCTTCGGTCGTCGTGGTCGTCTGCCGCGTTGCTTCTTCGTTA<br>ACTCTTCTATCAACTTCGAAGGTCTGCTGCGTTTCATGGGTCGTCACGACG<br>GTGAAGCTTTCGGTGACATCGTTGTTGGTTGCTTCGACTACGACCCGTTCG<br>CTTCTTTCCTGCCGTTCCCGGTTTACATGATCAAACCGGACATCGCTCAGAT<br>GCTGGAAAAAGGTTTCGAACTGCTGGAAGAAAACCGTACCGAACCGGAAGT<br>TACCATCATCGAACCGCAGCTGATCCCGCCGCGTACCGCTCTGGAAGGTC<br>CGCTGGACGACATCTGGGACCCGGTTGCTCTGCGTCGTATGGCTAAATAAA<br>GCAGGCATGCCCAGGCATCAAATAAAACGAAAGGCTCAGTCGAAAGACTG<br>GGCCTTTCGTTTTATCTGTTGTTTGTCGGTGAACGCTCTCTACTAGAGTCAC<br>ACTGGCTCACCTTCGGGTGGGCCTTTCTGCGTTTATAACGTACGTACGTAC<br>GTGGATCCCTTGGAGTATAAATGGTGGCTTTTTTTGAACTTATGCCCGTCAC<br>TGTGATCTCCCCAACTGATTCCGATTATTAGAGCACGCATCCCCTTGACGG<br>AAGGGCGCTTCATGATATGGTTATTGCACCATCGATTGTGCAGATTGGCAA<br>TATCGATTGTGCATGGTGGTTGCTATGGGAGTGGCAAGGGAGAGTCTCGAA<br>TAAGCGAGATGAGAGATTTTGAACGCGTCCGGGAAAAACGGGCTGCGGGC<br>GGATTTCGTTTGCCGAATTTTTGAGGAGGAACATCAATGAAGAAAATTATTG<br>CTGCGGCGGTTGGTCTGTCGCTGGCGTTGCTCTCATCCGCAGCCTTTGCC

	GAAGGGCCGAAGGTGGGCGTCGTCGTCAAGATCGGCGGCATTCCGTGGTT<br>CAACGCCAGCAGCCATGGGTACAAAGAGGAGAAATACTAGATGGTTTCTAA<br>AGGTGAAGAAGATAACATGGCTATCATCAAAGAATTTATGCGTTTCAAAGTT<br>CACATGGAAGGTTCTGTTAACGGTCACGAATTTGAAATCGAAGGTGAAGGT<br>GAAGGTCGTCCGTACGAAGGTACCCAGACCGCTAAACTGAAAGTTACCAAA<br>GGTGGTCCGCTGCCGTTCGCTTGGGACATCCTGTCTCCGCAGTTCATGTAC<br>GGTTCTAAAGCGTACGTTAAACACCCGGCTGACATCCCGGACTACCTGAAA<br>CTGTCTTTCCCGGAAGGTTTCAAATGGGAACGTGTTATGAACTTCGAAGAT<br>GGTGGTGTTGTTACCGTTACCCAGGACTCTTCTCTGCAAGACGGTGAATTT<br>ATCTACAAAGTTAAACTGCGTGGTACCAACTTCCCGTCTGACGGTCCGGTT<br>ATGCAGAAAAAAACTATGGGTTGGGAAGCGAGCTCTGAACGTATGTACCCG<br>GAAGATGGTGCTCTGAAAGGTGAAATCAAACAGCGTCTGAAACTGAAAGAC<br>GGTGGTCACTACGACGCTGAAGTTAAAACCACCTACAAAGCTAAAAAACCG<br>GTTCAGCTGCCGGGTGCTTACAACGTTAACATCAAACTGGACATCACCTCT<br>CACAACGAAGATTACACCATCGTTGAACAGTACGAACGTGCTGAAGGTCGT<br>CACTCTACCGGTGGTATGGACGAACTGTACAAATAATCCAGGCATCAAATA<br>AAACGAAAGGCTCAGTCGAAAGACTGGGCCTTTCGTTTTATCTGTTGTTTGT<br>CGGTGAACGCTCTCTACTAGAGTCACACTGGCTCACCTTCGGGTGGGCCTT<br>TCTGCGTTTATATGTTTACTAGTAGCGGCCGCTGCAG

Sequence name	**Psicose biosensor based on pPsiR promoter from *Agrobacterium tumefaciens* and the PsiR transcription factor from *Agrobacterium tumefaciens* with mCherry as reporter gene**
Acc. number	iGEM Parts Registry: BBa_K2448026<br>http://parts.igem.org/Part:BBa_K2448026
Sequence	TCCGGCAAAAAAGGGCAAGGTGTCACCACCCTGCCCTTTTTCTTTAAAACC<br>GAAAAGATTACTTCGCGTTATGCAGGCTTCCTCGCTCACTGACTCGCTGCG<br>CTCGGTCGTTCGGCTGCGGCGAGCGGTATCAGCTCACTCAAAGGCGGTAA<br>TACGGTTATCCACAGAATCAGGGGATAACGCAGGAAAGAACATGTGAGCAA<br>AAGGCCAGCAAAAGGCCAGGAACCGTAAAAAGGCCGCGTTGCTGGCGTTT<br>TTCCACAGGCTCCGCCCCCCTGACGAGCATCACAAAAATCGACGCTCAAGT<br>CAGAGGTGGCGAAACCCGACAGGACTATAAAGATACCAGGCGTTTCCCCC<br>TGGAAGCTCCCTCGTGCGCTCTCCTGTTCCGACCCTGCCGCTTACCGGATA<br>CCTGTCCGCCTTTCTCCCTTCGGGAAGCGTGGCGCTTTCTCATAGCTCACG<br>CTGTAGGTATCTCAGTTCGGTGTAGGTCGTTCGCTCCAAGCTGGGCTGTGT<br>GCACGAACCCCCCGTTCAGCCCGACCGCTGCGCCTTATCCGGTAACTATC<br>GTCTTGAGTCCAACCCGGTAAGACACGACTTATCGCCACTGGCAGCAGCCA<br>CTGGTAACAGGATTAGCAGAGCGAGGTATGTAGGCGGTGCTACAGAGTTCT<br>TGAAGTGGTGGCCTAACTACGGCTACACTAGAAGAACAGTATTTGGTATCT<br>GCGCTCTGCTGAAGCCAGTTACCTTCGGAAAAAGAGTTGGTAGCTCTTGAT<br>CCGGCAAACAAACCACCGCTGGTAGCGGTGGTTTTTTTGTTTGCAAGCAGC<br>AGATTACGCGCAGAAAAAAAGGATCTCAAGAAGATCCTTTGATCTTTTCTAC<br>GGGGTCTGACGCTCAGTGGAACGAAAACTCACGTTAAGGGATTTTGGTCAT<br>GAGATTATCAAAAAGGATCTTCACCTAGATCCTTTTAAATTAAAAATGAAGTT<br>TTAAATCAATCTAAAGTATATATGAGTAAACTTGGTCTGACAGCTCGAGGCT<br>TGGATTCTCACCAATAAAAAACGCCCGGCGGCAACCGAGCGTTCTGAACAA<br>ATCCAGATGGAGTTCTGAGGTCATTACTGGATCTATCAACAGGAGTCCAAG<br>CGAGCTCGATATCAAATTACGCCCCGCCCTGCCACTCATCGCAGTACTGTT<br>GTAATTCATTAAGCATTCTGCCGACATGGAAGCCATCACAAACGGCATGAT<br>GAACCTGAATCGCCAGCGGCATCAGCACCTTGTCGCCTTGCGTATAATATT<br>TGCCCATGGTGAAAACGGGGGCGAAGAAGTTGTCCATATTGGCCACGTTTA<br>AATCAAAACTGGTGAAACTCACCCAGGGATTGGCTGACACGAAAAACATAT<br>TCTCAATAAACCCTTTAGGGAAATAGGCCAGGTTTTCACCGTAACACGCCA<br>CATCTTGCGAATATATGTGTAGAAACTGCCGGAAATCGTCGTGGTATTCACT<br>CCAGAGCGATGAAAACGTTTCAGTTTGCTCATGGAAAACGGTGTAACAAGG<br>GTGAACACTATCCCATATCACCAGCTCACCGTCTTTCATTGCCATACGAAAT

	TCCGGATGAGCATTCATCAGGCGGGCAAGAATGTGAATAAAGGCCGGATAA<br>AACTTGTGCTTATTTTTCTTTACGGTCTTTAAAAAGGCCGTAATATCCAGCTG<br>AACGGTCTGGTTATAGGTACATTGAGCAACTGACTGAAATGCCTCAAAATGT<br>TCTTTACGATGCCATTGGGATATATCAACGGTGGTATATCCAGTGATTTTTTT<br>CTCCATTTTAGCTTCCTTAGCTCCTGAAAATCTCGATAACTCAAAAAATACG<br>CCCGGTAGTGATCTTATTTCATTATGGTGAAAGTTGGAACCTCTTACGTGCC<br>CGATCAACTCGAGTGCCACCTGACGTCTAAGAAACCATTATTATCATGACAT<br>TAACCTATAAAAATAGGCGTATCACGAGGCAGAATTTCAGATAAAAAAAATC<br>CTTAGCTTTCGCTAAGGATGATTTCTGGAATTCGCGGCCGCTTCTAGAGGA<br>GCTGTTGACAATTAATCATCGGCTCGTATAATGTGTGGAATTGTGAGCGGAT<br>AACAATTCTCGAGTGGAAGAGACGGTACAAAGAGGAGAAATACCATATGAC<br>CGGTATCTCTTCTAAAAAAGCTACCATCTACGACCTGTCTATCCTGTCTGGT<br>GCTTCTGCTTCTACCGTTTCTGCTGTTCTGAACGGTTCTTGGCGTAAACGTC<br>GTATCTCTGAAGAAACCGCTGACAAAATCCTGTCTCTGGCTAAAGCTCAGC<br>GTTACACCACCAACTTACAGGCTCGTGGTCTGCGTTCTTCTAAATCTGGTCT<br>GGTTGGTCTGCTGGTTCCGGTTTACGACAACCGTTTCTTCTCTTCTATGGCT<br>CAGACCTTCGAAGGTCAGGCTCGTAAACGTGGTCTGTCTCCGATGGTTGTT<br>TCTGGTCGTCGTGACCCGGAAGAAGAACGTCGTACCGTTGAAACCCTGATC<br>GCTTACTCTATCGACGCTCTGTTCATCGCTGGTGTTACCGACCCGGACGGT<br>GTTCACCAGGTTTGCGCTCGTGCTGCTCTGCCGCACGTTAACATCGACCTG<br>CCGGGTAAATTCGCTTCTTCTGTTATCTCTAACAACCGTCACGGTGCTGAAA<br>TCCTGACCGCTGCTATCCTGGCTCACGCTGCTAAAGGTGGTTCTCTGGGTC<br>CGGACGACGTTATCCTGTTCGGTGGTCACGACGACCACGCTTCTCGTGAAC<br>GTATCGACGGTTTCCACGCTGCTAAAGCTGACTACTTCGGTGTTGAAGGTG<br>GTGACGACATCGAAATCACCGGTTACTCTCCGCACATGACCGAAATGGCTT<br>TCGAACGTTTCTTCGGTCGTCGTGGTCGTCTGCCGCGTTGCTTCTTCGTTA<br>ACTCTTCTATCAACTTCGAAGGTCTGCTGCGTTTCATGGGTCGTCACGACG<br>GTGAAGCTTTCGGTGACATCGTTGTTGGTTGCTTCGACTACGACCCGTTCG<br>CTTCTTTCCTGCCGTTCCCGGTTTACATGATCAAACCGGACATCGCTCAGAT<br>GCTGGAAAAAGGTTTCGAACTGCTGGAAGAAAACCGTACCGAACCGGAAGT<br>TACCATCATCGAACCGCAGCTGATCCCGCCGCGTACCGCTCTGGAAGGTC<br>CGCTGGACGACATCTGGGACCCGGTTGCTCTGCGTCGTATGGCTAAATAAA<br>GCAGGCATGCCCAGGCATCAAATAAAACGAAAGGCTCAGTCGAAAGACTG<br>GGCCTTTCGTTTTATCTGTTGTTTGTCGGTGAACGCTCTCTACTAGAGTCAC<br>ACTGGCTCACCTTCGGGTGGGCCTTTCTGCGTTTATAACGTACGTACGTAC<br>GTGGATCCCTTGGAGGAGGCGTTGAACCACGGAATGCCGCCGATCTTGAC<br>GACGACGCCCACCTTCGGCCCTTCGGCAAAGGCTGCGGATGAGAGCAACG<br>CCAGCGACAGACCAACCGCCGCAGCAATAATTTTCTTCATTGATGTTCCTC<br>CTCAAAAATTCGGCAAACGAAATCCGCCCGCAGCCCGTTTTTCCCGGACGC<br>GTTCAAAATCTCTCATCTCGCTTATTCGAGACTCTCCCTTGCCACTCCCATA<br>GCAACCACCATGCACAATCGATATTGCCAATCTGCACAATCGATGGTGCAA<br>TAACCATATCATGAAGCGCCCTTCCGTCAAGGGGATGCGTGCTCTAATAAT<br>CGGAATCAGTTGGGGAGATCACATAGCAGCCATGGGTACAAAGAGGAGAA<br>ATACTAGATGGTTTCTAAAGGTGAAGAAGATAACATGGCTATCATCAAAGAA<br>TTTATGCGTTTCAAAGTTCACATGGAAGGTTCTGTTAACGGTCACGAATTTG<br>AAATCGAAGGTGAAGGTGAAGGTCGTCCGTACGAAGGTACCCAGACCGCT<br>AAACTGAAAGTTACCAAAGGTGGTCCGCTGCCGTTCGCTTGGGACATCCTG<br>TCTCCGCAGTTCATGTACGGTTCTAAAGCGTACGTTAAACACCCGGCTGAC<br>ATCCCGGACTACCTGAAACTGTCTTTCCCGGAAGGTTTCAAATGGGAACGT<br>GTTATGAACTTCGAAGATGGTGGTGTTGTTACCGTTACCCAGGACTCTTCTC<br>TGCAAGACGGTGAATTTATCTACAAAGTTAAACTGCGTGGTACCAACTTCCC<br>GTCTGACGGTCCGGTTATGCAGAAAAAAACTATGGGTTGGGAAGCGAGCTC<br>TGAACGTATGTACCCGGAAGATGGTGCTCTGAAAGGTGAAATCAAACAGCG<br>TCTGAAACTGAAAGACGGTGGTCACTACGACGCTGAAGTTAAAACCACCTA<br>CAAAGCTAAAAAACCGGTTCAGCTGCCGGGTGCTTACAACGTTAACATCAA<br>ACTGGACATCACCTCTCACAACGAAGATTACACCATCGTTGAACAGTACGA<br>ACGTGCTGAAGGTCGTCACTCTACCGGTGGTATGGACGAACTGTACAAATA<br>ATCCAGGCATCAAATAAAACGAAAGGCTCAGTCGAAAGACTGGGCCTTTCG<br>TTTTATCTGTTGTTTGTCGGTGAACGCTCTCTACTAGAGTCACACTGGCTCA

	CCTTCGGGTGGGCCTTTCTGCGTTTATATGTTTACTAGTAGCGGCCGCTGC<br>AG

Sequence name	**Psicose biosensor based on pPsiTacI synthetic promoter and the PsiR transcription factor from *Agrobacterium tumefaciens* with mCherry as reporter gene**
Acc. number	iGEM Parts Registry: BBa_K2448027<br>http://parts.igem.org/Part:BBa_K2448027
Sequence	TCCGGCAAAAAAGGGCAAGGTGTCACCACCCTGCCCTTTTTCTTTAAAACC<br>GAAAAGATTACTTCGCGTTATGCAGGCTTCCTCGCTCACTGACTCGCTGCG<br>CTCGGTCGTTCGGCTGCGGCGAGCGGTATCAGCTCACTCAAAGGCGGTAA<br>TACGGTTATCCACAGAATCAGGGGATAACGCAGGAAAGAACATGTGAGCAA<br>AAGGCCAGCAAAAGGCCAGGAACCGTAAAAAGGCCGCGTTGCTGGCGTTT<br>TTCCACAGGCTCCGCCCCCCTGACGAGCATCACAAAAATCGACGCTCAAGT<br>CAGAGGTGGCGAAACCCGACAGGACTATAAAGATACCAGGCGTTTCCCCC<br>TGGAAGCTCCCTCGTGCGCTCTCCTGTTCCGACCCTGCCGCTTACCGGATA<br>CCTGTCCGCCTTTCTCCCTTCGGGAAGCGTGGCGCTTTCTCATAGCTCACG<br>CTGTAGGTATCTCAGTTCGGTGTAGGTCGTTCGCTCCAAGCTGGGCTGTGT<br>GCACGAACCCCCCGTTCAGCCCGACCGCTGCGCCTTATCCGGTAACTATC<br>GTCTTGAGTCCAACCCGGTAAGACACGACTTATCGCCACTGGCAGCAGCCA<br>CTGGTAACAGGATTAGCAGAGCGAGGTATGTAGGCGGTGCTACAGAGTTCT<br>TGAAGTGGTGGCCTAACTACGGCTACACTAGAAGAACAGTATTTGGTATCT<br>GCGCTCTGCTGAAGCCAGTTACCTTCGGAAAAAGAGTTGGTAGCTCTTGAT<br>CCGGCAAACAAACCACCGCTGGTAGCGGTGGTTTTTTTGTTTGCAAGCAGC<br>AGATTACGCGCAGAAAAAAAGGATCTCAAGAAGATCCTTTGATCTTTTCTAC<br>GGGGTCTGACGCTCAGTGGAACGAAAACTCACGTTAAGGGATTTTGGTCAT<br>GAGATTATCAAAAAGGATCTTCACCTAGATCCTTTTAAATTAAAAATGAAGTT<br>TTAAATCAATCTAAAGTATATATGAGTAAACTTGGTCTGACAGCTCGAGGCT<br>TGGATTCTCACCAATAAAAAACGCCCGGCGGCAACCGAGCGTTCTGAACAA<br>ATCCAGATGGAGTTCTGAGGTCATTACTGGATCTATCAACAGGAGTCCAAG<br>CGAGCTCGATATCAAATTACGCCCCGCCCTGCCACTCATCGCAGTACTGTT<br>GTAATTCATTAAGCATTCTGCCGACATGGAAGCCATCACAAACGGCATGAT<br>GAACCTGAATCGCCAGCGGCATCAGCACCTTGTCGCCTTGCGTATAATATT<br>TGCCCATGGTGAAAACGGGGGCGAAGAAGTTGTCCATATTGGCCACGTTTA<br>AATCAAAACTGGTGAAACTCACCCAGGGATTGGCTGACACGAAAAACATAT<br>TCTCAATAAACCCTTTAGGGAAATAGGCCAGGTTTTCACCGTAACACGCCA<br>CATCTTGCGAATATATGTGTAGAAACTGCCGGAAATCGTCGTGGTATTCACT<br>CCAGAGCGATGAAAACGTTTCAGTTTGCTCATGGAAAACGGTGTAACAAGG<br>GTGAACACTATCCCATATCACCAGCTCACCGTCTTTCATTGCCATACGAAAT<br>TCCGGATGAGCATTCATCAGGCGGGCAAGAATGTGAATAAAGGCCGGATAA<br>AACTTGTGCTTATTTTTCTTTACGGTCTTTAAAAAGGCCGTAATATCCAGCTG<br>AACGGTCTGGTTATAGGTACATTGAGCAACTGACTGAAATGCCTCAAAATGT<br>TCTTTACGATGCCATTGGGATATATCAACGGTGGTATATCCAGTGATTTTTTT<br>CTCCATTTTAGCTTCCTTAGCTCCTGAAAATCTCGATAACTCAAAAAATACG<br>CCCGGTAGTGATCTTATTTCATTATGGTGAAAGTTGGAACCTCTTACGTGCC<br>CGATCAACTCGAGTGCCACCTGACGTCTAAGAAACCATTATTATCATGACAT<br>TAACCTATAAAAATAGGCGTATCACGAGGCAGAATTTCAGATAAAAAAAATC<br>CTTAGCTTTCGCTAAGGATGATTTCTGGAATTCGCGGCCGCTTCTAGAGGA<br>GCTGTTGACAATTAATCATCGGCTCGTATAATGTGTGGAATTGTGAGCGGAT<br>AACAATTCTCGAGTGGAAGAGACGGTACAAAGAGGAGAAATACCATATGAC<br>CGGTATCTCTTCTAAAAAAGCTACCATCTACGACCTGTCTATCCTGTCTGGT<br>GCTTCTGCTTCTACCGTTTCTGCTGTTCTGAACGGTTCTTGGCGTAAACGTC<br>GTATCTCTGAAGAAACCGCTGACAAAATCCTGTCTCTGGCTAAAGCTCAGC<br>GTTACACCACCAACTTACAGGCTCGTGGTCTGCGTTCTTCTAAATCTGGTCT<br>GGTTGGTCTGCTGGTTCCGGTTTACGACAACCGTTTCTTCTCTTCTATGGCT<br>CAGACCTTCGAAGGTCAGGCTCGTAAACGTGGTCTGTCTCCGATGGTTGTT

	TCTGGTCGTCGTGACCCGGAAGAAGAACGTCGTACCGTTGAAACCCTGATC GCTTACTCTATCGACGCTCTGTTCATCGCTGGTGTTACCGACCCGGACGGT GTTCACCAGGTTTGCGCTCGTGCTGCTCTGCCGCACGTTAACATCGACCTG CCGGGTAAATTCGCTTCTTCTGTTATCTCTAACAACCGTCACGGTGCTGAAA TCCTGACCGCTGCTATCCTGGCTCACGCTGCTAAAGGTGGTTCTCTGGGTC CGGACGACGTTATCCTGTTCGGTGGTCACGACGACCACGCTTCTCGTGAAC GTATCGACGGTTTCCACGCTGCTAAAGCTGACTACTTCGGTGTTGAAGGTG GTGACGACATCGAAATCACCGGTTACTCTCCGCACATGACCGAAATGGCTT TCGAACGTTTCTTCGGTCGTCGTGGTCGTCTGCCGCGTTGCTTCTTCGTTA ACTCTTCTATCAACTTCGAAGGTCTGCTGCGTTTCATGGGTCGTCACGACG GTGAAGCTTTCGGTGACATCGTTGTTGGTTGCTTCGACTACGACCCGTTCG CTTCTTTCCTGCCGTTCCCGGTTTACATGATCAAACCGGACATCGCTCAGAT GCTGGAAAAAGGTTTCGAACTGCTGGAAGAAAACCGTACCGAACCGGAAGT TACCATCATCGAACCGCAGCTGATCCCGCCGCGTACCGCTCTGGAAGGTC CGCTGGACGACATCTGGGACCCGGTTGCTCTGCGTCGTATGGCTAAATAAA GCAGGCATGCCCAGGCATCAAATAAAACGAAAGGCTCAGTCGAAAGACTG GGCCTTTCGTTTTATCTGTTGTTTGTCGGTGAACGCTCTCTACTAGAGTCAC ACTGGCTCACCTTCGGGTGGGCCTTTCTGCGTTTATAACGTACGTACGTAC GTGGATCCCTTGGATGAGCTGTTGACAATTAATCATCGGCTCGTATAATGTG TGGATTGCACAATCGATGGTGCAAAGCAGCCATGGGTACAAAGAGGAGAAA TACTAGATGGTTTCTAAAGGTGAAGAAGATAACATGGCTATCATCAAAGAAT TTATGCGTTTCAAAGTTCACATGGAAGGTTCTGTTAACGGTCACGAATTTGA AATCGAAGGTGAAGGTGAAGGTCGTCCGTACGAAGGTACCCAGACCGCTA AACTGAAAGTTACCAAAGGTGGTCCGCTGCCGTTCGCTTGGGACATCCTGT CTCCGCAGTTCATGTACGGTTCTAAAGCGTACGTTAAACACCCGGCTGACA TCCCGGACTACCTGAAACTGTCTTTCCCGGAAGGTTTCAAATGGGAACGTG TTATGAACTTCGAAGATGGTGGTGTTGTTACCGTTACCCAGGACTCTTCTCT GCAAGACGGTGAATTTATCTACAAAGTTAAACTGCGTGGTACCAACTTCCC GTCTGACGGTCCGGTTATGCAGAAAAAAACTATGGGTTGGGAAGCGAGCTC TGAACGTATGTACCCGGAAGATGGTGCTCTGAAAGGTGAAATCAAACAGCG TCTGAAACTGAAAGACGGTGGTCACTACGACGCTGAAGTTAAAACCACCTA CAAAGCTAAAAAACCGGTTCAGCTGCCGGGTGCTTACAACGTTAACATCAA ACTGGACATCACCTCTCACAACGAAGATTACACCATCGTTGAACAGTACGA ACGTGCTGAAGGTCGTCACTCTACCGGTGGTATGGACGAACTGTACAAATA ATCCAGGCATCAAATAAAACGAAAGGCTCAGTCGAAAGACTGGGCCTTTCG TTTTATCTGTTGTTTGTCGGTGAACGCTCTCTACTAGAGTCACACTGGCTCA CCTTCGGGTGGGCCTTTCTGCGTTTATATGTTTACTAGTAGCGGCCGCTGC AG

Sequence name	**Psicose biosensor based on pPsiA promoter from *Sinorhizobium fredii* and the PsiR transcription factor from *Sinorhizobium fredii* with mCherry as reporter gene**
Acc. number	iGEM Parts Registry: BBa_K2448028<br>http://parts.igem.org/Part:BBa_K2448028
Sequence	TCCGGCAAAAAAGGGCAAGGTGTCACCACCCTGCCCTTTTTCTTTAAAACC GAAAAGATTACTTCGCGTTATGCAGGCTTCCTCGCTCACTGACTCGCTGCG CTCGGTCGTTCGGCTGCGGCGAGCGGTATCAGCTCACTCAAAGGCGGTAA TACGGTTATCCACAGAATCAGGGGATAACGCAGGAAAGAACATGTGAGCAA AAGGCCAGCAAAAGGCCAGGAACCGTAAAAAGGCCGCGTTGCTGGCGTTT TTCCACAGGCTCCGCCCCCCTGACGAGCATCACAAAAATCGACGCTCAAGT CAGAGGTGGCGAAACCCGACAGGACTATAAAGATACCAGGCGTTTCCCCC TGGAAGCTCCCTCGTGCGCTCTCCTGTTCCGACCCTGCCGCTTACCGGATA CCTGTCCGCCTTTCTCCCTTCGGGAAGCGTGGCGCTTTCTCATAGCTCACG CTGTAGGTATCTCAGTTCGGTGTAGGTCGTTCGCTCCAAGCTGGGCTGTGT GCACGAACCCCCCGTTCAGCCCGACCGCTGCGCCTTATCCGGTAACTATC GTCTTGAGTCCAACCCGGTAAGACACGACTTATCGCCACTGGCAGCAGCCA

	CTGGTAACAGGATTAGCAGAGCGAGGTATGTAGGCGGTGCTACAGAGTTCT TGAAGTGGTGGCCTAACTACGGCTACACTAGAAGAACAGTATTTGGTATCT GCGCTCTGCTGAAGCCAGTTACCTTCGGAAAAAGAGTTGGTAGCTCTTGAT CCGGCAAACAAACCACCGCTGGTAGCGGTGGTTTTTTTGTTTGCAAGCAGC AGATTACGCGCAGAAAAAAAGGATCTCAAGAAGATCCTTTGATCTTTTCTAC GGGGTCTGACGCTCAGTGGAACGAAAACTCACGTTAAGGGATTTTGGTCAT GAGATTATCAAAAAGGATCTTCACCTAGATCCTTTTAAATTAAAAATGAAGTT TTAAATCAATCTAAAGTATATATGAGTAAACTTGGTCTGACAGCTCGAGGCT TGGATTCTCACCAATAAAAAACGCCCGGCGGCAACCGAGCGTTCTGAACAA ATCCAGATGGAGTTCTGAGGTCATTACTGGATCTATCAACAGGAGTCCAAG CGAGCTCGATATCAAATTACGCCCCGCCCTGCCACTCATCGCAGTACTGTT GTAATTCATTAAGCATTCTGCCGACATGGAAGCCATCACAAACGGCATGAT GAACCTGAATCGCCAGCGGCATCAGCACCTTGTCGCCTTGCGTATAATATT TGCCCATGGTGAAAACGGGGGCGAAGAAGTTGTCCATATTGGCCACGTTTA AATCAAAACTGGTGAAACTCACCCAGGGATTGGCTGACACGAAAAACATAT TCTCAATAAACCCTTTAGGGAAATAGGCCAGGTTTTCACCGTAACACGCCA CATCTTGCGAATATATGTGTAGAAACTGCCGGAAATCGTCGTGGTATTCACT CCAGAGCGATGAAAACGTTTCAGTTTGCTCATGGAAAACGGTGTAACAAGG GTGAACACTATCCCATATCACCAGCTCACCGTCTTTCATTGCCATACGAAAT TCCGGATGAGCATTCATCAGGCGGGCAAGAATGTGAATAAAGGCCGGATAA AACTTGTGCTTATTTTTCTTTACGGTCTTTAAAAAGGCCGTAATATCCAGCTG AACGGTCTGGTTATAGGTACATTGAGCAACTGACTGAAATGCCTCAAAATGT TCTTTACGATGCCATTGGGATATATCAACGGTGGTATATCCAGTGATTTTTTT CTCCATTTTAGCTTCCTTAGCTCCTGAAAATCTCGATAACTCAAAAAATACG CCCGGTAGTGATCTTATTTCATTATGGTGAAAGTTGGAACCTCTTACGTGCC CGATCAACTCGAGTGCCACCTGACGTCTAAGAAACCATTATTATCATGACAT TAACCTATAAAAATAGGCGTATCACGAGGCAGAATTTCAGATAAAAAAAATC CTTAGCTTTCGCTAAGGATGATTTCTGGAATTCGCGGCCGCTTCTAGAGGA GCTGTTGACAATTAATCATCGGCTCGTATAATGTGTGGAATTGTGAGCGGAT AACAATTCTCGAGTGGAAGAGACGGTACAAAGAGGAGAAATACCATATGGC TAACTCTGGTAAAAAAAAAGCTACCATCTACGACCTGTCTGTTCTGTCTGGT TCTTCTCCGTCTACCGTTTCTGCTGTTCTGAACGGTACCTGGCGTAAACGTC GTATCAAAGAATCTACCGCTGAACTGATCCGTAACCTGGCTGAAACCCACC AGTACACCGCTAACCGTCAGGCTCGTGGTCTGCGTTCTTCTCGTTCTGGTC TGGTTGGTCTGCTGCTGCCGGTTCACGACAACCGTTACTTCTCTTCTCTGG CTCAGACCTTCGAAGCTCACGTTCGTTCTAAAGGTCAGTGCCCGATCGTTG TTTCTGCTTCTCGTGACCCGCAGGAAGAACGTAAAACCGCTGAAACCCTGA TCTCTTACTCTATCGACGAACTGTTCATCTGCGGTGCTACCGACCCGGACG GTGTTCACGAAGTTTGCGAAGCTGCTGGTCTGAAACACATCAACATCGACC TGCCGGGTACCAAAGTTCCGTCTGTTATCTCTGACAACTTCGAAGGTGGTC GTCTGCTGACCGAAGCTATCATCCGTCACTTCCCGGCTGACCGTGCTCTGG CTCCGACCGACCTGTACCTGTTCGGTGGTCGTAACGACCACGCTTCTCACG AACGTATCCGTGGTTTCCGTGCTGTTAAAAAAGACCTGCTGGGTGACGACC CGGACGAATGCATCCAGCCGACCGGTTACGCTGCTAACAACGCTCGTAAA GCGTTCGAAGCGTTCTACGCTCGTCACGGTAAACTGCCGCGTGGTCTGTTC GTTAACTCTTCTATCAACTTCGAAGGTCTGCTGCGTTTCATGGCTGAACACC CGCACGACAACTTCACCGACCTGGTTGTTGGTTGCTACGACTACGACCCGT TCGCTTCTTTCCTGCCGTTCCCGGTTATCATGATCCGTCAGGACGTTGAAG GTATGATCGCTAAAGCGTTCGAAGTTATCGAACAGCCGCGTGCTCTGGCTC GTATCCACCTGGTTCAGCCGGAACTGGTTCCGCCGCGTACCGCTCTGACC GGTCCGCTGGACGCTCTGAAAGACATCGACCTGCCGCGTGGTTCTCAGTA AAGCAGGCATGCCCAGGCATCAAATAAAACGAAAGGCTCAGTCGAAAGACT GGGCCTTTCGTTTTATCTGTTGTTTGTCGGTGAACGCTCTCTACTAGAGTCA CACTGGCTCACCTTCGGGTGGGCCTTTCTGCGTTTATAACGTACGTACGTA CGTGGATCCCTTGGAGGTGGGTCTGGGCGAGGTTGCGGATCAACTCGGCG GTGCTTTCCTTGATGCGCCGCTTGCGCCAGGTGCCGTTCAGCACGGCGCT GACCGTCGAGGGCGAGCTGCCGGAGAGCACCGAGAGATCGTAGATCGTC GCCTTTTTCTTGCCGCTGTTCGCCATCCGAGCCCCCTCGAATCTCTTAGAG CCGTTTTGCGCTTGACGAAAGATTAAGTCTGCACGATAGTCTTTGCACCATC

	GATTGTGCAAATAAGAAATATCGATTGTGCAGCTCTTTGGGCCGTCTGAGG<br>AGGCGGCGGTCAGCGGCGGGAAACGCGCTTCTCGTCATGGAGGATTGAAA<br>CTGGAGGCCGGCGCGCCAGCGCCCGGGAGAGTTCCCGTTGCGGGAACCT<br>GTGGAGGAGAGACAGCAGCCATGGGTACAAAGAGGAGAAATACTAGATGG<br>TTTCTAAAGGTGAAGAAGATAACATGGCTATCATCAAAGAATTTATGCGTTT<br>CAAAGTTCACATGGAAGGTTCTGTTAACGGTCACGAATTTGAAATCGAAGGT<br>GAAGGTGAAGGTCGTCCGTACGAAGGTACCCAGACCGCTAAACTGAAAGTT<br>ACCAAAGGTGGTCCGCTGCCGTTCGCTTGGGACATCCTGTCTCCGCAGTTC<br>ATGTACGGTTCTAAAGCGTACGTTAAACACCCGGCTGACATCCCGGACTAC<br>CTGAAACTGTCTTTCCCGGAAGGTTTCAAATGGGAACGTGTTATGAACTTCG<br>AAGATGGTGGTGTTGTTACCGTTACCCAGGACTCTTCTCTGCAAGACGGTG<br>AATTTATCTACAAAGTTAAACTGCGTGGTACCAACTTCCCGTCTGACGGTCC<br>GGTTATGCAGAAAAAAACTATGGGTTGGGAAGCGAGCTCTGAACGTATGTA<br>CCCGGAAGATGGTGCTCTGAAAGGTGAAATCAAACAGCGTCTGAAACTGAA<br>AGACGGTGGTCACTACGACGCTGAAGTTAAAACCACCTACAAAGCTAAAAA<br>ACCGGTTCAGCTGCCGGGTGCTTACAACGTTAACATCAAACTGGACATCAC<br>CTCTCACAACGAAGATTACACCATCGTTGAACAGTACGAACGTGCTGAAGG<br>TCGTCACTCTACCGGTGGTATGGACGAACTGTACAAATAATCCAGGCATCA<br>AATAAAACGAAAGGCTCAGTCGAAAGACTGGGCCTTTCGTTTTATCTGTTGT<br>TTGTCGGTGAACGCTCTCTACTAGAGTCACACTGGCTCACCTTCGGGTGGG<br>CCTTTCTGCGTTTATATGTTTACTAGTAGCGGCCGCTGCAG

Sequence name	**Psicose biosensor based on pPsiR promoter from *Sinorhizobium fredii* and the PsiR transcription factor from *Sinorhizobium fredii* with mCherry as reporter gene**
Acc. number	iGEM Parts Registry: BBa_K2448029<br>http://parts.igem.org/Part:BBa_K2448029
Sequence	TCCGGCAAAAAAGGGCAAGGTGTCACCACCCTGCCCTTTTTCTTTAAAACC<br>GAAAAGATTACTTCGCGTTATGCAGGCTTCCTCGCTCACTGACTCGCTGCG<br>CTCGGTCGTTCGGCTGCGGCGAGCGGTATCAGCTCACTCAAAGGCGGTAA<br>TACGGTTATCCACAGAATCAGGGGATAACGCAGGAAAGAACATGTGAGCAA<br>AAGGCCAGCAAAAGGCCAGGAACCGTAAAAAGGCCGCGTTGCTGGCGTTT<br>TTCCACAGGCTCCGCCCCCCTGACGAGCATCACAAAAATCGACGCTCAAGT<br>CAGAGGTGGCGAAACCCGACAGGACTATAAAGATACCAGGCGTTTCCCCC<br>TGGAAGCTCCCTCGTGCGCTCTCCTGTTCCGACCCTGCCGCTTACCGGATA<br>CCTGTCCGCCTTTCTCCCTTCGGGAAGCGTGGCGCTTTCTCATAGCTCACG<br>CTGTAGGTATCTCAGTTCGGTGTAGGTCGTTCGCTCCAAGCTGGGCTGTGT<br>GCACGAACCCCCCGTTCAGCCCGACCGCTGCGCCTTATCCGGTAACTATC<br>GTCTTGAGTCCAACCCGGTAAGACACGACTTATCGCCACTGGCAGCAGCCA<br>CTGGTAACAGGATTAGCAGAGCGAGGTATGTAGGCGGTGCTACAGAGTTCT<br>TGAAGTGGTGGCCTAACTACGGCTACACTAGAAGAACAGTATTTGGTATCT<br>GCGCTCTGCTGAAGCCAGTTACCTTCGGAAAAAGAGTTGGTAGCTCTTGAT<br>CCGGCAAACAAACCACCGCTGGTAGCGGTGGTTTTTTTGTTTGCAAGCAGC<br>AGATTACGCGCAGAAAAAAAGGATCTCAAGAAGATCCTTTGATCTTTTCTAC<br>GGGGTCTGACGCTCAGTGGAACGAAAACTCACGTTAAGGGATTTTGGTCAT<br>GAGATTATCAAAAAGGATCTTCACCTAGATCCTTTTAAATTAAAAATGAAGTT<br>TTAAATCAATCTAAAGTATATATGAGTAAACTTGGTCTGACAGCTCGAGGCT<br>TGGATTCTCACCAATAAAAAACGCCCGGCGGCAACCGAGCGTTCTGAACAA<br>ATCCAGATGGAGTTCTGAGGTCATTACTGGATCTATCAACAGGAGTCCAAG<br>CGAGCTCGATATCAAATTACGCCCCGCCCTGCCACTCATCGCAGTACTGTT<br>GTAATTCATTAAGCATTCTGCCGACATGGAAGCCATCACAAACGGCATGAT<br>GAACCTGAATCGCCAGCGGCATCAGCACCTTGTCGCCTTGCGTATAATATT<br>TGCCCATGGTGAAAACGGGGGCGAAGAAGTTGTCCATATTGGCCACGTTTA<br>AATCAAAACTGGTGAAACTCACCCAGGGATTGGCTGACACGAAAAACATAT<br>TCTCAATAAACCCTTTAGGGAAATAGGCCAGGTTTTCACCGTAACACGCCA<br>CATCTTGCGAATATATGTGTAGAAACTGCCGGAAATCGTCGTGGTATTCACT

	CCAGAGCGATGAAAACGTTTCAGTTTGCTCATGGAAAACGGTGTAACAAGG<br>GTGAACACTATCCCATATCACCAGCTCACCGTCTTTCATTGCCATACGAAAT<br>TCCGGATGAGCATTCATCAGGCGGGCAAGAATGTGAATAAAGGCCGGATAA<br>AACTTGTGCTTATTTTTCTTTACGGTCTTTAAAAAGGCCGTAATATCCAGCTG<br>AACGGTCTGGTTATAGGTACATTGAGCAACTGACTGAAATGCCTCAAAATGT<br>TCTTTACGATGCCATTGGGATATATCAACGGTGGTATATCCAGTGATTTTTTT<br>CTCCATTTTAGCTTCCTTAGCTCCTGAAAATCTCGATAACTCAAAAAATACG<br>CCCGGTAGTGATCTTATTTCATTATGGTGAAAGTTGGAACCTCTTACGTGCC<br>CGATCAACTCGAGTGCCACCTGACGTCTAAGAAACCATTATTATCATGACAT<br>TAACCTATAAAAATAGGCGTATCACGAGGCAGAATTTCAGATAAAAAAAATC<br>CTTAGCTTTCGCTAAGGATGATTTCTGGAATTCGCGGCCGCTTCTAGAGGA<br>GCTGTTGACAATTAATCATCGGCTCGTATAATGTGTGGAATTGTGAGCGGAT<br>AACAATTCTCGAGTGGAAGAGACGGTACAAAGAGGAGAAATACCATATGGC<br>TAACTCTGGTAAAAAAAAAGCTACCATCTACGACCTGTCTGTTCTGTCTGGT<br>TCTTCTCCGTCTACCGTTTCTGCTGTTCTGAACGGTACCTGGCGTAAACGTC<br>GTATCAAAGAATCTACCGCTGAACTGATCCGTAACCTGGCTGAAACCCACC<br>AGTACACCGCTAACCGTCAGGCTCGTGGTCTGCGTTCTTCTCGTTCTGGTC<br>TGGTTGGTCTGCTGCTGCCGGTTCACGACAACCGTTACTTCTCTTCTCTGG<br>CTCAGACCTTCGAAGCTCACGTTCGTTCTAAAGGTCAGTGCCCGATCGTTG<br>TTTCTGCTTCTCGTGACCCGCAGGAAGAACGTAAAACCGCTGAAACCCTGA<br>TCTCTTACTCTATCGACGAACTGTTCATCTGCGGTGCTACCGACCCGGACG<br>GTGTTCACGAAGTTTGCGAAGCTGCTGGTCTGAAACACATCAACATCGACC<br>TGCCGGGTACCAAAGTTCCGTCTGTTATCTCTGACAACTTCGAAGGTGGTC<br>GTCTGCTGACCGAAGCTATCATCCGTCACTTCCCGGCTGACCGTGCTCTGG<br>CTCCGACCGACCTGTACCTGTTCGGTGGTCGTAACGACCACGCTTCTCACG<br>AACGTATCCGTGGTTTCCGTGCTGTTAAAAAAGACCTGCTGGGTGACGACC<br>CGGACGAATGCATCCAGCCGACCGGTTACGCTGCTAACAACGCTCGTAAA<br>GCGTTCGAAGCGTTCTACGCTCGTCACGGTAAACTGCCGCGTGGTCTGTTC<br>GTTAACTCTTCTATCAACTTCGAAGGTCTGCTGCGTTTCATGGCTGAACACC<br>CGCACGACAACTTCACCGACCTGGTTGTTGGTTGCTACGACTACGACCCGT<br>TCGCTTCTTTCCTGCCGTTCCCGGTTATCATGATCCGTCAGGACGTTGAAG<br>GTATGATCGCTAAAGCGTTCGAAGTTATCGAACAGCCGCGTGCTCTGGCTC<br>GTATCCACCTGGTTCAGCCGGAACTGGTTCCGCCGCGTACCGCTCTGACC<br>GGTCCGCTGGACGCTCTGAAAGACATCGACCTGCCGCGTGGTTCTCAGTA<br>AAGCAGGCATGCCCAGGCATCAAATAAAACGAAAGGCTCAGTCGAAAGACT<br>GGGCCTTTCGTTTTATCTGTTGTTTGTCGGTGAACGCTCTCTACTAGAGTCA<br>CACTGGCTCACCTTCGGGTGGGCCTTTCTGCGTTTATAACGTACGTACGTA<br>CGTGGATCCCTTGGAGGCCGCGCTCCTTGATGCCGACTTGCATGGCGTTG<br>AACCACGGAATGCCGCCGATCTTGACGACCACGCCGACCTTCGGCGCATC<br>CTGCGCCGCGACGGAAAAGGCACCGGCGAGCGAAAGCGAAGCCGCCAGA<br>GCGGCAGCAAGAAATGTCTTGATCATGTCTCTCCTCCACAGGTTCCCGCAA<br>CGGGAACTCTCCCGGGCGCTGGCGCGCCGGCCTCCAGTTTCAATCCTCCA<br>TGACGAGAAGCGCGTTTCCCGCCGCTGACCGCCGCCTCCTCAGACGGCCC<br>AAAGAGCTGCACAATCGATATTTCTTATTTGCACAATCGATGGTGCAAAGAC<br>TATCCTGCTGACTTAATCTTTCGTCAAGCGCAAAACGGCTCTAAGAGATTCG<br>AGGGGGCTCGGAGCAGCCATGGGTACAAAGAGGAGAAATACTAGATGGTT<br>TCTAAAGGTGAAGAAGATAACATGGCTATCATCAAAGAATTTATGCGTTTCA<br>AAGTTCACATGGAAGGTTCTGTTAACGGTCACGAATTTGAAATCGAAGGTG<br>AAGGTGAAGGTCGTCCGTACGAAGGTACCCAGACCGCTAAACTGAAAGTTA<br>CCAAAGGTGGTCCGCTGCCGTTCGCTTGGGACATCCTGTCTCCGCAGTTCA<br>TGTACGGTTCTAAAGCGTACGTTAAACACCCGGCTGACATCCCGGACTACC<br>TGAAACTGTCTTTCCCGGAAGGTTTCAAATGGGAACGTGTTATGAACTTCGA<br>AGATGGTGGTGTTGTTACCGTTACCCAGGACTCTTCTCTGCAAGACGGTGA<br>ATTTATCTACAAAGTTAAACTGCGTGGTACCAACTTCCCGTCTGACGGTCCG<br>GTTATGCAGAAAAAAACTATGGGTTGGGAAGCGAGCTCTGAACGTATGTAC<br>CCGGAAGATGGTGCTCTGAAAGGTGAAATCAAACAGCGTCTGAAACTGAAA<br>GACGGTGGTCACTACGACGCTGAAGTTAAAACCACCTACAAAGCTAAAAAA<br>CCGGTTCAGCTGCCGGGTGCTTACAACGTTAACATCAAACTGGACATCACC<br>TCTCACAACGAAGATTACACCATCGTTGAACAGTACGAACGTGCTGAAGGT

	CGTCACTCTACCGGTGGTATGGACGAACTGTACAAATAATCCAGGCATCAA<br>ATAAAACGAAAGGCTCAGTCGAAAGACTGGGCCTTTCGTTTTATCTGTTGTT<br>TGTCGGTGAACGCTCTCTACTAGAGTCACACTGGCTCACCTTCGGGTGGGC<br>CTTTCTGCGTTTATATGTTTACTAGTAGCGGCCGCTGCAG

Sequence name	**Psicose biosensor based on pPsiA promoter from *Sinorhizobium meliloti* and the PsiR transcription factor from *Sinorhizobium meliloti* with mCherry as reporter gene**
Acc. number	iGEM Parts Registry: BBa_K2448030<br>http://parts.igem.org/Part:BBa_K2448030
Sequence	TCCGGCAAAAAAGGGCAAGGTGTCACCACCCTGCCCTTTTTCTTTAAAACC<br>GAAAAGATTACTTCGCGTTATGCAGGCTTCCTCGCTCACTGACTCGCTGCG<br>CTCGGTCGTTCGGCTGCGGCGAGCGGTATCAGCTCACTCAAAGGCGGTAA<br>TACGGTTATCCACAGAATCAGGGGATAACGCAGGAAAGAACATGTGAGCAA<br>AAGGCCAGCAAAAGGCCAGGAACCGTAAAAAGGCCGCGTTGCTGGCGTTT<br>TTCCACAGGCTCCGCCCCCCTGACGAGCATCACAAAAATCGACGCTCAAGT<br>CAGAGGTGGCGAAACCCGACAGGACTATAAAGATACCAGGCGTTTCCCCC<br>TGGAAGCTCCCTCGTGCGCTCTCCTGTTCCGACCCTGCCGCTTACCGGATA<br>CCTGTCCGCCTTTCTCCCTTCGGGAAGCGTGGCGCTTTCTCATAGCTCACG<br>CTGTAGGTATCTCAGTTCGGTGTAGGTCGTTCGCTCCAAGCTGGGCTGTGT<br>GCACGAACCCCCCGTTCAGCCCGACCGCTGCGCCTTATCCGGTAACTATC<br>GTCTTGAGTCCAACCCGGTAAGACACGACTTATCGCCACTGGCAGCAGCCA<br>CTGGTAACAGGATTAGCAGAGCGAGGTATGTAGGCGGTGCTACAGAGTTCT<br>TGAAGTGGTGGCCTAACTACGGCTACACTAGAAGAACAGTATTTGGTATCT<br>GCGCTCTGCTGAAGCCAGTTACCTTCGGAAAAAGAGTTGGTAGCTCTTGAT<br>CCGGCAAACAAACCACCGCTGGTAGCGGTGGTTTTTTTGTTTGCAAGCAGC<br>AGATTACGCGCAGAAAAAAAGGATCTCAAGAAGATCCTTTGATCTTTTCTAC<br>GGGGTCTGACGCTCAGTGGAACGAAAACTCACGTTAAGGGATTTTGGTCAT<br>GAGATTATCAAAAAGGATCTTCACCTAGATCCTTTTAAATTAAAAATGAAGTT<br>TTAAATCAATCTAAAGTATATATGAGTAAACTTGGTCTGACAGCTCGAGGCT<br>TGGATTCTCACCAATAAAAAACGCCCGGCGGCAACCGAGCGTTCTGAACAA<br>ATCCAGATGGAGTTCTGAGGTCATTACTGGATCTATCAACAGGAGTCCAAG<br>CGAGCTCGATATCAAATTACGCCCCGCCCTGCCACTCATCGCAGTACTGTT<br>GTAATTCATTAAGCATTCTGCCGACATGGAAGCCATCACAAACGGCATGAT<br>GAACCTGAATCGCCAGCGGCATCAGCACCTTGTCGCCTTGCGTATAATATT<br>TGCCCATGGTGAAAACGGGGGCGAAGAAGTTGTCCATATTGGCCACGTTTA<br>AATCAAAACTGGTGAAACTCACCCAGGGATTGGCTGACACGAAAAACATAT<br>TCTCAATAAACCCTTTAGGGAAATAGGCCAGGTTTTCACCGTAACACGCCA<br>CATCTTGCGAATATATGTGTAGAAACTGCCGGAAATCGTCGTGGTATTCACT<br>CCAGAGCGATGAAAACGTTTCAGTTTGCTCATGGAAAACGGTGTAACAAGG<br>GTGAACACTATCCCATATCACCAGCTCACCGTCTTTCATTGCCATACGAAAT<br>TCCGGATGAGCATTCATCAGGCGGGCAAGAATGTGAATAAAGGCCGGATAA<br>AACTTGTGCTTATTTTTCTTTACGGTCTTTAAAAAGGCCGTAATATCCAGCTG<br>AACGGTCTGGTTATAGGTACATTGAGCAACTGACTGAAATGCCTCAAAATGT<br>TCTTTACGATGCCATTGGGATATATCAACGGTGGTATATCCAGTGATTTTTTT<br>CTCCATTTTAGCTTCCTTAGCTCCTGAAAATCTCGATAACTCAAAAAATACG<br>CCCGGTAGTGATCTTATTTCATTATGGTGAAAGTTGGAACCTCTTACGTGCC<br>CGATCAACTCGAGTGCCACCTGACGTCTAAGAAACCATTATTATCATGACAT<br>TAACCTATAAAAATAGGCGTATCACGAGGCAGAATTTCAGATAAAAAAAATC<br>CTTAGCTTTCGCTAAGGATGATTTCTGGAATTCGCGGCCGCTTCTAGAGGA<br>GCTGTTGACAATTAATCATCGGCTCGTATAATGTGTGGAATTGTGAGCGGAT<br>AACAATTCTCGAGTGGAAGAGACGGTACAAAGAGGAGAAATACCATATGAC<br>CAACGGTGGTCGTAAAAAAGCTACCATCTACGACCTGTCTGTTCTGTCTGG<br>TTCTTCTCCGTCTACCGTTTCTGCTGTTCTGAACGGTACCTGGCGTAAACGT<br>CGTATCAAAGAATCTACCGCTGAACTGATCCGTTCTCTGGCTGAAACCCAC<br>CAGTACACCGCTAACCGTCAGGCTCGTGGTCTGCGTTCTTCTCGTTCTGGT

	CTGGTTGGTCTGCTGCTGCCGGTTCACGACAACCGTTACTTCTCTTCTCTG<br>GCTCAGACCTTCGAAGCTCACGTTCGTTCTAAAGGTCAGTGCCCGATCGTT<br>GTTTCTGCTTCTCGTGACCCGGAAGAAGAACGTCGTACCGCTGAAACCCTG<br>ATCTCTTACTCTATCGACGAACTGTTCATCTGCGGTGCTACCGACCCGGAC<br>GGTGTTCACGAAGTTTGCGAAGCTGCTGGTCTGCGTCACATCAACATCGAC<br>CTGCCGGGTACCAAAGTTCCGTCTGTTATCTCTGACAACTTCGAAGGTGGT<br>CGTCTGCTGACCGAAGCTATCATCCGTCACTTCCCGGCTGAACGTCCGCTG<br>GAACCGGACGACCTGTACCTGTTCGGTGGTCGTGACGACCACGCTACCCG<br>TGAACGTATCCGTGGTTTCCGTGCTGTTAAATCTGACCTGCTGGGTGCTGA<br>CCCGGACGAATGCATCTGGCCGACCGGTTACGCTGCTGACAACGCTCGTA<br>AAGCGTTCGAAGCGTTCTACGAACAGCACGGTAAACTGCCGCGTGGTTTCT<br>TCGTTAACTCTTCTATCAACTTCGAAGGTCTGCTGCGTTTCATGGCTGAACA<br>CCCGCTGGAAAACTTCACCGACCTGGTTGTTGGTTGCTACGACTACGACCC<br>GTTCGCTTCTTTCCTGCCGTTCCCGGTTATCATGATCCGTCAGAACATCGAA<br>GGTATGATCGCTAAAGCGTTCGAAGTTATCGAAGAACCGCGTGCTTCTCTG<br>CAAATCCACATGATCGAACCGCAGCTGGTTCCGCCGCGTACCGCTCTGAC<br>CGGTCCGCTGGACGCTCTGATGGACTCTGAAATGCCGCGTGAATAAAGCA<br>GGCATGCCCAGGCATCAAATAAAACGAAAGGCTCAGTCGAAAGACTGGGC<br>CTTTCGTTTTATCTGTTGTTTGTCGGTGAACGCTCTCTACTAGAGTCACACT<br>GGCTCACCTTCGGGTGGGCCTTTCTGCGTTTATAACGTACGTACGTACGTG<br>GATCCCTTGGACGGTGCTTTCCTTGATCCGTCGCTTGCGCCACGTGCCGTT<br>TAGCACCGCACTGACGGTAGAGGGCGAACTTCCCGACAGCACCGAGAGAT<br>CATAGATCGTCGCTTTTTTCCTGCCGCCGTTCGTCATCTGACCTCCTCCAAA<br>CCCCGGAAAACCGATGCGCACGTTTCCTGGAATTGCTCTAGTGCCGATTTC<br>GGCTTGACGAAAGATTAAGTCTGAATGATAGTCATTGCACCATCGATTGTGC<br>AAAAAAGAAATATCGATTGTGCAAGTTGTTGGTGCCGTCTGAGGAGGCGGC<br>CGTCAGCGGCGGGATATCCCCTTCCGTGCAAAAGAATTAAGCTGGAGGCC<br>GGCGCGTGAAGCGCCCGGGAGCGTTCCCCTCGGGGAAACATGTGGAGGA<br>GAAACAGCAGCCATGGGTACAAAGAGGAGAAATACTAGATGGTTTCTAAAG<br>GTGAAGAAGATAACATGGCTATCATCAAAGAATTTATGCGTTTCAAAGTTCA<br>CATGGAAGGTTCTGTTAACGGTCACGAATTTGAAATCGAAGGTGAAGGTGA<br>AGGTCGTCCGTACGAAGGTACCCAGACCGCTAAACTGAAAGTTACCAAAGG<br>TGGTCCGCTGCCGTTCGCTTGGGACATCCTGTCTCCGCAGTTCATGTACGG<br>TTCTAAAGCGTACGTTAAACACCCGGCTGACATCCCGGACTACCTGAAACT<br>GTCTTTCCCGGAAGGTTTCAAATGGGAACGTGTTATGAACTTCGAAGATGG<br>TGGTGTTGTTACCGTTACCCAGGACTCTTCTCTGCAAGACGGTGAATTTATC<br>TACAAAGTTAAACTGCGTGGTACCAACTTCCCGTCTGACGGTCCGGTTATG<br>CAGAAAAAAACTATGGGTTGGGAAGCGAGCTCTGAACGTATGTACCCGGAA<br>GATGGTGCTCTGAAAGGTGAAATCAAACAGCGTCTGAAACTGAAAGACGGT<br>GGTCACTACGACGCTGAAGTTAAAACCACCTACAAAGCTAAAAAACCGGTT<br>CAGCTGCCGGGTGCTTACAACGTTAACATCAAACTGGACATCACCTCTCAC<br>AACGAAGATTACACCATCGTTGAACAGTACGAACGTGCTGAAGGTCGTCAC<br>TCTACCGGTGGTATGGACGAACTGTACAAATAATCCAGGCATCAAATAAAAC<br>GAAAGGCTCAGTCGAAAGACTGGGCCTTTCGTTTTATCTGTTGTTTGTCGGT<br>GAACGCTCTCTACTAGAGTCACACTGGCTCACCTTCGGGTGGGCCTTTCTG<br>CGTTTATATGTTTACTAGTAGCGGCCGCTGCAG

Sequence name	**Psicose biosensor based on pPsiR promoter from *Sinorhizobium meliloti* and the PsiR transcription factor from *Sinorhizobium meliloti* with mCherry as reporter gene**
Acc. number	iGEM Parts Registry: BBa_K2448031<br>http://parts.igem.org/Part:BBa_K2448031
Sequence	TCCGGCAAAAAAGGGCAAGGTGTCACCACCCTGCCCTTTTTCTTTAAAACC<br>GAAAAGATTACTTCGCGTTATGCAGGCTTCCTCGCTCACTGACTCGCTGCG<br>CTCGGTCGTTCGGCTGCGGCGAGCGGTATCAGCTCACTCAAAGGCGGTAA<br>TACGGTTATCCACAGAATCAGGGGATAACGCAGGAAAGAACATGTGAGCAA

	AAGGCCAGCAAAAGGCCAGGAACCGTAAAAAGGCCGCGTTGCTGGCGTTT<br>TTCCACAGGCTCCGCCCCCCTGACGAGCATCACAAAAATCGACGCTCAAGT<br>CAGAGGTGGCGAAACCCGACAGGACTATAAAGATACCAGGCGTTTCCCCC<br>TGGAAGCTCCCTCGTGCGCTCTCCTGTTCCGACCCTGCCGCTTACCGGATA<br>CCTGTCCGCCTTTCTCCCTTCGGGAAGCGTGGCGCTTTCTCATAGCTCACG<br>CTGTAGGTATCTCAGTTCGGTGTAGGTCGTTCGCTCCAAGCTGGGCTGTGT<br>GCACGAACCCCCCGTTCAGCCCGACCGCTGCGCCTTATCCGGTAACTATC<br>GTCTTGAGTCCAACCCGGTAAGACACGACTTATCGCCACTGGCAGCAGCCA<br>CTGGTAACAGGATTAGCAGAGCGAGGTATGTAGGCGGTGCTACAGAGTTCT<br>TGAAGTGGTGGCCTAACTACGGCTACACTAGAAGAACAGTATTTGGTATCT<br>GCGCTCTGCTGAAGCCAGTTACCTTCGGAAAAAGAGTTGGTAGCTCTTGAT<br>CCGGCAAACAAACCACCGCTGGTAGCGGTGGTTTTTTTGTTTGCAAGCAGC<br>AGATTACGCGCAGAAAAAAAGGATCTCAAGAAGATCCTTTGATCTTTTCTAC<br>GGGGTCTGACGCTCAGTGGAACGAAAACTCACGTTAAGGGATTTTGGTCAT<br>GAGATTATCAAAAAGGATCTTCACCTAGATCCTTTTAAATTAAAAATGAAGTT<br>TTAAATCAATCTAAAGTATATATGAGTAAACTTGGTCTGACAGCTCGAGGCT<br>TGGATTCTCACCAATAAAAAACGCCCGGCGGCAACCGAGCGTTCTGAACAA<br>ATCCAGATGGAGTTCTGAGGTCATTACTGGATCTATCAACAGGAGTCCAAG<br>CGAGCTCGATATCAAATTACGCCCCGCCCTGCCACTCATCGCAGTACTGTT<br>GTAATTCATTAAGCATTCTGCCGACATGGAAGCCATCACAAACGGCATGAT<br>GAACCTGAATCGCCAGCGGCATCAGCACCTTGTCGCCTTGCGTATAATATT<br>TGCCCATGGTGAAAACGGGGGCGAAGAAGTTGTCCATATTGGCCACGTTTA<br>AATCAAAACTGGTGAAACTCACCCAGGGATTGGCTGACACGAAAAACATAT<br>TCTCAATAAACCCTTTAGGGAAATAGGCCAGGTTTTCACCGTAACACGCCA<br>CATCTTGCGAATATATGTGTAGAAACTGCCGGAAATCGTCGTGGTATTCACT<br>CCAGAGCGATGAAAACGTTTCAGTTTGCTCATGGAAAACGGTGTAACAAGG<br>GTGAACACTATCCCATATCACCAGCTCACCGTCTTTCATTGCCATACGAAAT<br>TCCGGATGAGCATTCATCAGGCGGGCAAGAATGTGAATAAAGGCCGGATAA<br>AACTTGTGCTTATTTTTCTTTACGGTCTTTAAAAAGGCCGTAATATCCAGCTG<br>AACGGTCTGGTTATAGGTACATTGAGCAACTGACTGAAATGCCTCAAAATGT<br>TCTTTACGATGCCATTGGGATATATCAACGGTGGTATATCCAGTGATTTTTTT<br>CTCCATTTTAGCTTCCTTAGCTCCTGAAAATCTCGATAACTCAAAAAATACG<br>CCCGGTAGTGATCTTATTTCATTATGGTGAAAGTTGGAACCTCTTACGTGCC<br>CGATCAACTCGAGTGCCACCTGACGTCTAAGAAACCATTATTATCATGACAT<br>TAACCTATAAAAATAGGCGTATCACGAGGCAGAATTTCAGATAAAAAAAATC<br>CTTAGCTTTCGCTAAGGATGATTTCTGGAATTCGCGGCCGCTTCTAGAGGA<br>GCTGTTGACAATTAATCATCGGCTCGTATAATGTGTGGAATTGTGAGCGGAT<br>AACAATTCTCGAGTGGAAGAGACGGTACAAAGAGGAGAAATACCATATGAC<br>CAACGGTGGTCGTAAAAAAGCTACCATCTACGACCTGTCTGTTCTGTCTGG<br>TTCTTCTCCGTCTACCGTTTCTGCTGTTCTGAACGGTACCTGGCGTAAACGT<br>CGTATCAAAGAATCTACCGCTGAACTGATCCGTTCTCTGGCTGAAACCCAC<br>CAGTACACCGCTAACCGTCAGGCTCGTGGTCTGCGTTCTTCTCGTTCTGGT<br>CTGGTTGGTCTGCTGCTGCCGGTTCACGACAACCGTTACTTCTCTTCTCTG<br>GCTCAGACCTTCGAAGCTCACGTTCGTTCTAAAGGTCAGTGCCCGATCGTT<br>GTTTCTGCTTCTCGTGACCCGGAAGAAGAACGTCGTACCGCTGAAACCCTG<br>ATCTCTTACTCTATCGACGAACTGTTCATCTGCGGTGCTACCGACCCGGAC<br>GGTGTTCACGAAGTTTGCGAAGCTGCTGGTCTGCGTCACATCAACATCGAC<br>CTGCCGGGTACCAAAGTTCCGTCTGTTATCTCTGACAACTTCGAAGGTGGT<br>CGTCTGCTGACCGAAGCTATCATCCGTCACTTCCCGGCTGAACGTCCGCTG<br>GAACCGGACGACCTGTACCTGTTCGGTGGTCGTGACGACCACGCTACCCG<br>TGAACGTATCCGTGGTTTCCGTGCTGTTAAATCTGACCTGCTGGGTGCTGA<br>CCCGGACGAATGCATCTGGCCGACCGGTTACGCTGCTGACAACGCTCGTA<br>AAGCGTTCGAAGCGTTCTACGAACAGCACGGTAAACTGCCGCGTGGTTTCT<br>TCGTTAACTCTTCTATCAACTTCGAAGGTCTGCTGCGTTTCATGGCTGAACA<br>CCCGCTGGAAAACTTCACCGACCTGGTTGTTGGTTGCTACGACTACGACCC<br>GTTCGCTTCTTTCCTGCCGTTCCCGGTTATCATGATCCGTCAGAACATCGAA<br>GGTATGATCGCTAAAGCGTTCGAAGTTATCGAAGAACCGCGTGCTTCTCTG<br>CAAATCCACATGATCGAACCGCAGCTGGTTCCGCCGCGTACCGCTCTGAC<br>CGGTCCGCTGGACGCTCTGATGGACTCTGAAATGCCGCGTGAATAAAGCA

	GGCATGCCCAGGCATCAAATAAAACGAAAGGCTCAGTCGAAAGACTGGGC<br>CTTTCGTTTTATCTGTTGTTTGTCGGTGAACGCTCTCTACTAGAGTCACACT<br>GGCTCACCTTCGGGTGGGCCTTTCTGCGTTTATAACGTACGTACGTACGTG<br>GATCCCTTGGATGAACCACGGTATGCCGCCGATCTTGACGACGACACCGA<br>CCTTGCCCGTATCCTGCGCCGCGGCGGTATAGGCACCCGCAAGCGAAAGC<br>GACGCCGCCAGAGCGGCGGCAAGAATTTTCTTGATCATGTTTCTCCTCCAC<br>ATGTTTCCCCGAGGGGAACGCTCCCGGGCGCTTCACGCGCCGGCCTCCAG<br>CTTAATTCTTTTGCACGGAAGGGGATATCCCGCCGCTGACGGCCGCCTCCT<br>CAGACGGCACCAACAACTTGCACAATCGATATTTCTTTTTTGCACAATCGAT<br>GGTGCAATGACTATCATTCAGACTTAATCTTTCGTCAAGCCGAAATCGGCAC<br>TAGAGCAATTCCAGGAAACGTGCGCATCGGTTTTCCGGGGTTTGGAGGAG<br>GTCAGAGCAGCCATGGGTACAAAGAGGAGAAATACTAGATGGTTTCTAAAG<br>GTGAAGAAGATAACATGGCTATCATCAAAGAATTTATGCGTTTCAAAGTTCA<br>CATGGAAGGTTCTGTTAACGGTCACGAATTTGAAATCGAAGGTGAAGGTGA<br>AGGTCGTCCGTACGAAGGTACCCAGACCGCTAAACTGAAAGTTACCAAAGG<br>TGGTCCGCTGCCGTTCGCTTGGGACATCCTGTCTCCGCAGTTCATGTACGG<br>TTCTAAAGCGTACGTTAAACACCCGGCTGACATCCCGGACTACCTGAAACT<br>GTCTTTCCCGGAAGGTTTCAAATGGGAACGTGTTATGAACTTCGAAGATGG<br>TGGTGTTGTTACCGTTACCCAGGACTCTTCTCTGCAAGACGGTGAATTTATC<br>TACAAAGTTAAACTGCGTGGTACCAACTTCCCGTCTGACGGTCCGGTTATG<br>CAGAAAAAAACTATGGGTTGGGAAGCGAGCTCTGAACGTATGTACCCGGAA<br>GATGGTGCTCTGAAAGGTGAAATCAAACAGCGTCTGAAACTGAAAGACGGT<br>GGTCACTACGACGCTGAAGTTAAAACCACCTACAAAGCTAAAAAACCGGTT<br>CAGCTGCCGGGTGCTTACAACGTTAACATCAAACTGGACATCACCTCTCAC<br>AACGAAGATTACACCATCGTTGAACAGTACGAACGTGCTGAAGGTCGTCAC<br>TCTACCGGTGGTATGGACGAACTGTACAAATAATCCAGGCATCAAATAAAAC<br>GAAAGGCTCAGTCGAAAGACTGGGCCTTTCGTTTTATCTGTTGTTTGTCGGT<br>GAACGCTCTCTACTAGAGTCACACTGGCTCACCTTCGGGTGGGCCTTTCTG<br>CGTTTATATGTTTACTAGTAGCGGCCGCTGCAG

Sequence name	**Psicose biosensor based on pPsiA promoter from *Agrobacterium tumefaciens* and the PsiR transcription factor from *Agrobacterium tumefaciens* with mEmerald as reporter gene and a downstream the Mutant Drop Zone**
Acc. number	iGEM Parts Registry: BBa_K2448057<br>http://parts.igem.org/Part:BBa_K2448057
Sequence	TCCGGCAAAAAAGGGCAAGGTGTCACCACCCTGCCCTTTTTCTTTAAAACC<br>GAAAAGATTACTTCGCGTTATGCAGGCTTCCTCGCTCACTGACTCGCTGCG<br>CTCGGTCGTTCGGCTGCGGCGAGCGGTATCAGCTCACTCAAAGGCGGTAA<br>TACGGTTATCCACAGAATCAGGGGATAACGCAGGAAAGAACATGTGAGCAA<br>AAGGCCAGCAAAAGGCCAGGAACCGTAAAAAGGCCGCGTTGCTGGCGTTT<br>TTCCACAGGCTCCGCCCCCCTGACGAGCATCACAAAAATCGACGCTCAAGT<br>CAGAGGTGGCGAAACCCGACAGGACTATAAAGATACCAGGCGTTTCCCCC<br>TGGAAGCTCCCTCGTGCGCTCTCCTGTTCCGACCCTGCCGCTTACCGGATA<br>CCTGTCCGCCTTTCTCCCTTCGGGAAGCGTGGCGCTTTCTCATAGCTCACG<br>CTGTAGGTATCTCAGTTCGGTGTAGGTCGTTCGCTCCAAGCTGGGCTGTGT<br>GCACGAACCCCCCGTTCAGCCCGACCGCTGCGCCTTATCCGGTAACTATC<br>GTCTTGAGTCCAACCCGGTAAGACACGACTTATCGCCACTGGCAGCAGCCA<br>CTGGTAACAGGATTAGCAGAGCGAGGTATGTAGGCGGTGCTACAGAGTTCT<br>TGAAGTGGTGGCCTAACTACGGCTACACTAGAAGAACAGTATTTGGTATCT<br>GCGCTCTGCTGAAGCCAGTTACCTTCGGAAAAAGAGTTGGTAGCTCTTGAT<br>CCGGCAAACAAACCACCGCTGGTAGCGGTGGTTTTTTTGTTTGCAAGCAGC<br>AGATTACGCGCAGAAAAAAAGGATCTCAAGAAGATCCTTTGATCTTTTCTAC<br>GGGGTCTGACGCTCAGTGGAACGAAAACTCACGTTAAGGGATTTTGGTCAT<br>GAGATTATCAAAAAGGATCTTCACCTAGATCCTTTTAAATTAAAAATGAAGTT<br>TTAAATCAATCTAAAGTATATATGAGTAAACTTGGTCTGACAGCTCGAGGCT

	TGGATTCTCACCAATAAAAAACGCCCGGCGGCAACCGAGCGTTCTGAACAA<br>ATCCAGATGGAGTTCTGAGGTCATTACTGGATCTATCAACAGGAGTCCAAG<br>CGAGCTCGATATCAAATTACGCCCCGCCCTGCCACTCATCGCAGTACTGTT<br>GTAATTCATTAAGCATTCTGCCGACATGGAAGCCATCACAAACGGCATGAT<br>GAACCTGAATCGCCAGCGGCATCAGCACCTTGTCGCCTTGCGTATAATATT<br>TGCCCATGGTGAAAACGGGGGCGAAGAAGTTGTCCATATTGGCCACGTTTA<br>AATCAAAACTGGTGAAACTCACCCAGGGATTGGCTGACACGAAAAACATAT<br>TCTCAATAAACCCTTTAGGGAAATAGGCCAGGTTTTCACCGTAACACGCCA<br>CATCTTGCGAATATATGTGTAGAAACTGCCGGAAATCGTCGTGGTATTCACT<br>CCAGAGCGATGAAAACGTTTCAGTTTGCTCATGGAAAACGGTGTAACAAGG<br>GTGAACACTATCCCATATCACCAGCTCACCGTCTTTCATTGCCATACGAAAT<br>TCCGGATGAGCATTCATCAGGCGGGCAAGAATGTGAATAAAGGCCGGATAA<br>AACTTGTGCTTATTTTTCTTTACGGTCTTTAAAAAGGCCGTAATATCCAGCTG<br>AACGGTCTGGTTATAGGTACATTGAGCAACTGACTGAAATGCCTCAAAATGT<br>TCTTTACGATGCCATTGGGATATATCAACGGTGGTATATCCAGTGATTTTTTT<br>CTCCATTTTAGCTTCCTTAGCTCCTGAAAATCTCGATAACTCAAAAAATACG<br>CCCGGTAGTGATCTTATTTCATTATGGTGAAAGTTGGAACCTCTTACGTGCC<br>CGATCAACTCGAGTGCCACCTGACGTCTAAGAAACCATTATTATCATGACAT<br>TAACCTATAAAAATAGGCGTATCACGAGGCAGAATTTCAGATAAAAAAAATC<br>CTTAGCTTTCGCTAAGGATGATTTCTGGAATTCGCGGCCGCTTCTAGAGGA<br>GCTGTTGACAATTAATCATCGGCTCGTATAATGTGTGGAATTGTGAGCGGAT<br>AACAATTCTCGAGTGGAAGACTCGGTACAAAGAGGAGAAATACCATATGAC<br>CGGTATCTCTTCTAAAAAAGCTACCATCTACGACCTGTCTATCCTGTCTGGT<br>GCTTCTGCTTCTACCGTTTCTGCTGTTCTGAACGGTTCTTGGCGTAAACGTC<br>GTATCTCTGAAGAAACCGCTGACAAAATCCTGTCTCTGGCTAAAGCTCAGC<br>GTTACACCACCAACTTACAGGCTCGTGGTCTGCGTTCTTCTAAATCTGGTCT<br>GGTTGGTCTGCTGGTTCCGGTTTACGACAACCGTTTCTTCTCTTCTATGGCT<br>CAGACCTTCGAAGGTCAGGCTCGTAAACGTGGTCTGTCTCCGATGGTTGTT<br>TCTGGTCGTCGTGACCCGGAAGAAGAACGTCGTACCGTTGAAACCCTGATC<br>GCTTACTCTATCGACGCTCTGTTCATCGCTGGTGTTACCGACCCGGACGGT<br>GTTCACCAGGTTTGCGCTCGTGCTGCTCTGCCGCACGTTAACATCGACCTG<br>CCGGGTAAATTCGCTTCTTCTGTTATCTCTAACAACCGTCACGGTGCTGAAA<br>TCCTGACCGCTGCTATCCTGGCTCACGCTGCTAAAGGTGGTTCTCTGGGTC<br>CGGACGACGTTATCCTGTTCGGTGGTCACGACGACCACGCTTCTCGTGAAC<br>GTATCGACGGTTTCCACGCTGCTAAAGCTGACTACTTCGGTGTTGAAGGTG<br>GTGACGACATCGAAATCACCGGTTACTCTCCGCACATGACCGAAATGGCTT<br>TCGAACGTTTCTTCGGTCGTCGTGGTCGTCTGCCGCGTTGCTTCTTCGTTA<br>ACTCTTCTATCAACTTCGAAGGTCTGCTGCGTTTCATGGGTCGTCACGACG<br>GTGAAGCTTTCGGTGACATCGTTGTTGGTTGCTTCGACTACGACCCGTTCG<br>CTTCTTTCCTGCCGTTCCCGGTTTACATGATCAAACCGGACATCGCTCAGAT<br>GCTGGAAAAAGGTTTCGAACTGCTGGAAGAAAACCGTACCGAACCGGAAGT<br>TACCATCATCGAACCGCAGCTGATCCCGCCGCGTACCGCTCTGGAAGGTC<br>CGCTGGACGACATCTGGGACCCGGTTGCTCTGCGTCGTATGGCTAAATAAA<br>GCAGGCATGCCCAGGCATCAAATAAAACGAAAGGCTCAGTCGAAAGACTG<br>GGCCTTTCGTTTTATCTGTTGTTTGTCGGTGAACGCTCTCTACTAGAGTCAC<br>ACTGGCTCACCTTCGGGTGGGCCTTTCTGCGTTTATAACGTACGTACGTAC<br>GTGGATCCCTTGGAGTATAAATGGTGGCTTTTTTTGAACTTATGCCCGTCAC<br>TGTGATCTCCCCAACTGATTCCGATTATTAGAGCACGCATCCCCTTGACGG<br>AAGGGCGCTTCATGATATGGTTATTGCACCATCGATTGTGCAGATTGGCAA<br>TATCGATTGTGCATGGTGGTTGCTATGGGAGTGGCAAGGGAGAGTCTCGAA<br>TAAGCGAGATGAGAGATTTTGAACGCGTCCGGGAAAAACGGGCTGCGGGC<br>GGATTTCGTTTGCCGAATTTTTGAGGAGGAACATCAATGAAGAAAATTATTG<br>CTGCGGCGGTTGGTCTGTCGCTGGCGTTGCTCTCATCCGCAGCCTTTGCC<br>GAAGGGCCGAAGGTGGGCGTCGTCGTCAAGATCGGCGGCATTCCGTGGTT<br>CAACGCCAGCAGCCATGGGTACAAATGGAGGAAAAGAGGAGAAAAGATCA<br>ATGGTTTCTAAAGGTGAAGAACTGTTCACCGGTGTTGTTCCGATCCTGGTT<br>GAACTGGACGGTGACGTTAACGGTCACAAATTCTCTGTTTCTGGTGAAGGT<br>GAAGGTGACGCTACCTACGGTAAACTGACCCTGAAATTCATCTGCACCACC<br>GGTAAACTGCCGGTTCCGTGGCCGACCCTGGTTACCACCCTGACCTACGG

	TGTTCAGTGCTTCGCTCGTTACCCGGACCACATGAAACAGCACGACTTCTT CAAATCTGCTATGCCGGAAGGTTACGTTCAGGAACGTACCATCTTCTTCAAA GACGACGGTAACTACAAAACCCGTGCTGAAGTTAAATTCGAAGGTGACACC CTGGTTAACCGTATCGAACTGAAAGGTATCGACTTCAAAGAAGATGGTAAC ATCCTGGGTCACAAACTGGAATACAACTACAACTCTCACAAAGTTTACATCA CCGCTGACAAACAGAAAAACGGTATCAAAGTTAACTTCAAAACCCGTCACAA CATCGAAGATGGTTCTGTTCAGCTGGCTGACCACTACCAGCAGAACACCCC GATCGGTGACGGTCCGGTTCTGCTGCCGGACAACCACTACCTGTCTACCC AGTCTAAACTGTCTAAAGACCCGAACGAAAAACGTGACCACATGGTTCTGC TGGAATTTGTTACCGCTGCTGGTATCACCCTGGGTATGGACGAACTGTACA AATAAGAGAGCAGTTGGATAGCGTGACCGGCGCATCGGTCACGCTATTTGT TGAGGAGAGAGAGCTGTTGACAATTAATCATCGGCTCGTATAATGTGTGGA ATTGTGAGCGGATAACAATTGTACAAAGAGGAGAAACTCGAGGATGAGAGA CGGATCGATCCGTCTCAAGCGGCATGCCCAGGCATCAAATAAAACGAAAG GCTCAGTCGAAAGACTGGGCCTTTCGTTTTATCTGTTGTTTGTCGGTGAAC GCTCTCTACTAGAGTCACACTGGCTCACCTTCGGGTGGGCCTTTCTGCGTT TATAGCAGAACTAGTAGCGGCCGCTGCAG

Sequence name	**Psicose biosensor based on pPsiA promoter from *Agrobacterium tumefaciens* and the PsiR transcription factor from *Agrobacterium tumefaciens* with mEmerald as reporter gene and a downstream D-Psicose 3-epimerase (DPEase) from *Clostridium cellulolyticum* under the control of pTacI promoter**
Acc. number	iGEM Parts Registry: BBa_K2448058 http://parts.igem.org/Part:BBa_K2448058
Sequence	TCCGGCAAAAAAGGGCAAGGTGTCACCACCCTGCCCTTTTTCTTTAAAACC GAAAAGATTACTTCGCGTTATGCAGGCTTCCTCGCTCACTGACTCGCTGCG CTCGGTCGTTCGGCTGCGGCGAGCGGTATCAGCTCACTCAAAGGCGGTAA TACGGTTATCCACAGAATCAGGGGATAACGCAGGAAAGAACATGTGAGCAA AAGGCCAGCAAAAGGCCAGGAACCGTAAAAAGGCCGCGTTGCTGGCGTTT TTCCACAGGCTCCGCCCCCCTGACGAGCATCACAAAAATCGACGCTCAAGT CAGAGGTGGCGAAACCCGACAGGACTATAAAGATACCAGGCGTTTCCCCC TGGAAGCTCCCTCGTGCGCTCTCCTGTTCCGACCCTGCCGCTTACCGGATA CCTGTCCGCCTTTCTCCCTTCGGGAAGCGTGGCGCTTTCTCATAGCTCACG CTGTAGGTATCTCAGTTCGGTGTAGGTCGTTCGCTCCAAGCTGGGCTGTGT GCACGAACCCCCCGTTCAGCCCGACCGCTGCGCCTTATCCGGTAACTATC GTCTTGAGTCCAACCCGGTAAGACACGACTTATCGCCACTGGCAGCAGCCA CTGGTAACAGGATTAGCAGAGCGAGGTATGTAGGCGGTGCTACAGAGTTCT TGAAGTGGTGGCCTAACTACGGCTACACTAGAAGAACAGTATTTGGTATCT GCGCTCTGCTGAAGCCAGTTACCTTCGGAAAAAGAGTTGGTAGCTCTTGAT CCGGCAAACAAACCACCGCTGGTAGCGGTGGTTTTTTTGTTTGCAAGCAGC AGATTACGCGCAGAAAAAAAGGATCTCAAGAAGATCCTTTGATCTTTTCTAC GGGGTCTGACGCTCAGTGGAACGAAAACTCACGTTAAGGGATTTTGGTCAT GAGATTATCAAAAAGGATCTTCACCTAGATCCTTTTAAATTAAAAATGAAGTT TTAAATCAATCTAAAGTATATATGAGTAAACTTGGTCTGACAGCTCGAGGCT TGGATTCTCACCAATAAAAAACGCCCGGCGGCAACCGAGCGTTCTGAACAA ATCCAGATGGAGTTCTGAGGTCATTACTGGATCTATCAACAGGAGTCCAAG CGAGCTCGATATCAAATTACGCCCCGCCCTGCCACTCATCGCAGTACTGTT GTAATTCATTAAGCATTCTGCCGACATGGAAGCCATCACAAACGGCATGAT GAACCTGAATCGCCAGCGGCATCAGCACCTTGTCGCCTTGCGTATAATATT TGCCCATGGTGAAAACGGGGGCGAAGAAGTTGTCCATATTGGCCACGTTTA AATCAAAACTGGTGAAACTCACCCAGGGATTGGCTGACACGAAAAACATAT TCTCAATAAACCCTTTAGGGAAATAGGCCAGGTTTTCACCGTAACACGCCA CATCTTGCGAATATATGTGTAGAAACTGCCGGAAATCGTCGTGGTATTCACT CCAGAGCGATGAAAACGTTTCAGTTTGCTCATGGAAAACGGTGTAACAAGG GTGAACACTATCCCATATCACCAGCTCACCGTCTTTCATTGCCATACGAAAT

	TCCGGATGAGCATTCATCAGGCGGGCAAGAATGTGAATAAAGGCCGGATAA<br>AACTTGTGCTTATTTTTCTTTACGGTCTTTAAAAAGGCCGTAATATCCAGCTG<br>AACGGTCTGGTTATAGGTACATTGAGCAACTGACTGAAATGCCTCAAAATGT<br>TCTTTACGATGCCATTGGGATATATCAACGGTGGTATATCCAGTGATTTTTTT<br>CTCCATTTTAGCTTCCTTAGCTCCTGAAAATCTCGATAACTCAAAAAATACG<br>CCCGGTAGTGATCTTATTTCATTATGGTGAAAGTTGGAACCTCTTACGTGCC<br>CGATCAACTCGAGTGCCACCTGACGTCTAAGAAACCATTATTATCATGACAT<br>TAACCTATAAAAATAGGCGTATCACGAGGCAGAATTTCAGATAAAAAAAATC<br>CTTAGCTTTCGCTAAGGATGATTTCTGGAATTCGCGGCCGCTTCTAGAGGA<br>GCTGTTGACAATTAATCATCGGCTCGTATAATGTGTGGAATTGTGAGCGGAT<br>AACAATTCTCGAGTGGAAGACTCGGTACAAAGAGGAGAAATACCATATGAC<br>CGGTATCTCTTCTAAAAAAGCTACCATCTACGACCTGTCTATCCTGTCTGGT<br>GCTTCTGCTTCTACCGTTTCTGCTGTTCTGAACGGTTCTTGGCGTAAACGTC<br>GTATCTCTGAAGAAACCGCTGACAAAATCCTGTCTCTGGCTAAAGCTCAGC<br>GTTACACCACCAACTTACAGGCTCGTGGTCTGCGTTCTTCTAAATCTGGTCT<br>GGTTGGTCTGCTGGTTCCGGTTTACGACAACCGTTTCTTCTCTTCTATGGCT<br>CAGACCTTCGAAGGTCAGGCTCGTAAACGTGGTCTGTCTCCGATGGTTGTT<br>TCTGGTCGTCGTGACCCGGAAGAAGAACGTCGTACCGTTGAAACCCTGATC<br>GCTTACTCTATCGACGCTCTGTTCATCGCTGGTGTTACCGACCCGGACGGT<br>GTTCACCAGGTTTGCGCTCGTGCTGCTCTGCCGCACGTTAACATCGACCTG<br>CCGGGTAAATTCGCTTCTTCTGTTATCTCTAACAACCGTCACGGTGCTGAAA<br>TCCTGACCGCTGCTATCCTGGCTCACGCTGCTAAAGGTGGTTCTCTGGGTC<br>CGGACGACGTTATCCTGTTCGGTGGTCACGACGACCACGCTTCTCGTGAAC<br>GTATCGACGGTTTCCACGCTGCTAAAGCTGACTACTTCGGTGTTGAAGGTG<br>GTGACGACATCGAAATCACCGGTTACTCTCCGCACATGACCGAAATGGCTT<br>TCGAACGTTTCTTCGGTCGTCGTGGTCGTCTGCCGCGTTGCTTCTTCGTTA<br>ACTCTTCTATCAACTTCGAAGGTCTGCTGCGTTTCATGGGTCGTCACGACG<br>GTGAAGCTTTCGGTGACATCGTTGTTGGTTGCTTCGACTACGACCCGTTCG<br>CTTCTTTCCTGCCGTTCCCGGTTTACATGATCAAACCGGACATCGCTCAGAT<br>GCTGGAAAAAGGTTTCGAACTGCTGGAAGAAAACCGTACCGAACCGGAAGT<br>TACCATCATCGAACCGCAGCTGATCCCGCCGCGTACCGCTCTGGAAGGTC<br>CGCTGGACGACATCTGGGACCCGGTTGCTCTGCGTCGTATGGCTAAATAAA<br>GCAGGCATGCCCAGGCATCAAATAAAACGAAAGGCTCAGTCGAAAGACTG<br>GGCCTTTCGTTTTATCTGTTGTTTGTCGGTGAACGCTCTCTACTAGAGTCAC<br>ACTGGCTCACCTTCGGGTGGGCCTTTCTGCGTTTATAACGTACGTACGTAC<br>GTGGATCCCTTGGAGTATAAATGGTGGCTTTTTTTGAACTTATGCCCGTCAC<br>TGTGATCTCCCCAACTGATTCCGATTATTAGAGCACGCATCCCCTTGACGG<br>AAGGGCGCTTCATGATATGGTTATTGCACCATCGATTGTGCAGATTGGCAA<br>TATCGATTGTGCATGGTGGTTGCTATGGGAGTGGCAAGGGAGAGTCTCGAA<br>TAAGCGAGATGAGAGATTTTGAACGCGTCCGGGAAAAACGGGCTGCGGGC<br>GGATTTCGTTTGCCGAATTTTTGAGGAGGAACATCAATGAAGAAAATTATTG<br>CTGCGGCGGTTGGTCTGTCGCTGGCGTTGCTCTCATCCGCAGCCTTTGCC<br>GAAGGGCCGAAGGTGGGCGTCGTCGTCAAGATCGGCGGCATTCCGTGGTT<br>CAACGCCAGCAGCCATGGGTACAAATGGAGGAAAAGAGGAGAAAAGATCA<br>ATGGTTTCTAAAGGTGAAGAACTGTTCACCGGTGTTGTTCCGATCCTGGTT<br>GAACTGGACGGTGACGTTAACGGTCACAAATTCTCTGTTTCTGGTGAAGGT<br>GAAGGTGACGCTACCTACGGTAAACTGACCCTGAAATTCATCTGCACCACC<br>GGTAAACTGCCGGTTCCGTGGCCGACCCTGGTTACCACCCTGACCTACGG<br>TGTTCAGTGCTTCGCTCGTTACCCGGACCACATGAAACAGCACGACTTCTT<br>CAAATCTGCTATGCCGGAAGGTTACGTTCAGGAACGTACCATCTTCTTCAAA<br>GACGACGGTAACTACAAAACCCGTGCTGAAGTTAAATTCGAAGGTGACACC<br>CTGGTTAACCGTATCGAACTGAAAGGTATCGACTTCAAAGAAGATGGTAAC<br>ATCCTGGGTCACAAACTGGAATACAACTACAACTCTCACAAAGTTTACATCA<br>CCGCTGACAAACAGAAAAACGGTATCAAAGTTAACTTCAAAACCCGTCACAA<br>CATCGAAGATGGTTCTGTTCAGCTGGCTGACCACTACCAGCAGAACACCCC<br>GATCGGTGACGGTCCGGTTCTGCTGCCGGACAACCACTACCTGTCTACCC<br>AGTCTAAACTGTCTAAAGACCCGAACGAAAAACGTGACCACATGGTTCTGC<br>TGGAATTTGTTACCGCTGCTGGTATCACCCTGGGTATGGACGAACTGTACA<br>AATAAGAGAGCAGTTGGATAGCGTGACCGGCGCATCGGTCACGCTATTTGT

	TGAGGAGAGAGAGCTGTTGACAATTAATCATCGGCTCGTATAATGTGTGGA ATTGTGAGCGGATAACAATTGTACAAAGAGGAGAAACTCGAGGATGAAACA CGGTATCTACTACGCTTACTGGGAACAGGAATGGGAAGCTGACTACAAATA CTACATCGAAAAAGTTGCTAAACTGGGTTTCGACATCCTGGAAATCGCTGCT TCTCCGCTGCCGTTCTACTCTGACATCCAGATCAACGAACTGAAAGCTTGC GCTCACGGTAACGGTATCACCCTGACCGTTGGTCACGGTCCGTCTGCTGAA CAGAACCTGTCTTCTCCGGACCCGGACATCCGTAAAAACGCTAAAGCTTTC TACACCGACCTGCTGAAACGTCTGTACAAACTGGACGTTCACCTGATCGGT GGTGCTCTGTACTCTTACTGGCCGATCGACTACACCAAAACCATCGACAAA AAAGGTGACTGGGAACGTTCTGTTGAATCTGTTCGTGAAGTTGCTAAAGTT GCTGAAGCTTGCGGTGTTGACTTCTGCCTGGAAGTTCTGAACCGTTTCGAA AACTACCTGATCAACACCGCTCAGGAAGGTGTTGACTTCGTTAAACAGGTT GACCACAACAACGTTAAAGTTATGCTGGACACCTTCCACATGAACATCGAA GAAGACTCTATCGGTGGTGCTATCCGTACCGCTGGTTCTTACCTGGGTCAC CTGCACACCGGTGAATGCAACCGTAAAGTTCCGGGTCGTGGTCGTATCCC GTGGGTTGAAATCGGTGAAGCTCTGGCTGACATCGGTTACAACGGTTCTGT TGTTATGGAACCGTTCGTTCGTATGGGTGGTACCGTTGGTTCTAACATCAAA GTTTGGCGTGACATCTCTAACGGTGCTGACGAAAAAATGCTGGACCGTGAA GCTCAGGCTGCTCTGGACTTCTCTCGTTACGTTCTGGAATGCCACAAACAC TCTTAAAGCGGCATGCCCAGGCATCAAATAAAACGAAAGGCTCAGTCGAAA GACTGGGCCTTTCGTTTTATCTGTTGTTTGTCGGTGAACGCTCTCTACTAGA GTCACACTGGCTCACCTTCGGGTGGGCCTTTCTGCGTTTATAGCAGAACTA GTAGCGGCCGCTGCAG

Sequence name	**D-Psicose 3-epimerase (DPEase) from *Clostridium cellulolyticum* under the control of pTacI promoter**
Acc. number	iGEM Parts Registry: BBa_K2448033 http://parts.igem.org/Part:BBa_K2448033
Sequence	TCCGGCAAAAAAGGGCAAGGTGTCACCACCCTGCCCTTTTTCTTTAAAACC GAAAAGATTACTTCGCGTTATGCAGGCTTCCTCGCTCACTGACTCGCTGCG CTCGGTCGTTCGGCTGCGGCGAGCGGTATCAGCTCACTCAAAGGCGGTAA TACGGTTATCCACAGAATCAGGGGATAACGCAGGAAAGAACATGTGAGCAA AAGGCCAGCAAAAGGCCAGGAACCGTAAAAAGGCCGCGTTGCTGGCGTTT TTCCACAGGCTCCGCCCCCCTGACGAGCATCACAAAAATCGACGCTCAAGT CAGAGGTGGCGAAACCCGACAGGACTATAAAGATACCAGGCGTTTCCCCC TGGAAGCTCCCTCGTGCGCTCTCCTGTTCCGACCCTGCCGCTTACCGGATA CCTGTCCGCCTTTCTCCCTTCGGGAAGCGTGGCGCTTTCTCATAGCTCACG CTGTAGGTATCTCAGTTCGGTGTAGGTCGTTCGCTCCAAGCTGGGCTGTGT GCACGAACCCCCCGTTCAGCCCGACCGCTGCGCCTTATCCGGTAACTATC GTCTTGAGTCCAACCCGGTAAGACACGACTTATCGCCACTGGCAGCAGCCA CTGGTAACAGGATTAGCAGAGCGAGGTATGTAGGCGGTGCTACAGAGTTCT TGAAGTGGTGGCCTAACTACGGCTACACTAGAAGAACAGTATTTGGTATCT GCGCTCTGCTGAAGCCAGTTACCTTCGGAAAAAGAGTTGGTAGCTCTTGAT CCGGCAAACAAACCACCGCTGGTAGCGGTGGTTTTTTTGTTTGCAAGCAGC AGATTACGCGCAGAAAAAAAGGATCTCAAGAAGATCCTTTGATCTTTTCTAC GGGGTCTGACGCTCAGTGGAACGAAAACTCACGTTAAGGGATTTTGGTCAT GAGATTATCAAAAAGGATCTTCACCTAGATCCTTTTAAATTAAAAATGAAGTT TTAAATCAATCTAAAGTATATATGAGTAAACTTGGTCTGACAGCTCGAGGCT TGGATTCTCACCAATAAAAAACGCCCGGCGGCAACCGAGCGTTCTGAACAA ATCCAGATGGAGTTCTGAGGTCATTACTGGATCTATCAACAGGAGTCCAAG CGAGCTCGATATCAAATTACGCCCCGCCCTGCCACTCATCGCAGTACTGTT GTAATTCATTAAGCATTCTGCCGACATGGAAGCCATCACAAACGGCATGAT GAACCTGAATCGCCAGCGGCATCAGCACCTTGTCGCCTTGCGTATAATATT TGCCCATGGTGAAAACGGGGGCGAAGAAGTTGTCCATATTGGCCACGTTTA AATCAAAACTGGTGAAACTCACCCAGGGATTGGCTGACACGAAAAACATAT TCTCAATAAACCCTTTAGGGAAATAGGCCAGGTTTTCACCGTAACACGCCA

	CATCTTGCGAATATATGTGTAGAAACTGCCGGAAATCGTCGTGGTATTCACT<br>CCAGAGCGATGAAAACGTTTCAGTTTGCTCATGGAAAACGGTGTAACAAGG<br>GTGAACACTATCCCATATCACCAGCTCACCGTCTTTCATTGCCATACGAAAT<br>TCCGGATGAGCATTCATCAGGCGGGCAAGAATGTGAATAAAGGCCGGATAA<br>AACTTGTGCTTATTTTTCTTTACGGTCTTTAAAAAGGCCGTAATATCCAGCTG<br>AACGGTCTGGTTATAGGTACATTGAGCAACTGACTGAAATGCCTCAAAATGT<br>TCTTTACGATGCCATTGGGATATATCAACGGTGGTATATCCAGTGATTTTTTT<br>CTCCATTTTAGCTTCCTTAGCTCCTGAAAATCTCGATAACTCAAAAAATACG<br>CCCGGTAGTGATCTTATTTCATTATGGTGAAAGTTGGAACCTCTTACGTGCC<br>CGATCAACTCGAGTGCCACCTGACGTCTAAGAAACCATTATTATCATGACAT<br>TAACCTATAAAAATAGGCGTATCACGAGGCAGAATTTCAGATAAAAAAAATC<br>CTTAGCTTTCGCTAAGGATGATTTCTGGAATTCGCGGCCGCTTCTAGAGAG<br>GAGCTGTTGACAATTAATCATCGGCTCGTATAATGTGTGGAATTGTGAGCG<br>GATAACAATTTTAACTTTAAGAAGGAGATATACAAATGAAACACGGTATCTA<br>CTACGCTTACTGGGAACAGGAATGGGAAGCTGACTACAAATACTACATCGA<br>AAAAGTTGCTAAACTGGGTTTCGACATCCTGGAAATCGCTGCTTCTCCGCT<br>GCCGTTCTACTCTGACATCCAGATCAACGAACTGAAAGCTTGCGCTCACGG<br>TAACGGTATCACCCTGACCGTTGGTCACGGTCCGTCTGCTGAACAGAACCT<br>GTCTTCTCCGGACCCGGACATCCGTAAAAACGCTAAAGCTTTCTACACCGA<br>CCTGCTGAAACGTCTGTACAAACTGGACGTTCACCTGATCGGTGGTGCTCT<br>GTACTCTTACTGGCCGATCGACTACACCAAAACCATCGACAAAAAAGGTGA<br>CTGGGAACGTTCTGTTGAATCTGTTCGTGAAGTTGCTAAAGTTGCTGAAGCT<br>TGCGGTGTTGACTTCTGCCTGGAAGTTCTGAACCGTTTCGAAAACTACCTG<br>ATCAACACCGCTCAGGAAGGTGTTGACTTCGTTAAACAGGTTGACCACAAC<br>AACGTTAAAGTTATGCTGGACACCTTCCACATGAACATCGAAGAAGACTCTA<br>TCGGTGGTGCTATCCGTACCGCTGGTTCTTACCTGGGTCACCTGCACACCG<br>GTGAATGCAACCGTAAAGTTCCGGGTCGTGGTCGTATCCCGTGGGTTGAAA<br>TCGGTGAAGCTCTGGCTGACATCGGTTACAACGGTTCTGTTGTTATGGAAC<br>CGTTCGTTCGTATGGGTGGTACCGTTGGTTCTAACATCAAAGTTTGGCGTG<br>ACATCTCTAACGGTGCTGACGAAAAAATGCTGGACCGTGAAGCTCAGGCTG<br>CTCTGGACTTCTCTCGTTACGTTCTGGAATGCCACAAACACTCTTAATACTA<br>GTAGCGGCCGCTGCAG

Sequence name	**D-Psicose 3-epimerase (DPEase) from *Clostridium cellulolyticum* with a C-terminal Histidine tag under the control of pTacI promoter**
Acc. number	iGEM Parts Registry: BBa_K2448054<br>http://parts.igem.org/Part:BBa_K2448054
Sequence	TCCGGCAAAAAGGGCAAGGTGTCACCACCCTGCCCTTTTTCTTTAAAACC<br>GAAAAGATTACTTCGCGTTATGCAGGCTTCCTCGCTCACTGACTCGCTGCG<br>CTCGGTCGTTCGGCTGCGGCGAGCGGTATCAGCTCACTCAAAGGCGGTAA<br>TACGGTTATCCACAGAATCAGGGGATAACGCAGGAAAGAACATGTGAGCAA<br>AAGGCCAGCAAAAGGCCAGGAACCGTAAAAAGGCCGCGTTGCTGGCGTTT<br>TTCCACAGGCTCCGCCCCCCTGACGAGCATCACAAAAATCGACGCTCAAGT<br>CAGAGGTGGCGAAACCCGACAGGACTATAAAGATACCAGGCGTTTCCCCC<br>TGGAAGCTCCCTCGTGCGCTCTCCTGTTCCGACCCTGCCGCTTACCGGATA<br>CCTGTCCGCCTTTCTCCCTTCGGGAAGCGTGGCGCTTTCTCATAGCTCACG<br>CTGTAGGTATCTCAGTTCGGTGTAGGTCGTTCGCTCCAAGCTGGGCTGTGT<br>GCACGAACCCCCCGTTCAGCCCGACCGCTGCGCCTTATCCGGTAACTATC<br>GTCTTGAGTCCAACCCGGTAAGACACGACTTATCGCCACTGGCAGCAGCCA<br>CTGGTAACAGGATTAGCAGAGCGAGGTATGTAGGCGGTGCTACAGAGTTCT<br>TGAAGTGGTGGCCTAACTACGGCTACACTAGAAGAACAGTATTTGGTATCT<br>GCGCTCTGCTGAAGCCAGTTACCTTCGGAAAAAGAGTTGGTAGCTCTTGAT<br>CCGGCAAACAAACCACCGCTGGTAGCGGTGGTTTTTTTGTTTGCAAGCAGC<br>AGATTACGCGCAGAAAAAAAGGATCTCAAGAAGATCCTTTGATCTTTTCTAC<br>GGGGTCTGACGCTCAGTGGAACGAAAACTCACGTTAAGGGATTTTGGTCAT<br>GAGATTATCAAAAAGGATCTTCACCTAGATCCTTTTAAATTAAAAATGAAGTT

	TTAAATCAATCTAAAGTATATATGAGTAAACTTGGTCTGACAGCTCGAGGCT<br>TGGATTCTCACCAATAAAAAACGCCCGGCGGCAACCGAGCGTTCTGAACAA<br>ATCCAGATGGAGTTCTGAGGTCATTACTGGATCTATCAACAGGAGTCCAAG<br>CGAGCTCGATATCAAATTACGCCCCGCCCTGCCACTCATCGCAGTACTGTT<br>GTAATTCATTAAGCATTCTGCCGACATGGAAGCCATCACAAACGGCATGAT<br>GAACCTGAATCGCCAGCGGCATCAGCACCTTGTCGCCTTGCGTATAATATT<br>TGCCCATGGTGAAAACGGGGGCGAAGAAGTTGTCCATATTGGCCACGTTTA<br>AATCAAAACTGGTGAAACTCACCCAGGGATTGGCTGACACGAAAAACATAT<br>TCTCAATAAACCCTTTAGGGAAATAGGCCAGGTTTTCACCGTAACACGCCA<br>CATCTTGCGAATATATGTGTAGAAACTGCCGGAAATCGTCGTGGTATTCACT<br>CCAGAGCGATGAAAACGTTTCAGTTTGCTCATGGAAAACGGTGTAACAAGG<br>GTGAACACTATCCCATATCACCAGCTCACCGTCTTTCATTGCCATACGAAAT<br>TCCGGATGAGCATTCATCAGGCGGGCAAGAATGTGAATAAAGGCCGGATAA<br>AACTTGTGCTTATTTTTCTTTACGGTCTTTAAAAAGGCCGTAATATCCAGCTG<br>AACGGTCTGGTTATAGGTACATTGAGCAACTGACTGAAATGCCTCAAAATGT<br>TCTTTACGATGCCATTGGGATATATCAACGGTGGTATATCCAGTGATTTTTTT<br>CTCCATTTTAGCTTCCTTAGCTCCTGAAAATCTCGATAACTCAAAAAATACG<br>CCCGGTAGTGATCTTATTTCATTATGGTGAAAGTTGGAACCTCTTACGTGCC<br>CGATCAACTCGAGTGCCACCTGACGTCTAAGAAACCATTATTATCATGACAT<br>TAACCTATAAAAATAGGCGTATCACGAGGCAGAATTTCAGATAAAAAAAATC<br>CTTAGCTTTCGCTAAGGATGATTTCTGGAATTCGCGGCCGCTTCTAGAGAG<br>GAGCTGTTGACAATTAATCATCGGCTCGTATAATGTGTGGAATTGTGAGCG<br>GATAACAATTTTAACTTTAAGAAGGAGATATACAAATGAAACACGGTATCTA<br>CTACGCTTACTGGGAACAGGAATGGGAAGCTGACTACAAATACTACATCGA<br>AAAAGTTGCTAAACTGGGTTTCGACATCCTGGAAATCGCTGCTTCTCCGCT<br>GCCGTTCTACTCTGACATCCAGATCAACGAACTGAAAGCTTGCGCTCACGG<br>TAACGGTATCACCCTGACCGTTGGTCACGGTCCGTCTGCTGAACAGAACCT<br>GTCTTCTCCGGACCCGGACATCCGTAAAAACGCTAAAGCTTTCTACACCGA<br>CCTGCTGAAACGTCTGTACAAACTGGACGTTCACCTGATCGGTGGTGCTCT<br>GTACTCTTACTGGCCGATCGACTACACCAAAACCATCGACAAAAAAGGTGA<br>CTGGGAACGTTCTGTTGAATCTGTTCGTGAAGTTGCTAAAGTTGCTGAAGCT<br>TGCGGTGTTGACTTCTGCCTGGAAGTTCTGAACCGTTTCGAAAACTACCTG<br>ATCAACACCGCTCAGGAAGGTGTTGACTTCGTTAAACAGGTTGACCACAAC<br>AACGTTAAAGTTATGCTGGACACCTTCCACATGAACATCGAAGAAGACTCTA<br>TCGGTGGTGCTATCCGTACCGCTGGTTCTTACCTGGGTCACCTGCACACCG<br>GTGAATGCAACCGTAAAGTTCCGGGTCGTGGTCGTATCCCGTGGGTTGAAA<br>TCGGTGAAGCTCTGGCTGACATCGGTTACAACGGTTCTGTTGTTATGGAAC<br>CGTTCGTTCGTATGGGTGGTACCGTTGGTTCTAACATCAAAGTTTGGCGTG<br>ACATCTCTAACGGTGCTGACGAAAAAATGCTGGACCGTGAAGCTCAGGCTG<br>CTCTGGACTTCTCTCGTTACGTTCTGGAATGCCACAAACACTCTCTCGAGCA<br>CCACCATCACCACCACTAATACTAGTAGCGGCCGCTGCAG

Supplementary materials and methods

Plasmid construction

Universal Biosensing Chassis (UBC) was constructed in 5 steps:
Step 1: The pSB1C3 backbone vector contains a BsmBI cloning site within the chloramphenicol resistance gene. Its presence prevents from using the Golden Gate assembly technique with this backbone. To circumvent this issue, we performed a site-directed mutagenesis and created the pSB1C3 BsmBI free backbone. The single synonymous mutation (G1385C) was introduced by the Single-Primer Reactions IN Parallel (SPRINP) site directed mutagenesis protocol (3) using the primers 5'-AGGGATTGGCTGACACGAAAAACAT-3' and 5'-ATGTTTTTCGTGTCAGCCAATCCCT-3'. The pSB1C3 BsmBI free backbone is available in the iGEM's Registry of Standard Biological Parts repository under the acc. number BBa_K2448036 (http://parts.igem.org/Part:BBa_K2448036).
Step 2: The pSB1C3 BsmBI free backbone was used as template in a PCR reaction with the primers 5'-GCGGTCTCTGCAGTCCGGCAAAAAAGGGCAAGG-3' and 5'-GCGGTCTCTTCCAGAAATCATCC TTAGCG-3' and the PCR product was assembled by Golden Gate with BsaI to a gBlock fragment 5'-GCTACGATCTGGTCTCATGGAATTCGCGGCCGCTTCTAGAGGAGCTGTTGACAATTAATCATCG GCTCGTATAATGTGTGGAATTGTGAGCGGATAACAATTCTCGAGTGGAAGAGACGGTACCATCG TCTCAGCAGGCATGCCCAGGCATCAAATAAAACGAAAGGCTCAGTCGAAAGACTGGGCCTTTCG TTTTATCTGTTGTTTGTCGGTGAACGCTCTCTACTAGAGTCACACTGGCTCACCTTCGGGTGGGC CTTTCTGCGTTTATAACGTACGTACGTACGTGGATCCCTTGGAAAGTCTTCACTGTTTGAAGACA GGCAGCCATGGGTACAAAGAGGAGAAATACTAGATGGTTTCTAAAGGTGAAGAAGATAACATGG CTATCATCAAAGAATTTATGCGTTTCAAAGTTCACATGGAAGGTTCTGTTAACGGTCACGAATTTG AAATCGAAGGTGAAGGTGAAGGTCGTCCGTACGAAGGTACCCAGACCGCTAAACTGAAAGTTAC CAAAGGTGGTCCGCTGCCGTTCGCTTGGGACATCCTGTCTCCGCAGTTCATGTACGGTTCTAAA GCGTACGTTAAACACCCGGCTGACATCCCGGACTACCTGAAACTGTCTTTCCCGGAAGGTTTCA AATGGGAACGTGTTATGAACTTCGAAGATGGTGGTGTTGTTACCGTTACCCAGGACTCTTCTCTG CAAGACGGTGAATTTATCTACAAAGTTAAACTGCGTGGTACCAACTTCCCGTCTGACGGTCCGGT TATGCAGAAAAAAACTATGGGTTGGGAAGCGAGCTCTGAACGTATGTACCCGGAAGATGGTGCT CTGAAAGGTGAAATCAAACAGCGTCTGAAACTGAAAGACGGTGGTCACTACGACGCTGAAGTTA AAACCACCTACAAAGCTAAAAAACCGGTTCAGCTGCCGGGTGCTTACAACGTTAACATCAAACTG GACATCACCTCTCACAACGAAGATTACACCATCGTTGAACAGTACGAACGTGCTGAAGGTCGTCA CTCTACCGGTGGTATGGACGAACTGTACAAATAATCCAGGCATCAAATAAAACGAAAGGCTCAGT CGAAAGACTGGGCCTTTCGTTTTATCTGTTGTTTGTCGGTGAACGCTCTCTACTAGAGTCACACT GGCTCACCTTCGGGTGGGCCTTTCTGCGTTTATATGTTTACTAGTAGCGGCCGCTGCAGTGAGA CCGCTACGATC-3'. The resulting plasmid, the Universal Biosensing Chassis (UBC) short version, is available in the iGEM's Registry of Standard Biological Parts repository under the acc. number BBa_K2448024 (http://parts.igem.org/Part:BBa_K2448024).
Step 3: The BBa_K2448024 was used as template in a PCR reaction with the primers 5'-GCGCGGT CTCAGCAGATCGTCTCAGCAGGCATGC-3' and 5'-GCGCGGTCTCATCCACTCTTCCACTCGAGAA TTG-3' and the PCR product was assembled by Golden Gate with BsaI to a gBlock fragment containing the mEmerald insertion marker 5'-GCGCGCGGTCTCATGGAGGAAAAGAGGAGAAA AGATCAATGGTTTCTAAAGGTGAAGAACTGTTCACCGGTGTTGTTCCGATCCTGGTTGAACTGGA CGGTGACGTTAACGGTCACAAATTCTCTGTTTCTGGTGAAGGTGAAGGTGACGCTACCTACGGT AAACTGACCCTGAAATTCATCTGCACCACCGGTAAACTGCCGGTTCCGTGGCCGACCCTGGTTA CCACCCTGACCTACGGTGTTCAGTGCTTCGCTCGTTACCCGGACCACATGAAACAGCACGACTT CTTCAAATCTGCTATGCCGGAAGGTTACGTTCAGGAACGTACCATCTTCTTCAAAGACGACGGTA ACTACAAAACCCGTGCTGAAGTTAAATTCGAAGGTGACACCCTGGTTAACCGTATCGAACTGAAA GGTATCGACTTCAAAGAAGATGGTAACATCCTGGGTCACAAACTGGAATACAACTACAACTCTCA CAAAGTTTACATCACCGCTGACAAACAGAAAAACGGTATCAAAGTTAACTTCAAAACCCGTCACA ACATCGAAGATGGTTCTGTTCAGCTGGCTGACCACTACCAGCAGAACACCCCGATCGGTGACGG TCCGGTTCTGCTGCCGGACAACCACTACCTGTCTACCCAGTCTAAACTGTCTAAAGACCCGAAC GAAAAACGTGACCACATGGTTCTGCTGGAATTTGTTACCGCTGCTGGTATCACCCTGGGTATGGA CGAACTGTACAAATAAGAGAGCAGTGAGACCGCGCGC-3'.
Step 4: Due to a design error, the insertion mEmerald in BBa_K2448024 (step 3) destroyed the BsmBI site between the pTacI promoter and mEmerald. This error was corrected by site directed mutagenesis following the Single-Primer Reactions IN Parallel (SPRINP) protocol (3) using the primers 5'-CGAGTGGAAGAGACGAGGAAAAGAGG-3' and 5'-CCTCTTTTCCTCGTCTCTTCCACT CG-3'.

Step 5: The plasmid obtained at step 4 was used as template in a PCR reaction with the primers 5'-GCGCGGTCTCAGCAGGGTACAAAGAGGAGAAATACTAGATGGTTTC-3' and 5'-GCGCGGTCTC ATCCAACGTACGTACGTACGTACGTTATAAACGCAG-3' and the PCR product was assembled by Golden Gate with BsaI to a gBlock fragment containing the LacZα insertion marker under the control of J23100 constitutive promoter 5'-GCGGTCTCATGGAGAGAGGATCCCTTGGAAAGTCTTCACTTG ACGGCTAGCTCAGTCCTAGGTACAGTGCTAGCAATTAAAGAGGAGAACAGCTATGACCATGATC ACCCCGAGCCTGCACGCTTGCCGTTCTACCCTGGAAGATCCGCGTGTTCCGAGCTCTAACTCTC TGGCTGTTGTTCTGCAACGTCGTGACTGGGAAAACCCGGGTGTTACCCAGCTGAACCGTCTGGC TGCTCACCCGCCGTTCGCTTCTTGGCGTAACTCTGAAGAAGCTCGTACCGACCGTCCGAGCCAG CAGCTGCGTTCTCTGAACGGTGAATGGCGTCTGATGCGTTACTTCCTGCTGACCCACCTGTGCG GTATCTCTCACCGTATCTGGTGCACCCTGTCTACCATCTGCTCTGACGCTGCTTAAGCCAGGCAT CAAATAAAACGAAAGGCTCAGTCGAAAGACTGGGCCTTTCGTTTTATCTGTTGTTTGTCGGTGAA CGCTCTCTACTAGAGTCACACTGGCTCACCTTCGGGTGGGCCTTTCTGCGTTTATATGAAGACAG GCAGCCATGGGAGAGCAGTGAGACCGC-3'. The resulting plasmid is the Universal Biosensing Chassis (UBC).

Psicose biosensor based on pPsiA promoter from *Agrobacterium tumefaciens* and the PsiR transcription factor from *Agrobacterium tumefaciens* with mCherry as reporter gene was constructed in 2 steps:

Step 1: A gBlock fragment containing the pPsiA promoter 5'-GCGGATCCCGAGAAGACAATGGAGTA TAAATGGTGGCTTTTTTTGAACTTATGCCCGTCACTGTGATCTCCCCAACTGATTCCGATTATTAG AGCACGCATCCCCTTGACGGAAGGGCGCTTCATGATATGGTTATTGCACCATCGATTGTGCAGA TTGGCAATATCGATTGTGCATGGTGGTTGCTATGGGAGTGGCAAGGGAGAGTCTCGAATAAGCG AGATGAGAGATTTTGAACGCGTCCGGGAAAAACGGGCTGCGGGCGGATTTCGTTTGCCGAATTT TTGAGGAGGAACATCAATGAAGAAAATTATTGCTGCGGCGGTTGGTCTGTCGCTGGCGTTGCTC TCATCCGCAGCCTTTGCCGAAGGGCCGAAGGTGGGCGTCGTCGTCAAGATCGGCGGCATTCCG TGGTTCAACGCCAGCAGAAGTCTTCACCATGGCATATGGC-3' was inserted by Golden Gate with BbsI into the Universal Biosensing Chassis.

Step 2: The plasmid obtained at step 1 was assembled by Golden Gate with BsmBI to a gBlock fragment containing the PsiR gene 5'-GCAGCGCCTCGAGCGTCTCATGGAAGAGACGGTAC AAAGAGGAGAAATACCATATGACCGGTATCTCTTCTAAAAAAGCTACCATCTACGACCTGTCTATC CTGTCTGGTGCTTCTGCTTCTACCGTTTCTGCTGTTCTGAACGGTTCTTGGCGTAAACGTCGTAT CTCTGAAGAAACCGCTGACAAAATCCTGTCTCTGGCTAAAGCTCAGCGTTACACCACCAACTTAC AGGCTCGTGGTCTGCGTTCTTCTAAATCTGGTCTGGTTGGTCTGCTGGTTCCGGTTTACGACAAC CGTTTCTTCTCTTCTATGGCTCAGACCTTCGAAGGTCAGGCTCGTAAACGTGGTCTGTCTCCGAT GGTTGTTTCTGGTCGTCGTGACCCGGAAGAAGAACGTCGTACCGTTGAAACCCTGATCGCTTAC TCTATCGACGCTCTGTTCATCGCTGGTGTTACCGACCCGGACGGTGTTCACCAGGTTTGCGCTC GTGCTGCTCTGCCGCACGTTAACATCGACCTGCCGGGTAAATTCGCTTCTTCTGTTATCTCTAAC AACCGTCACGGTGCTGAAATCCTGACCGCTGCTATCCTGGCTCACGCTGCTAAAGGTGGTTCTC TGGGTCCGGACGACGTTATCCTGTTCGGTGGTCACGACGACCACGCTTCTCGTGAACGTATCGA CGGTTTCCACGCTGCTAAAGCTGACTACTTCGGTGTTGAAGGTGGTGACGACATCGAAATCACC GGTTACTCTCCGCACATGACCGAAATGGCTTTCGAACGTTTCTTCGGTCGTCGTGGTCGTCTGC CGCGTTGCTTCTTCGTTAACTCTTCTATCAACTTCGAAGGTCTGCTGCGTTTCATGGGTCGTCAC GACGGTGAAGCTTTCGGTGACATCGTTGTTGGTTGCTTCGACTACGACCCGTTCGCTTCTTTCCT GCCGTTCCCGGTTTACATGATCAAACCGGACATCGCTCAGATGCTGGAAAAAGGTTTCGAACTG CTGGAAGAAAACCGTACCGAACCGGAAGTTACCATCATCGAACCGCAGCTGATCCCGCCGCGTA CCGCTCTGGAAGGTCCGCTGGACGACATCTGGGACCCGGTTGCTCTGCGTCGTATGGCTAAATA AAGCAGTGAGACGGCATGCGCGCGC-3'. The resulting plasmid is the psicose biosensor based on pPsiA promoter from *Agrobacterium tumefaciens* and the PsiR transcription factor from *Agrobacterium tumefaciens* with mCherry as reporter gene.

Psicose biosensor based on pPsiR promoter from *Agrobacterium tumefaciens* and the PsiR transcription factor from *Agrobacterium tumefaciens* with mCherry as reporter gene was constructed in 2 steps:

Step 1: The pPsiR was amplified by PCR using *Agrobacterium tumefaciens* str. C58 genomic DNA as template and the primers 5'-GCGGATCCCGAGAAGACAATGGAGGAGGCGTTGAACCACGGA ATG-3' and 5'-GCCATATGCCATGGTGAAGACTTCTGCTATGTGATCTCCCCAACTGATT-3' and then inserted by Golden Gate with BbsI into the Universal Biosensing Chassis.

Step 2: The plasmid obtained at step 1 was assembled by Golden Gate with BsmBI to a gBlock fragment containing the PsiR gene 5'-GCAGCGCCTCGAGCGTCTCATGGAAGAGACGGTAC AAAGAGGAGAAATACCATATGACCGGTATCTCTTCTAAAAAAGCTACCATCTACGACCTGTCTATC CTGTCTGGTGCTTCTGCTTCTACCGTTTCTGCTGTTCTGAACGGTTCTTGGCGTAAACGTCGTAT CTCTGAAGAAACCGCTGACAAAATCCTGTCTCTGGCTAAAGCTCAGCGTTACACCACCAACTTAC AGGCTCGTGGTCTGCGTTCTTCTAAATCTGGTCTGGTTGGTCTGCTGGTTCCGGTTTACGACAAC CGTTTCTTCTCTTCTATGGCTCAGACCTTCGAAGGTCAGGCTCGTAAACGTGGTCTGTCTCCGAT GGTTGTTTCTGGTCGTCGTGACCCGGAAGAAGAACGTCGTACCGTTGAAACCCTGATCGCTTAC TCTATCGACGCTCTGTTCATCGCTGGTGTTACCGACCCGGACGGTGTTCACCAGGTTTGCGCTC GTGCTGCTCTGCCGCACGTTAACATCGACCTGCCGGGTAAATTCGCTTCTTCTGTTATCTCTAAC AACCGTCACGGTGCTGAAATCCTGACCGCTGCTATCCTGGCTCACGCTGCTAAAGGTGGTTCTC TGGGTCCGGACGACGTTATCCTGTTCGGTGGTCACGACGACCACGCTTCTCGTGAACGTATCGA CGGTTTCCACGCTGCTAAAGCTGACTACTTCGGTGTTGAAGGTGGTGACGACATCGAAATCACC GGTTACTCTCCGCACATGACCGAAATGGCTTTCGAACGTTTCTTCGGTCGTCGTGGTCGTCTGC CGCGTTGCTTCTTCGTTAACTCTTCTATCAACTTCGAAGGTCTGCTGCGTTTCATGGGTCGTCAC GACGGTGAAGCTTTCGGTGACATCGTTGTTGGTTGCTTCGACTACGACCCGTTCGCTTCTTTCCT GCCGTTCCCGGTTTACATGATCAAACCGGACATCGCTCAGATGCTGGAAAAAGGTTTCGAACTG CTGGAAGAAAACCGTACCGAACCGGAAGTTACCATCATCGAACCGCAGCTGATCCCGCCGCGTA CCGCTCTGGAAGGTCCGCTGGACGACATCTGGGACCCGGTTGCTCTGCGTCGTATGGCTAAATA AAGCAGTGAGACGGCATGCGCGCGC-3'. The resulting plasmid is the psicose biosensor based on pPsiR promoter from *Agrobacterium tumefaciens* and the PsiR transcription factor from *Agrobacterium tumefaciens* with mCherry as reporter gene.

Psicose biosensor based on pPsiTacI synthetic promoter and the PsiR transcription factor from *Agrobacterium tumefaciens* with mCherry as reporter gene was constructed in 2 steps:
Step 1: A gBlock fragment containing the pPsiTacI promoter 5'-GCGGATCCCGAGAAGACAATGGAT GAGCTGTTGACAATTAATCATCGGCTCGTATAATGTGTGGATTGCACAATCGATGGTGCAAAGCA GAAGTCTTCACCATGGCATATGGC-3' was inserted by Golden Gate with BbsI into the Universal Biosensing Chassis.
Step 2: The plasmid obtained at step 1 was assembled by Golden Gate with BsmBI to a gBlock fragment containing the PsiR gene 5'-GCAGCGCCTCGAGCGTCTCATGGAAGAGACGGTAC AAAGAGGAGAAATACCATATGACCGGTATCTCTTCTAAAAAAGCTACCATCTACGACCTGTCTATC CTGTCTGGTGCTTCTGCTTCTACCGTTTCTGCTGTTCTGAACGGTTCTTGGCGTAAACGTCGTAT CTCTGAAGAAACCGCTGACAAAATCCTGTCTCTGGCTAAAGCTCAGCGTTACACCACCAACTTAC AGGCTCGTGGTCTGCGTTCTTCTAAATCTGGTCTGGTTGGTCTGCTGGTTCCGGTTTACGACAAC CGTTTCTTCTCTTCTATGGCTCAGACCTTCGAAGGTCAGGCTCGTAAACGTGGTCTGTCTCCGAT GGTTGTTTCTGGTCGTCGTGACCCGGAAGAAGAACGTCGTACCGTTGAAACCCTGATCGCTTAC TCTATCGACGCTCTGTTCATCGCTGGTGTTACCGACCCGGACGGTGTTCACCAGGTTTGCGCTC GTGCTGCTCTGCCGCACGTTAACATCGACCTGCCGGGTAAATTCGCTTCTTCTGTTATCTCTAAC AACCGTCACGGTGCTGAAATCCTGACCGCTGCTATCCTGGCTCACGCTGCTAAAGGTGGTTCTC TGGGTCCGGACGACGTTATCCTGTTCGGTGGTCACGACGACCACGCTTCTCGTGAACGTATCGA CGGTTTCCACGCTGCTAAAGCTGACTACTTCGGTGTTGAAGGTGGTGACGACATCGAAATCACC GGTTACTCTCCGCACATGACCGAAATGGCTTTCGAACGTTTCTTCGGTCGTCGTGGTCGTCTGC CGCGTTGCTTCTTCGTTAACTCTTCTATCAACTTCGAAGGTCTGCTGCGTTTCATGGGTCGTCAC GACGGTGAAGCTTTCGGTGACATCGTTGTTGGTTGCTTCGACTACGACCCGTTCGCTTCTTTCCT GCCGTTCCCGGTTTACATGATCAAACCGGACATCGCTCAGATGCTGGAAAAAGGTTTCGAACTG CTGGAAGAAAACCGTACCGAACCGGAAGTTACCATCATCGAACCGCAGCTGATCCCGCCGCGTA CCGCTCTGGAAGGTCCGCTGGACGACATCTGGGACCCGGTTGCTCTGCGTCGTATGGCTAAATA AAGCAGTGAGACGGCATGCGCGCGC-3'. The resulting plasmid is the psicose biosensor based on pPsiTacI synthetic promoter and the PsiR transcription factor from *Agrobacterium tumefaciens* with mCherry as reporter gene.

Psicose biosensor based on pPsiA promoter from *Sinorhizobium fredii* and the PsiR transcription factor from *Sinorhizobium fredii* with mCherry as reporter gene was constructed in 2 steps:
Step 1: A gBlock fragment containing the pPsiA promoter 5'-GCGGATCCCGAGAAGACAATGGAGG TGGGTCTGGGCGAGGTTGCGGATCAACTCGGCGGTGCTTTCCTTGATGCGCCGCTTGCGCCAG GTGCCGTTCAGCACGGCGCTGACCGTCGAGGGCGAGCTGCCGGAGAGCACCGAGAGATCGTA

GATCGTCGCCTTTTTCTTGCCGCTGTTCGCCATCCGAGCCCCCTCGAATCTCTTAGAGCCGTTTT GCGCTTGACGAAAGATTAAGTCTGCACGATAGTCTTTGCACCATCGATTGTGCAAATAAGAAATA TCGATTGTGCAGCTCTTTGGGCCGTCTGAGGAGGCGGCGGTCAGCGGCGGGAAACGCGCTTCT CGTCATGGAGGATTGAAACTGGAGGCCGGCGCGCCAGCGCCCGGGAGAGTTCCCGTTGCGGG AACCTGTGGAGGAGAGACAGCAGAAGTCTTCACCATGGCATATGGC-3' was inserted by Golden Gate with BbsI into the Universal Biosensing Chassis.

Step 2: The plasmid obtained at step 1 was assembled by Golden Gate with BsmBI to a gBlock fragment containing the PsiR gene 5'-GCAGCGCCTCGAGCGTCTCATGGAAGAGACGGTACAAAGA GGAGAAATACCATATGGCTAACTCTGGTAAAAAAAAAGCTACCATCTACGACCTGTCTGTTCTGT CTGGTTCTTCTCCGTCTACCGTTTCTGCTGTTCTGAACGGTACCTGGCGTAAACGTCGTATCAAA GAATCTACCGCTGAACTGATCCGTAACCTGGCTGAAACCCACCAGTACACCGCTAACCGTCAGG CTCGTGGTCTGCGTTCTTCTCGTTCTGGTCTGGTTGGTCTGCTGCTGCCGGTTCACGACAACCG TTACTTCTCTTCTCTGGCTCAGACCTTCGAAGCTCACGTTCGTTCTAAAGGTCAGTGCCCGATCG TTGTTTCTGCTTCTCGTGACCCGCAGGAAGAACGTAAAACCGCTGAAACCCTGATCTCTTACTCT ATCGACGAACTGTTCATCTGCGGTGCTACCGACCCGGACGGTGTTCACGAAGTTTGCGAAGCTG CTGGTCTGAAACACATCAACATCGACCTGCCGGGTACCAAAGTTCCGTCTGTTATCTCTGACAAC TTCGAAGGTGGTCGTCTGCTGACCGAAGCTATCATCCGTCACTTCCCGGCTGACCGTGCTCTGG CTCCGACCGACCTGTACCTGTTCGGTGGTCGTAACGACCACGCTTCTCACGAACGTATCCGTGG TTTCCGTGCTGTTAAAAAAGACCTGCTGGGTGACGACCCGGACGAATGCATCCAGCCGACCGGT TACGCTGCTAACAACGCTCGTAAAGCGTTCGAAGCGTTCTACGCTCGTCACGGTAAACTGCCGC GTGGTCTGTTCGTTAACTCTTCTATCAACTTCGAAGGTCTGCTGCGTTTCATGGCTGAACACCCG CACGACAACTTCACCGACCTGGTTGTTGGTTGCTACGACTACGACCCGTTCGCTTCTTTCCTGCC GTTCCCGGTTATCATGATCCGTCAGGACGTTGAAGGTATGATCGCTAAAGCGTTCGAAGTTATCG AACAGCCGCGTGCTCTGGCTCGTATCCACCTGGTTCAGCCGGAACTGGTTCCGCCGCGTACCG CTCTGACCGGTCCGCTGGACGCTCTGAAAGACATCGACCTGCCGCGTGGTTCTCAGTAAAGCAG TGAGACGGCATGCGCGCGC-3'. The resulting plasmid is the psicose biosensor based on pPsiA promoter from *Sinorhizobium fredii* and the PsiR transcription factor from *Sinorhizobium fredii* with mCherry as reporter gene.

Psicose biosensor based on pPsiR promoter from *Sinorhizobium fredii* and the PsiR transcription factor from *Sinorhizobium fredii* with mCherry as reporter gene was constructed in 2 steps:

Step 1: A gBlock fragment containing the pPsiR promoter 5'-GCGGATCCCGAGAAGACAATGGAGG CCGCGCTCCTTGATGCCGACTTGCATGGCGTTGAACCACGGAATGCCGCCGATCTTGACGACCA CGCCGACCTTCGGCGCATCCTGCGCCGCGACGGAAAAGGCACCGGCGAGCGAAAGCGAAGCC GCCAGAGCGGCAGCAAGAAATGTCTTGATCATGTCTCTCCTCCACAGGTTCCCGCAACGGGAAC TCTCCCGGGCGCTGGCGCGCCGGCCTCCAGTTTCAATCCTCCATGACGAGAAGCGCGTTTCCC GCCGCTGACCGCCGCCTCCTCAGACGGCCCAAAGAGCTGCACAATCGATATTTCTTATTTGCAC AATCGATGGTGCAAAGACTATCCTGCTGACTTAATCTTTCGTCAAGCGCAAAACGGCTCTAAGAG ATTCGAGGGGGCTCGGAGCAGAAGTCTTCACCATGGCATATGGC-3' was inserted by Golden Gate with BbsI into the Universal Biosensing Chassis.

Step 2: The plasmid obtained at step 1 was assembled by Golden Gate with BsmBI to a gBlock fragment containing the PsiR gene 5'-GCAGCGCCTCGAGCGTCTCATGGAAGAGACGGTACAAAGA GGAGAAATACCATATGGCTAACTCTGGTAAAAAAAAAGCTACCATCTACGACCTGTCTGTTCTGT CTGGTTCTTCTCCGTCTACCGTTTCTGCTGTTCTGAACGGTACCTGGCGTAAACGTCGTATCAAA GAATCTACCGCTGAACTGATCCGTAACCTGGCTGAAACCCACCAGTACACCGCTAACCGTCAGG CTCGTGGTCTGCGTTCTTCTCGTTCTGGTCTGGTTGGTCTGCTGCTGCCGGTTCACGACAACCG TTACTTCTCTTCTCTGGCTCAGACCTTCGAAGCTCACGTTCGTTCTAAAGGTCAGTGCCCGATCG TTGTTTCTGCTTCTCGTGACCCGCAGGAAGAACGTAAAACCGCTGAAACCCTGATCTCTTACTCT ATCGACGAACTGTTCATCTGCGGTGCTACCGACCCGGACGGTGTTCACGAAGTTTGCGAAGCTG CTGGTCTGAAACACATCAACATCGACCTGCCGGGTACCAAAGTTCCGTCTGTTATCTCTGACAAC TTCGAAGGTGGTCGTCTGCTGACCGAAGCTATCATCCGTCACTTCCCGGCTGACCGTGCTCTGG CTCCGACCGACCTGTACCTGTTCGGTGGTCGTAACGACCACGCTTCTCACGAACGTATCCGTGG TTTCCGTGCTGTTAAAAAAGACCTGCTGGGTGACGACCCGGACGAATGCATCCAGCCGACCGGT TACGCTGCTAACAACGCTCGTAAAGCGTTCGAAGCGTTCTACGCTCGTCACGGTAAACTGCCGC GTGGTCTGTTCGTTAACTCTTCTATCAACTTCGAAGGTCTGCTGCGTTTCATGGCTGAACACCCG CACGACAACTTCACCGACCTGGTTGTTGGTTGCTACGACTACGACCCGTTCGCTTCTTTCCTGCC GTTCCCGGTTATCATGATCCGTCAGGACGTTGAAGGTATGATCGCTAAAGCGTTCGAAGTTATCG AACAGCCGCGTGCTCTGGCTCGTATCCACCTGGTTCAGCCGGAACTGGTTCCGCCGCGTACCG

CTCTGACCGGTCCGCTGGACGCTCTGAAAGACATCGACCTGCCGCGTGGTTCTCAGTAAAGCAG TGAGACGGCATGCGCGCGC-3'. The resulting plasmid is the psicose biosensor based on pPsiR promoter from *Sinorhizobium fredii* and the PsiR transcription factor from *Sinorhizobium fredii* with mCherry as reporter gene.

Psicose biosensor based on pPsiA promoter from *Sinorhizobium meliloti* and the PsiR transcription factor from *Sinorhizobium meliloti* with mCherry as reporter gene was constructed in 2 steps:
Step 1: A gBlock fragment containing the pPsiA promoter 5'-GCGGATCCCGAGAAGACAATGGACG GTGCTTTCCTTGATCCGTCGCTTGCGCCACGTGCCGTTTAGCACCGCACTGACGGTAGAGGGCG AACTTCCCGACAGCACCGAGAGATCATAGATCGTCGCTTTTTTCCTGCCGCCGTTCGTCATCTGA CCTCCTCCAAACCCCGGAAAACCGATGCGCACGTTTCCTGGAATTGCTCTAGTGCCGATTTCGG CTTGACGAAAGATTAAGTCTGAATGATAGTCATTGCACCATCGATTGTGCAAAAAAGAAATATCGA TTGTGCAAGTTGTTGGTGCCGTCTGAGGAGGCGGCCGTCAGCGGCGGGATATCCCCTTCCGTG CAAAAGAATTAAGCTGGAGGCCGGCGCGTGAAGCGCCCGGGAGCGTTCCCCTCGGGGAAACAT GTGGAGGAGAAACAGCAGAAGTCTTCACCATGGCATATGGC-3' was inserted by Golden Gate with BbsI into the Universal Biosensing Chassis.
Step 2: The plasmid obtained at step 1 was assembled by Golden Gate with BsmBI to a gBlock fragment containing the PsiR gene 5'-GCAGCGCCTCGAGCGTCTCATGGAAGAGACGGTACAAAGA GGAGAAATACCATATGACCAACGGTGGTCGTAAAAAAGCTACCATCTACGACCTGTCTGTTCTGT CTGGTTCTTCTCCGTCTACCGTTTCTGCTGTTCTGAACGGTACCTGGCGTAAACGTCGTATCAAA GAATCTACCGCTGAACTGATCCGTTCTCTGGCTGAAACCCACCAGTACACCGCTAACCGTCAGG CTCGTGGTCTGCGTTCTTCTCGTTCTGGTCTGGTTGGTCTGCTGCTGCCGGTTCACGACAACCG TTACTTCTCTTCTCTGGCTCAGACCTTCGAAGCTCACGTTCGTTCTAAAGGTCAGTGCCCGATCG TTGTTTCTGCTTCTCGTGACCCGGAAGAAGAACGTCGTACCGCTGAAACCCTGATCTCTTACTCT ATCGACGAACTGTTCATCTGCGGTGCTACCGACCCGGACGGTGTTCACGAAGTTTGCGAAGCTG CTGGTCTGCGTCACATCAACATCGACCTGCCGGGTACCAAAGTTCCGTCTGTTATCTCTGACAAC TTCGAAGGTGGTCGTCTGCTGACCGAAGCTATCATCCGTCACTTCCCGGCTGAACGTCCGCTGG AACCGGACGACCTGTACCTGTTCGGTGGTCGTGACGACCACGCTACCCGTGAACGTATCCGTG GTTTCCGTGCTGTTAAATCTGACCTGCTGGGTGCTGACCCGGACGAATGCATCTGGCCGACCGG TTACGCTGCTGACAACGCTCGTAAAGCGTTCGAAGCGTTCTACGAACAGCACGGTAAACTGCCG CGTGGTTTCTTCGTTAACTCTTCTATCAACTTCGAAGGTCTGCTGCGTTTCATGGCTGAACACCC GCTGGAAAACTTCACCGACCTGGTTGTTGGTTGCTACGACTACGACCCGTTCGCTTCTTTCCTGC CGTTCCCGGTTATCATGATCCGTCAGAACATCGAAGGTATGATCGCTAAAGCGTTCGAAGTTATC GAAGAACCGCGTGCTTCTCTGCAAATCCACATGATCGAACCGCAGCTGGTTCCGCCGCGTACCG CTCTGACCGGTCCGCTGGACGCTCTGATGGACTCTGAAATGCCGCGTGAATAAAGCAGTGAGAC GGCATGCGCGCGC-3'. The resulting plasmid is the psicose biosensor based on pPsiA promoter from *Sinorhizobium meliloti* and the PsiR transcription factor from *Sinorhizobium meliloti* with mCherry as reporter gene.

Psicose biosensor based on pPsiR promoter from *Sinorhizobium meliloti* and the PsiR transcription factor from *Sinorhizobium meliloti* with mCherry as reporter gene was constructed in 2 steps:
Step 1: A gBlock fragment containing the pPsiR promoter 5'-GCGGATCCCGAGAAGACAATGGATG AACCACGGTATGCCGCCGATCTTGACGACGACACCGACCTTGCCCGTATCCTGCGCCGCGGCG GTATAGGCACCCGCAAGCGAAAGCGACGCCGCCAGAGCGGCGGCAAGAATTTTCTTGATCATGT TTCTCCTCCACATGTTTCCCCGAGGGGAACGCTCCCGGGCGCTTCACGCGCCGGCCTCCAGCT TAATTCTTTTGCACGGAAGGGGATATCCCGCCGCTGACGGCCGCCTCCTCAGACGGCACCAACA ACTTGCACAATCGATATTTCTTTTTTGCACAATCGATGGTGCAATGACTATCATTCAGACTTAATCT TTCGTCAAGCCGAAATCGGCACTAGAGCAATTCCAGGAAACGTGCGCATCGGTTTTCCGGGGTT TGGAGGAGGTCAGAGCAGAAGTCTTCACCATGGCATATGGC-3' was inserted by Golden Gate with BbsI into the Universal Biosensing Chassis.
Step 2: The plasmid obtained at step 1 was assembled by Golden Gate with BsmBI to a gBlock fragment containing the PsiR gene 5'-GCAGCGCCTCGAGCGTCTCATGGAAGAGACGGTACAAAGA GGAGAAATACCATATGACCAACGGTGGTCGTAAAAAAGCTACCATCTACGACCTGTCTGTTCTGT CTGGTTCTTCTCCGTCTACCGTTTCTGCTGTTCTGAACGGTACCTGGCGTAAACGTCGTATCAAA GAATCTACCGCTGAACTGATCCGTTCTCTGGCTGAAACCCACCAGTACACCGCTAACCGTCAGG CTCGTGGTCTGCGTTCTTCTCGTTCTGGTCTGGTTGGTCTGCTGCTGCCGGTTCACGACAACCG

TTACTTCTCTTCTCTGGCTCAGACCTTCGAAGCTCACGTTCGTTCTAAAGGTCAGTGCCCGATCG TTGTTTCTGCTTCTCGTGACCCGGAAGAAGAACGTCGTACCGCTGAAACCCTGATCTCTTACTCT ATCGACGAACTGTTCATCTGCGGTGCTACCGACCCGGACGGTGTTCACGAAGTTTGCGAAGCTG CTGGTCTGCGTCACATCAACATCGACCTGCCGGGTACCAAAGTTCCGTCTGTTATCTCTGACAAC TTCGAAGGTGGTCGTCTGCTGACCGAAGCTATCATCCGTCACTTCCCGGCTGAACGTCCGCTGG AACCGGACGACCTGTACCTGTTCGGTGGTCGTGACGACCACGCTACCCGTGAACGTATCCGTG GTTTCCGTGCTGTTAAATCTGACCTGCTGGGTGCTGACCCGGACGAATGCATCTGGCCGACCGG TTACGCTGCTGACAACGCTCGTAAAGCGTTCGAAGCGTTCTACGAACAGCACGGTAAACTGCCG CGTGGTTTCTTCGTTAACTCTTCTATCAACTTCGAAGGTCTGCTGCGTTTCATGGCTGAACACCC GCTGGAAAACTTCACCGACCTGGTTGTTGGTTGCTACGACTACGACCCGTTCGCTTCTTTCCTGC CGTTCCCGGTTATCATGATCCGTCAGAACATCGAAGGTATGATCGCTAAAGCGTTCGAAGTTATC GAAGAACCGCGTGCTTCTCTGCAAATCCACATGATCGAACCGCAGCTGGTTCCGCCGCGTACCG CTCTGACCGGTCCGCTGGACGCTCTGATGGACTCTGAAATGCCGCGTGAATAAAGCAGTGAGAC GGCATGCGCGCGC-3’. The resulting plasmid is the psicose biosensor based on pPsiR promoter from *Sinorhizobium meliloti* and the PsiR transcription factor from *Sinorhizobium meliloti* with mCherry as reporter gene.

Psicose biosensor based on pPsiA promoter from *Agrobacterium tumefaciens* and the PsiR transcription factor from *Agrobacterium tumefaciens* with mEmerald as reporter gene and a downstream the Mutant Drop Zone was constructed in 3 steps:

Step 1: The psicose biosensor based on pPsiA promoter from *Agrobacterium tumefaciens* and the PsiR transcription factor from *Agrobacterium tumefaciens* with mCherry as reporter gene was used as template in a PCR reaction with the primers 5’-GCGCGGTCTCAGCAGAACTAGTAGCGGCCGCTG CAG-3’ and 5’-GCGCGGTCTCATCCAATTATTTGTACAGTTCGTCC-3’. The PCR product was assembled by Golden Gate with BsaI to a gBlock fragment containing the Mutant Drop Zone 5’-GCGCTGGTCTCATGGATAGCGTGACCGGCGCATCGGTCACGCTATTTGTTGAGGAGAGAGAGC TGTTGACAATTAATCATCGGCTCGTATAATGTGTGGAATTGTGAGCGGATAACAATTGTACAAAGA GGAGAAACTCGAGGATGAGAGACGGATCGATCCGTCTCAAGCGGCATGCCCAGGCATCAAATAA AACGAAAGGCTCAGTCGAAAGACTGGGCCTTTCGTTTTATCTGTTGTTTGTCGGTGAACGCTCTC TACTAGAGTCACACTGGCTCACCTTCGGGTGGGCCTTTCTGCGTTTATAGCAGTGAGACCGC-3’.

Step 2: Due to a design error, the plasmid obtained at step 1, has a BsmBI site between the pTacI promoter and PsiR. This error was corrected by site directed mutagenesis following the Single-Primer Reactions IN Parallel (SPRINP) protocol (3) using the primers 5’-CGAGTGGAAGACTCGGTACAAAG AGG-3’ and 5’-CCTCTTTGTACCGAGTCTTCCACTCG-3’.

Step 3: The plasmid obtained at step 2 was used as template in a PCR reaction with the primers 5’-GCGCGGTCTCATCCATTTGTACCCATGGCTGCTGGC-3’ and 5’-GCGCGGTCTCAGCAGTTGGA TAGCGTGAC-3’ and the PCR product was assembled by Golden Gate with BsaI to a gBlock fragment containing the mEmerald reporter 5’-GCGCGCGGTCTCATGGAGGAAAAGAGGAGAAAAG ATCAATGGTTTCTAAAGGTGAAGAACTGTTCACCGGTGTTGTTCCGATCCTGGTTGAACTGGACG GTGACGTTAACGGTCACAAATTCTCTGTTTCTGGTGAAGGTGAAGGTGACGCTACCTACGGTAAA CTGACCCTGAAATTCATCTGCACCACCGGTAAACTGCCGGTTCCGTGGCCGACCCTGGTTACCA CCCTGACCTACGGTGTTCAGTGCTTCGCTCGTTACCCGGACCACATGAAACAGCACGACTTCTT CAAATCTGCTATGCCGGAAGGTTACGTTCAGGAACGTACCATCTTCTTCAAAGACGACGGTAACT ACAAAACCCGTGCTGAAGTTAAATTCGAAGGTGACACCCTGGTTAACCGTATCGAACTGAAAGGT ATCGACTTCAAAGAAGATGGTAACATCCTGGGTCACAAACTGGAATACAACTACAACTCTCACAA AGTTTACATCACCGCTGACAAACAGAAAAACGGTATCAAAGTTAACTTCAAAACCCGTCACAACAT CGAAGATGGTTCTGTTCAGCTGGCTGACCACTACCAGCAGAACACCCCGATCGGTGACGGTCCG GTTCTGCTGCCGGACAACCACTACCTGTCTACCCAGTCTAAACTGTCTAAAGACCCGAACGAAAA ACGTGACCACATGGTTCTGCTGGAATTTGTTACCGCTGCTGGTATCACCCTGGGTATGGACGAA CTGTACAAATAAGAGAGCAGTGAGACCGCGCGC-3’. The resulting plasmid is the psicose biosensor based on pPsiA promoter from *Agrobacterium tumefaciens* and the PsiR transcription factor from *Agrobacterium tumefaciens* with mEmerald as reporter gene and a downstream the Mutant Drop Zone.

Psicose biosensor based on pPsiA promoter from *Agrobacterium tumefaciens* and the PsiR transcription factor from *Agrobacterium tumefaciens* with mEmerald as reporter gene and a downstream D-Psicose 3-epimerase (DPEase) from *Clostridium cellulolyticum* under the control of pTacI promoter was constructed in 1 step:

Step 1: A gBlock fragment containing the pTacI promoter followed by the *C. cellulolyticum* DPEase 5'-GCTACGATCTGGTCTCATGGAATTCGCGGCCGCTTCTAGAGAGGAGCTGTTGACAATTAATCATC GGCTCGTATAATGTGTGGAATTGTGAGCGGATAACAATTTTAACTTTAAGAAGGAGATATACAAAT GAAACACGGTATCTACTACGCTTACTGGGAACAGGAATGGGAAGCTGACTACAAATACTACATCG AAAAAGTTGCTAAACTGGGTTTCGACATCCTGGAAATCGCTGCTTCTCCGCTGCCGTTCTACTCT GACATCCAGATCAACGAACTGAAAGCTTGCGCTCACGGTAACGGTATCACCCTGACCGTTGGTC ACGGTCCGTCTGCTGAACAGAACCTGTCTTCTCCGGACCCGGACATCCGTAAAAACGCTAAAGC TTTCTACACCGACCTGCTGAAACGTCTGTACAAACTGGACGTTCACCTGATCGGTGGTGCTCTGT ACTCTTACTGGCCGATCGACTACACCAAAACCATCGACAAAAAAGGTGACTGGGAACGTTCTGTT GAATCTGTTCGTGAAGTTGCTAAAGTTGCTGAAGCTTGCGGTGTTGACTTCTGCCTGGAAGTTCT GAACCGTTTCGAAAACTACCTGATCAACACCGCTCAGGAAGGTGTTGACTTCGTTAAACAGGTTG ACCACAACAACGTTAAAGTTATGCTGGACACCTTCCACATGAACATCGAAGAAGACTCTATCGGT GGTGCTATCCGTACCGCTGGTTCTTACCTGGGTCACCTGCACACCGGTGAATGCAACCGTAAAG TTCCGGGTCGTGGTCGTATCCCGTGGGTTGAAATCGGTGAAGCTCTGGCTGACATCGGTTACAA CGGTTCTGTTGTTATGGAACCGTTCGTTCGTATGGGTGGTACCGTTGGTTCTAACATCAAAGTTT GGCGTGACATCTCTAACGGTGCTGACGAAAAAATGCTGGACCGTGAAGCTCAGGCTGCTCTGGA CTTCTCTCGTTACGTTCTGGAATGCCACAAACACTCTTAATACTAGTAGCGGCCGCTGCAGTGAG ACCGCTACGATC-3' was used as template in a PCR reaction with the primers 5'-GCCGTCTCGG ATGAAACACGGTATCTACTAC-3' and 5'-GCCGTCTCCCGCTTTAAGAGTGTTTGTGGCATTC-3' and the PCR product was inserted by Golden Gate with BsmBI in the psicose biosensor based on pPsiA promoter from *Agrobacterium tumefaciens* and the PsiR transcription factor from *Agrobacterium tumefaciens* with mEmerald as reporter gene and a downstream the Mutant Drop Zone. The resulting plasmid is the Psicose biosensor based on pPsiA promoter from *Agrobacterium tumefaciens* and the PsiR transcription factor from *Agrobacterium tumefaciens* with mEmerald as reporter gene and a downstream D-Psicose 3-epimerase (DPEase) from *Clostridium cellulolyticum* under the control of pTacI promoter.

D-Psicose 3-epimerase (DPEase) from *Clostridium cellulolyticum* under the control of pTacI promoter was constructed in 1 step:
Step 1: The pSB1C3 backbone was used as template in a PCR reaction with the primers 5'-GCGGTCTCTGCAGTCCGGCAAAAAAGGGCAAGG-3' and 5'-GCGGTCTCTTCCAGAAATCATCC TTAGCG-3' and the PCR product was assembled by Golden Gate with BsaI to a gBlock fragment containing the pTacI promoter followed by the *C. cellulolyticum* DPEase 5'-GCTACGATCTGGTCTCA TGGAATTCGCGGCCGCTTCTAGAGAGGAGCTGTTGACAATTAATCATCGGCTCGTATAATGTGTG GAATTGTGAGCGGATAACAATTTTAACTTTAAGAAGGAGATATACAAATGAAACACGGTATCTACT ACGCTTACTGGGAACAGGAATGGGAAGCTGACTACAAATACTACATCGAAAAAGTTGCTAAACTG GGTTTCGACATCCTGGAAATCGCTGCTTCTCCGCTGCCGTTCTACTCTGACATCCAGATCAACGA ACTGAAAGCTTGCGCTCACGGTAACGGTATCACCCTGACCGTTGGTCACGGTCCGTCTGCTGAA CAGAACCTGTCTTCTCCGGACCCGGACATCCGTAAAAACGCTAAAGCTTTCTACACCGACCTGCT GAAACGTCTGTACAAACTGGACGTTCACCTGATCGGTGGTGCTCTGTACTCTTACTGGCCGATC GACTACACCAAAACCATCGACAAAAAAGGTGACTGGGAACGTTCTGTTGAATCTGTTCGTGAAGT TGCTAAAGTTGCTGAAGCTTGCGGTGTTGACTTCTGCCTGGAAGTTCTGAACCGTTTCGAAAACT ACCTGATCAACACCGCTCAGGAAGGTGTTGACTTCGTTAAACAGGTTGACCACAACAACGTTAAA GTTATGCTGGACACCTTCCACATGAACATCGAAGAAGACTCTATCGGTGGTGCTATCCGTACCGC TGGTTCTTACCTGGGTCACCTGCACACCGGTGAATGCAACCGTAAAGTTCCGGGTCGTGGTCGT ATCCCGTGGGTTGAAATCGGTGAAGCTCTGGCTGACATCGGTTACAACGGTTCTGTTGTTATGGA ACCGTTCGTTCGTATGGGTGGTACCGTTGGTTCTAACATCAAAGTTTGGCGTGACATCTCTAACG GTGCTGACGAAAAAATGCTGGACCGTGAAGCTCAGGCTGCTCTGGACTTCTCTCGTTACGTTCT GGAATGCCACAAACACTCTTAATACTAGTAGCGGCCGCTGCAGTGAGACCGCTACGATC-3'. The resulting plasmid is the D-Psicose 3-epimerase (DPEase) from *Clostridium cellulolyticum* under the control of pTacI promoter.

Wild-type D-psicose 3-epimerase (DPEase) from *Clostridium cellulolyticum* with a C-terminal Histidine tag under the control of pTacI promoter was constructed in 1 step:
Step 1: The D-Psicose 3-epimerase (DPEase) from *Clostridium cellulolyticum* under the control of pTacI promoter was used as template in a PCR reaction with the primers 5'-GCGGTCTCACCATCAC CACCACTAATACTAGTAGCGGCCGCTGCA-3' and 5'-GCGGTCTCGATGGTGGTGCTCGAGAGAG TGTTTGTGGCATTCCAG-3' and the PCR product was self assembled by Golden Gate with BsaI.

The resulting plasmid is the wild-type D-psicose 3-epimerase (DPEase) from *Clostridium cellulolyticum* with a C-terminal Histidine tag under the control of pTacI promoter.

Mutant A142N of D-psicose 3-epimerase (DPEase) from *Clostridium cellulolyticum* with a C-terminal Histidine tag under the control of pTacI promoter was constructed in 1 step:
Step 1: The psicose biosensor based on pPsiA promoter from *Agrobacterium tumefaciens* and the PsiR transcription factor from *Agrobacterium tumefaciens* with mEmerald as reporter gene and a downstream D-Psicose 3-epimerase (DPEase) from *Clostridium cellulolyticum* (mutant A142N) under the control of pTacI promoter was used as template in a PCR reaction with the primers 5'-GCGGTCTCGATGGTGGTGCTCGAGAGAGTGTTTGTGGCATTCCAG-3' and 5'-GCGGTCTCAGG AGATATACAAATGAAACACGGTATCTACTAC-3'. A second PCR reaction was conducted in parallel using the primers 5'-GCGGTCTCACCATCACCACCACTAATACTAGTAGCGGCCGCTGCA-3' and 5'-GCGGTCTCTCTCCTTCTTAAAGTTAAAATTGTTATCCGCTCACAATTCC-3' and as template the D-Psicose 3-epimerase (DPEase) from *Clostridium cellulolyticum* under the control of pTacI promoter. The two PCR products were assembled by Golden Gate with BsaI. The resulting plasmid is the mutant A142N of D-psicose 3-epimerase (DPEase) from *Clostridium cellulolyticum* with a C-terminal Histidine tag under the control of pTacI promoter.

Supplementary references

1. Mu,W., Chu,F., Xing,Q., Yu,S., Zhou,L. and Jiang,B. (2011) Cloning, expression, and characterization of a D-psicose 3-epimerase from Clostridium cellulolyticum H10. *J. Agric. Food Chem.*, **59**, 7785–7792.
2. Rose,A.S., Bradley,A.R., Valasatava,Y., Duarte,J.M., Prlic,A. and Rose,P.W. (2018) NGL viewer: web-based molecular graphics for large complexes. *Bioinforma. Oxf. Engl.*, **34**, 3755–3758.
3. Engler,C., Kandzia,R. and Marillonnet,S. (2008) A one pot, one step, precision cloning method with high throughput capability. *PloS One*, **3**, e3647.
